# Complexity of genetic mechanisms conferring nonuniformity of recombination in maize

**DOI:** 10.1038/s41598-017-01240-2

**Published:** 2017-04-26

**Authors:** Qingchun Pan, Min Deng, Jianbing Yan, Lin Li

**Affiliations:** National Key Laboratory of Crop Genetic Improvement, Huazhong Agricultural University, Wuhan, 430070 China

## Abstract

Recombinations occur nonuniformly across the maize genome. To dissect the genetic mechanisms underlying the nonuniformity of recombination, we performed quantitative trait locus (QTL) mapping using recombinant inbred line populations. Genome-wide QTL scan identified hundreds of QTLs with both *cis-prone* and *trans-* effects for recombination number variation. To provide detailed insights into *cis-* factors associated with recombination variation, we examined the genomic features around recombination hot regions, including density of genes, DNA transposons, retrotransposons, and some specific motifs. Compared to recombination variation in whole genome, more QTLs were mapped for variations in recombination hot regions. The majority QTLs for recombination hot regions are *trans-*QTLs and co-localized with genes from the recombination pathway. We also found that recombination variation was positively associated with the presence of genes and DNA transposons, but negatively related to the presence of long terminal repeat retrotransposons. Additionally, 41 recombination hot regions were fine-mapped. The high-resolution genotyping of five randomly selected regions in two F_2_ populations verified that they indeed have ultra-high recombination frequency, which is even higher than that of the well-known recombination hot regions *sh1-bz* and *a1-sh2*. Taken together, our results further our understanding of recombination variation in plants.

## Introduction

Recombination refers to the phenomenon of genomic exchange among chromatids, which leads to new alleles and new combinations of existing alleles^1–3^. Together with DNA mutation, recombination is a key driving force in genome evolution and can enhance the genetic diversity of species^4–6^. In crops, recombination contributes substantially to breeding by the rearrangement of genomic fragments, which results in the introduction of new and improved allele combinations as well as the elimination of deleterious mutations^7, 8^. Recombination events do not occur uniformly along the chromosomes, which may be affected by genetic factors and specific DNA sequences^9–11^. In animals, discovery of the genetic and genomic patterns furthered our understanding of recombination^12–14^.

Previous studies suggested variation in recombination frequency is under both *cis*- and *trans*- genetic control^15–19^. Several genes that regulate recombination have been identified. In humans, PR domain containing 9 (*PRDM9*) locus has been identified as a *trans*- regulator of recombination hot spots^17^. The *phs1* and *rad51* genes were reported to affect recombination variation at the whole genome scale in maize^20–22^. Besides *trans*-acting genes, *cis-* genomic features were also found to regulate recombination. Study of different *PRDM9* alleles found that recombination break sites tend to cluster near transcript start sites (TSS) and simple repeat regions, and are associated with DNA motifs such as CCTCCCT and CCNCCNTNNCCNC^17, 19^. Gene density and CpG islands have also been reported to be positively correlated with recombination rate^23, 24^. In maize, a putative recombination hot region was reported to lie in an unusual gene-rich region including the *Bz*-*a1* locus^25^. Although gene density is positively correlated with recombination frequency, certain transposons (TE) were found to be negatively correlated with recombination^26^. Using recombination crossover number as a quantitative trait, quantitative trait locus (QTL) mapping method was employed to dissect the genetic mechanism underlying recombination variation in maize segregating population, and several recombination QTLs were identified^27^. These studies suggest that the regulation of recombination is very complex in both animals and plants.

As a model for genetic studies, maize has a genome of 2.3 Gb and more than 85% of the genome is composed of transposons and other repetitive sequences^28–31^. The complex genomic composition of maize may affect recombination patterns^32–34^. Two putative recombination hot regions (*a1-sh2* and *sh1-bz*) were identified 20 years ago, indicative of the nonuniform distribution of recombination events across maize genome^35, 36^. Further studies of these two recombination hot spots showed that genes and specific genomic contents were significantly enriched or depleted around them^37–39^. Recent studies have uncovered that recombination hot regions were related to phenotypic and gene expression variations, and different maize subgroups exhibited different genome-wide patterns in maize^40, 41^. However, the underlying genetic mechanisms of recombination variation in maize have not been completely explored.

Here, we conducted QTL mapping for recombination event number using 11 recombination inbred line (RIL) populations that have been genotyped with ~50000 SNPs^41^. We identified both *cis-* and *trans-* QTLs. We found candidate genes for some *trans-* QTLs. We also identified tens of genomic features as the *cis*- factors that are associated with the variation of recombination frequency. The *cis*- and *trans*- genetic and genomic factors were further validated in 23 DH populations and a panel of natural inbred lines^40, 42^. Furthermore, based on the identified features that are associated with recombination, we identified 41 putative recombination hot regions and 5 of them were verified in two separate F_2_ populations. Our results provide a comprehensive genome-wide scan of genetic and genomic factors conferring maize recombination variation, which will further our understanding of recombination and aid breeding in maize.

## Results

### Both *cis-* elements and *trans-* factors contribute to the nonuniformity of recombination events

**Figure 1:**
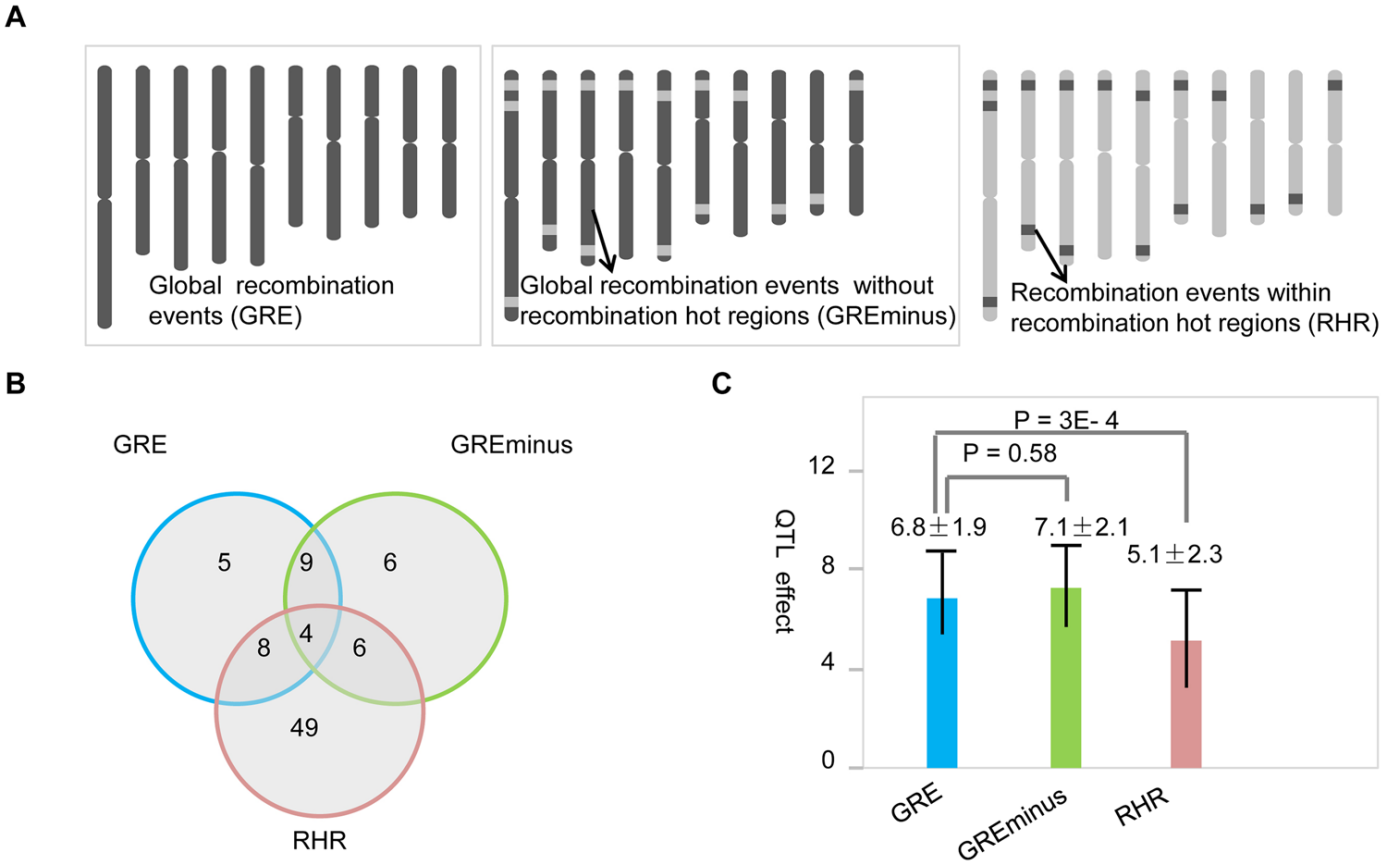
QTL mapping of recombination number variation in maize. (**A**) The schematic diagram of global recombination events (GRE, left panel), global recombination events substracting recombination number in recombination hot regions (GREminus, middle panel), and recombination event in recombination hot regions (RHR, right panel). Recombination number from dark genomic regions was counted for QTL mapping, while the ones from grey regions were excluded. (**B**) Venn Diagram of QTL mapping results among GRE, GREminus and RHR. (**C**) QTL effect variation among GRE, GREminus and RHR.

Our previous study has provided the genome-wide landscape of recombination events across 11 RIL populations and one teosinte-maize introgression population, and identified 143 recombination hot regions where significantly more recombination events were identified than genome-wide average, indicative of nonuniformity of recombination in maize genome^41^. To decipher the genetic mechanisms conferring the nonuniformity of recombination events across maize genome, we considered the global recombination event number of all chromosomes (GRE) and the recombination number of each chromosome (GREchr) as phenotypic traits to perform QTL mapping across 11 RILs (Fig. 1A). GRE could indicate global recombination rate of each segregation population, while GREchr would be more accurate to quantify recombination variation for each chromosome. Totally, we mapped 26 QTLs for GRE in 11 populations. These QTLs were distributed on six chromosomes except chromosomes 6, 8, 9 and 10 (Table S1). For GREchr, a total of 245 QTLs were identified. The QTL number for GREchr ranged from 15 (for chromosome 1) to 30 (for chromosome 2) (Table 1). The highest number of recombination QTLs (39) were detected in population ZONG3/YU87-1, while the least (16) were identified in population KUI3/BY815 (Table S1). Meanwhile, an average of 16.1% of recombination number variation could be explained by recombination QTLs for GREchr, which is lower than an average of 21.2% for GRE. These may be due to that GRE QTLs confer larger recombination variation, while GREchr QTLs somehow partitioned the global recombination variation into different parts in each chromosome.

**Table 1:** Summary of *cis*- and *trans*- QTL mapping results in 11 RIL and 23 DH populations.

Trait	RIL		DH	
	*Cis-prone*	*Trans*	*Cis-prone*	*Trans*
Chr1	2	13	12	24
Chr2	8	22	9	18
Chr3	3	21	26	25
Chr4	13	16	13	34
Chr5	13	9	15	25
Chr6	11	14	7	27
Chr7	7	19	17	25
Chr8	0	21	9	29
Chr9	0	27	10	20
Chr10	7	19	4	25
Sum^a^	64	181	122	252

^a^is the total number of QTL for each type (*cis-prone*, *trans*) across the ten chromosomes.

For GREchr, if QTL is located on the same chromosome, of which the recombination number variation was dissected, the QTL was defined as *cis-prone* QTL, otherwise it was defined as *trans-* QTL (see methods). Of the 245 QTLs underlying GREchr detectected in the 11 RIL populations, 64 were *cis-prone* QTLs, and 181 were *trans-*QTLs (Table 1). These 64 *cis-prone* QTLs were distributed on 8 maize chromosomes except chromosomes 8 and 9, while *trans-*QTLs were located on all maize chromosomes (Fig. S1). Recombination *cis-prone* QTLs had larger effects than *trans-*QTLs (P = 9.24E-13; Fig. S1). Of the *cis-prone* QTLs, the one located on chromosome 2 could explain up to 18.5% of the recombination variation of chromosome 2 (Table S1).

To validate our QTL mapping results for GREchr, we also mapped QTLs for recombination variation across 23 DH populations, which were constructed from 22 European maize inbred lines belonging to the Dent and Flint gene pools^40^. Comparable number and effects of *cis-prone* and *trans-* recombination QTLs were identified as in our 11 RIL populations (Table 1; Fig. S2; Table S2). Interestingly, of these 64 *cis-prone* recombination QTLs detected in RILs, 37 (58%) were also mapped in DH populations, and 161 out of 181 (89%) *trans-* QTL identified in our RIL populations could be detected in DHs as well, suggesting that similar genetic factors were employed to control recombination in these two different types of population.

To further dissect the recombination mechanisms that are related to the formation of recombination hot regions, we did QTL mapping for recombination events that occurred only outside recombination hot regions (GREminus), and recombination events that solely occurred within recombination hot regions (RHR) (Fig. 1A), and compared the QTL mapping results between GRE, GREminus, and RHR. In total, we mapped 26 and 25 recombination QTLs for GRE and GREminus, respectively. Comparative analyses between GRE and GREminus showed that 13 QTLs were shared between the two traits, while nearly half of the recombination QTLs were specific for each recombination trait (Fig. 1B). For the remaining 12 QTLs that are specific to GREminus, 7 out of the 12 specific recombination QTLs were located close to the recombination hot regions (Table S3). Futhermore, QTL mapping of RHR has identified 57 QTLs, of which only 18 were shared by GRE and GREminus (Fig. 1B). Comparative analysis between GRE, GREminus and RHR showed that only four recombination QTLs were detected simultaneously (Fig. 1B). A substantial number (49/67) of recombination QTLs were recombination hot region specific. These hot region specific recombination QTLs indicated that specific genetic mechanism might be involved in the regulation of recombination events in recombination hot regions. Expectedly, 11 QTLs of RHR were located in the same recombination hot regions (*cis-* recombination QTLs), while the others were not (*trans-* recombination QTLs) (Table S4). Interestingly, for RHR, *cis-* recombination QTLs had less effect (3.37%) than *trans-* QTLs (5.54%) (ANOVA; P = 1.22E-2). The QTL effects of RHR was significantly lower (ANOVA; P = 3E-4) than that of GRE and GREminus, while no significant difference was observed between GRE and GREminus (Fig. 1C).

### A substantial number of recombination QTLs co-localized with well-known recombination pathway genes in maize

**Figure 2:**
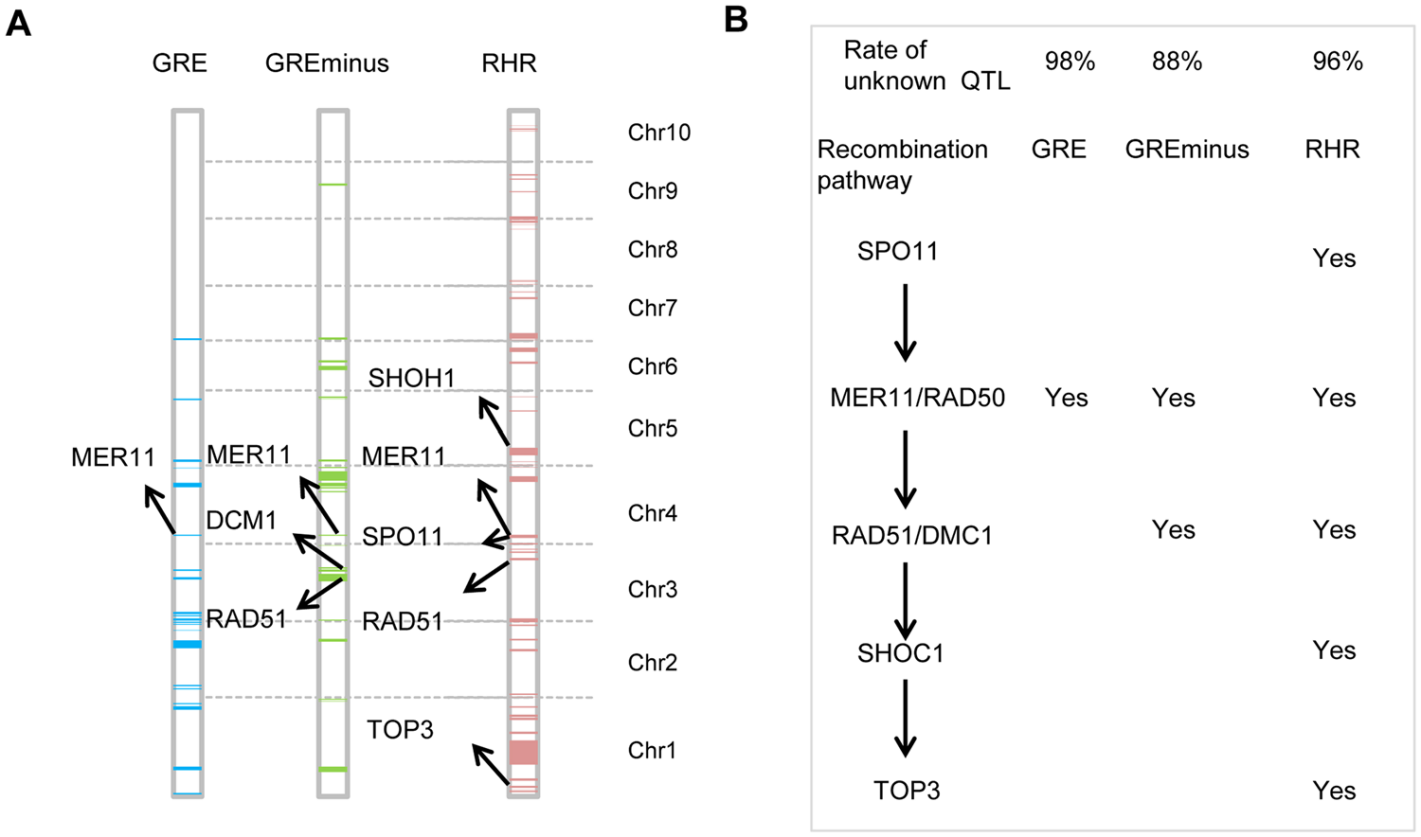
Relationships between recombination QTLs and recombination patyway genes. (**A**) Co-localization of recombination pathway genes with recombination QTLs for GRE, GREminus and RHR. (**B**) Scenario of recombination pathway genes that were detected by the QTL mapping for GRE, GREminus and RHR.

The molecular pathway related to recombination has been well studied, and several genes have already been cloned in *Arabidopsis* ^43^. Through comparative genomics, we identified 30 recombination pathway genes in maize. Co-localization of maize recombination pathway genes with recombination QTLs showed that 1, 3 and 5 recombination pathway genes were located in the QTLs for GRE, GREminus and RHR, respectively (Fig. 2A). Interestingly, *ZmMER11*, which encodes enzyme functioning in the upstream of recombination pathway, was located in the genomic region with detectable QTL for all three traits (GRE, GREminus and RHR). Moreover, two recombination pathway genes (*ZmRAD51* and *ZmDMC1*) were within QTLs for both GREminus and RHR, and two genes (*ZmTOP3* and *ZmSHOH1*) were located in QTLs that were only detected for RHR (Fig. 2B). These results showed that the more recombination QTLs could be mapped for recombination hot regions and the more downstream genes of recombination pathway could be identified through recombination QTL mapping for specific hot regions, suggesting that recombination pathway downstream genes are more likely to be involved in the process of recombination events in recombination hot regions

### Local recombination rate variation is strongly affected by local genomic contents

**Figure 3:**
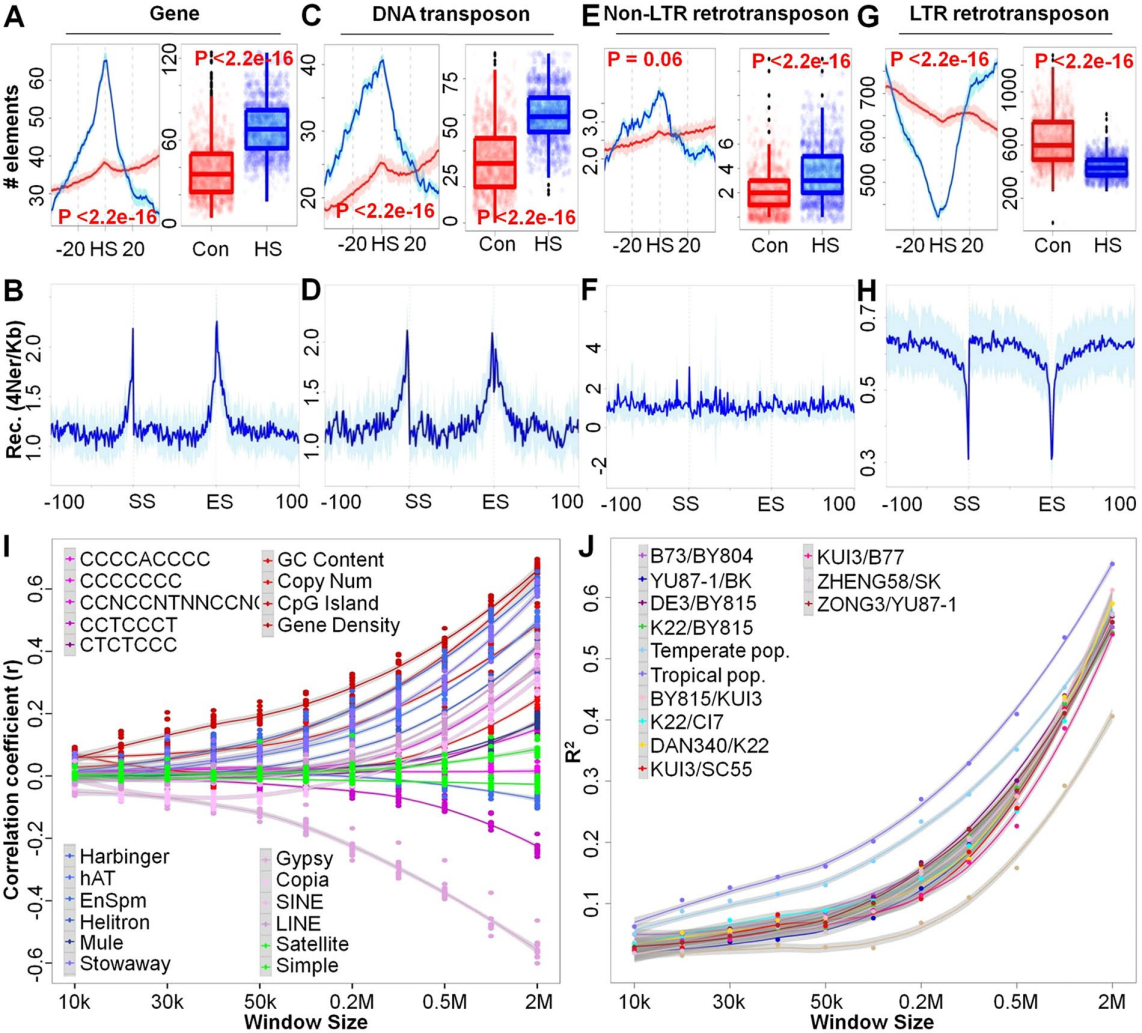
Relationships between recombination rate and local genomic features in maize. (**A**,**C**,**E**,**G**) Distribution of different genome components around recombination hot regions in segregating populations. “HS” is the short name of hot regions, while “Con” shows the random samples used as control. “−20” and “20” represent 20 Mb upstream and downstream genomic regions of hot regions. Blue and red represent recombination hot regions and control samples, respectively. (**B**,**D**,**F**,**H**) Recombination rate (4Ner/Kb) in 1 Kb bins spanning the 100 Kb upstream and downstream regions of different genome components in the natural temperate population from the Hapmap2 dataset. SS and ES indicate the start site and end site of genome content, respectively. (**I**) Correlation coefficients over 11 different sliding window sizes between 21 different genome components and recombination rate in 11 segregation populations. (**J**) The linear regression determination coefficient of genomic components in different window size relative to recombination variation in 11 segregation populations, and in temperate and tropical sub-populations of natural population.

The identification of recombination hot regions and *cis*- QTLs suggests that local chromosome feature might affect the frequency of recombination. To identify these genomic features, we focused on the 143 recombination hot regions and assessed the gene density along the hot regions and their flanking regions^41^. We found that gene density peaks around the hot regions and decreases dramatically away from the hot regions. This pattern is significantly different from that of the randomly selected genomic regions (ANOVA; P < 2.2E-16; Fig. 3A). Gene density (32 genes/Mb) in the hot regions is much higher than the genomic average (16 genes/Mb) (ANOVA; P < 2.2E-16), in accordance with previous reports that crossovers are more likely to occur around gene islands^44, 45^. To complement the analysis of present populations that captured recent recombination events, we also estimated genome-wide recombination that occurred historically in the HapMap2 population^46^. We confirmed that recombination tends to occur near genes. We found that recombination showed a strong tendency to occur at the transcriptional start and 3′-UTR regions of the genes (Fig. 3B). We also observed a significant enrichment of CpG sites in the hot regions (ANOVA; P < 2.2E-16; Fig. S3). Moreover, we uncovered that GC frequency is higher at the start and terminal sites of genes than that of the other genic regions (Fig. S3 **)**. Our findings suggest that recombination rate is increased in the flanking regions of genes rather than within genes.

A substantial proportion of recombination events occurred in intergenic regions^41^, which largely consist of transposable elements^28, 30^. To clarify the relationship between transposons and recombination rate, we first examined DNA transposons and their flanking genomic regions. More than 162,990 putative DNA transposons were identified and classified into six types according to their DNA sequence features (Table S5). These DNA transposons were distributed unevenly across the whole genome (Fig. S4). By comparing the average number of DNA transposons in the flanking genomic regions of the hot regions with that of a set of randomly selected control regions, we found a significant enrichment for five out of the six DNA transposon families, including Helitron, Harbinger, hAT, MULE and Stowaway, in the vicinity of recombination hot regions (ANOVA; P < 2.2E-16; Fig. 3C). However, EnSpm was not enriched, consistent with results that EnSpm is retrotransposon-like in the Triticeae 45 (ANOVA; P = 0.58, Fig. S5).

To further validate the relationship between recombination and DNA transposons, we analyzed recombination variation in DNA transposons and their flanking regions in natural maize populations based on the high density Hapmap2 SNP^42^. We found that all classes of DNA transposons and their flanking regions tend to have higher recombination rates than randomly selected non-genic regions (ANOVA; P < 2.2E-16; Fig. 3D). Intriguingly, recombination rate had a distinct probability peak around both ends of DNA transposons, similar to the distribution of recombination near genes (Fig. 3B). Among all the related DNA transposons, Harbinger exhibits the strongest correlation with recombination not only in the segregating populations but also in the natural populations, while Mule transposon showed the weakest correlation, perhaps due to the high genetic diversity in their terminal inverted repeats (TIRs) compared to all other DNA transposons^47^. Moreover, we also noted that EnSpm and their adjacent regions showed a significant positive relationship with recombination in natural populations (Fig. S5), while such a relationship was not observed in artificial segregating populations.

Genome-wide recombination events are more likely to occur in telomere and decrease toward pericentromeric regions, which mainly consist of retrotransposons, indicative of potential negative relationships between retrotransposons and recombination in maize. To test this hypothesis, we analyzed the density distribution of retrotransposons around recombination hot regions detected in the 11 RIL populations as well as the recombination sites in the natural populations. Plant retrotransposons were classified into two distinct groups: LTR retrotransposons (Copia and Gypsy) and non LTR retrotransposons (LINE and SINE)^47^. For non LTR retrotransposons, we identified enrichment peaks around the hot regions in 11 RIL populations and compared these with the randomly selected genomic regions (ANOVA; P < 2.2E-16; Fig. 3E). We did not see a relationship between non-LTR retrotransposons and recombination rates in natural populations (Fig. 3F). This might be due to the hitchhiking effect of other recombination-related elements in the artificial segregating populations, since we observed that non LTR transposons are more likely to occur in the autosomal regions and are tightly linked with DNA transposons.

For Gypsy LTR retrotransposons, we found a distinct negative correlation (Pearson’s Correlation Test; P < 2.2E-16) with recombination in both artificial segregating populations and natural populations (Fig. S6), indicative of potential repressive regulatory roles of LTR retrotransposons in the process of recombination. Although the adjacent regions of Copia and Gypsy showed significantly lower levels of recombination than other genomic regions (ANOVA; P < 2.2E-16; Fig. 3G,H), this may be related to their closeness to the pericentromere. However, the LTRs of both Copia and Gypsy had low recombination rates (less than 50% of the recombination rate of flanking regions) in natural populations (Fig. 3G,H). The difference of association between non-LTR retrotransposons and LTR retrotransposons with recombination suggested that the recombination repression effects may be related to the presence of LTRs.

To uncover other genetic factors underlying the variation of recombination, we examined the relationship between recombination rate and 21 genomic features (Table S6) using linear regression in a range of sliding window sizes (10 kb, 20 kb, 30 kb, 40 kb, 50 kb, 100 kb, 200 kb, 300 kb, 500 kb, 1 Mb, 2 Mb). As described above, we confirmed that some among the 21 surveyed genomic features, including gene density, gene copy number, CpG island, GC content and DNA transposons, had a high positive correlation with recombination rate variation, while two among the 21 surveyed genomic features, including the Gypsy retrotransposons and microsatellite sequence, were negatively associated with recombination rate in 11 segregating populations. Notably, Copia exhibited a negative correlation with recombination at a fine scale of less than 100 kb, but a positive correlation at a large scale (greater than100 kb) (Pearson’s Correlation Test; P < 0.001; (Fig. 3I; Table S6). This is inconsistent with the observation that Copia showed a negative correlation in natural populations that have a high recombination resolution and a positive correlation in segregating populations that have low recombination resolution (Fig. S7; Tables S6 and S7). We also tried to relate recombination rate to DNA sequence motifs that were found to be associated with recombination in humans^17, 19^. In maize, we found positive correlations of CCCCCCC, CCNCCNTNNCCNC and CCCCACCCC with recombination rate (Tables S6 and S7) which are consistent as in humans^17, 19^. Interestingly, CCTCCCT was negatively associated with recombination rate in our study, while this motif was positively correlated with recombination hot regions in humans^17^ (Tables S6 and S7). These results suggested a possible difference in the mechanism underlying selection of recombination sites between plant and animals. Furthermore, to sum up the potential local effects of genomic contents contributing to the recombination variation in maize, linear regression analysis with an empirical window size identified that the above described associated genomic factors explain up to 60% of the recombination variation over all populations at the 2 Mb genomic window size (Fig. 3J), suggestive of strong *cis-*regulation of recombination variation in maize.

### Tens of novel recombination hot regions were verified in maize

**Figure 4:**
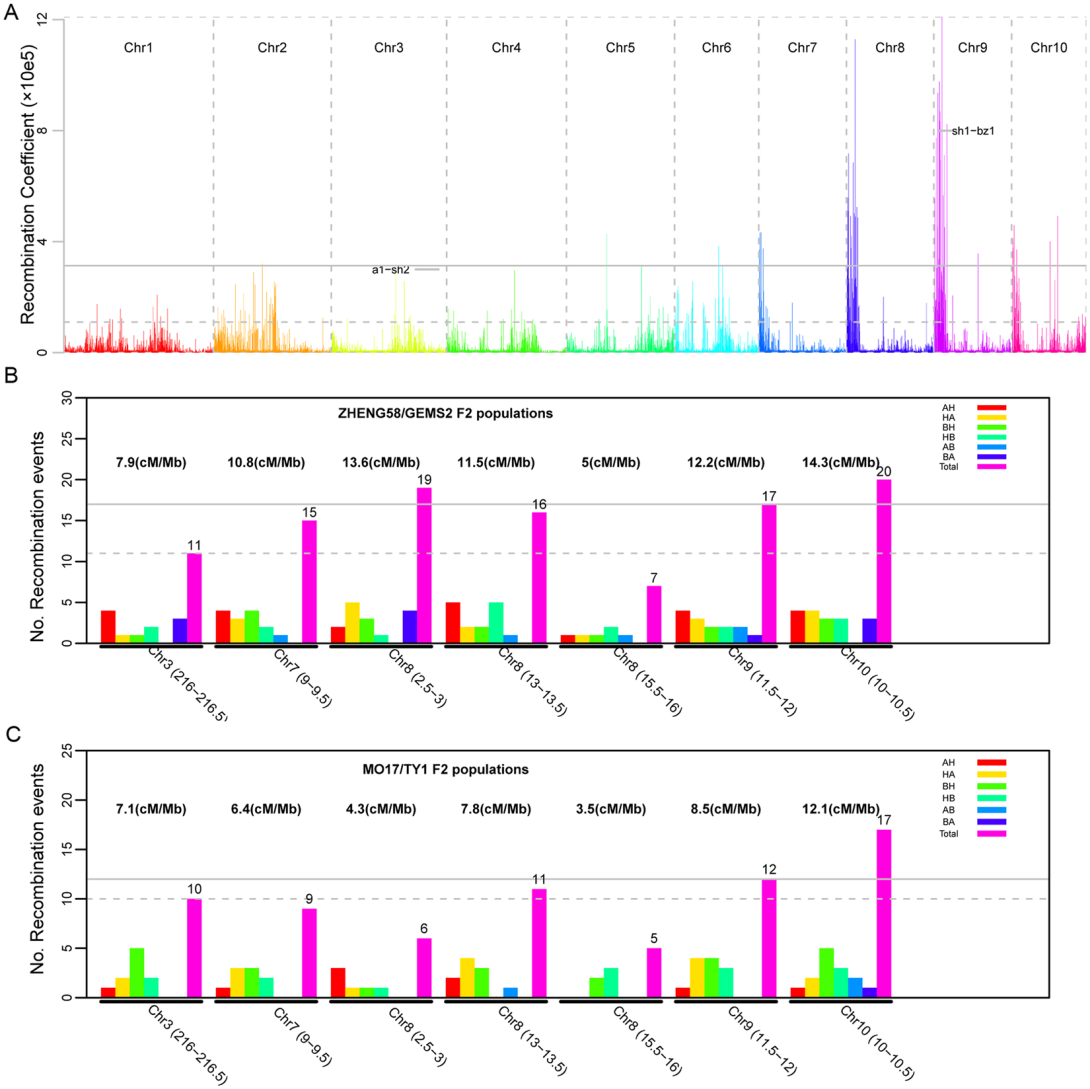
Distribution and validation of novel recombination hot spots in maize. (**A**) Distribution of recombination coefficient indicated a large number of recombination hot spots across the maize genome. Two putative recombination hot spots of *a1-sh2* and *sh1-bz1* were labelled. The gray solid line is the threshold for top 1% recombination coefficients. The Gray dashed line is the threshold line for top 5% recombination coefficients. (**B**) and (**C**) High-density genotypeing validation of five randomly selected recombination hot spots in two F_2_ populations - ZHENG/GEMS2 and Mo17/TY1. A, B, and H in (**B**) and (**C**) graphs represent the genotypes of parent 1, parent 2, and heterozygosity, respectively. The number of crossover was obtained by counting the number of individuals with genotype switching in the target recombination region. For example, AH means there was a genotype switch from parent 1 to heterozygosity. The gray solid line shows the recombination rate at the *sh1-bz1* locus, while Grey dashed line shows the recombination rate of the *a1-sh2* locus.

Previous studies have identified 143 recombination hot regions with 2 Mb window-size, indicative of dramatic recombination frequency variation in maize^41^. Here, we elected to accurately measure the recombination event variation in order to fine-map recombination hot regions by our 11 RIL populations within sliding 500 kb genomic bins. The recombination coefficient analysis (see the Materials and Methods section), which takes local recombination events, genomic contents and the relative distance to centromere of sliding 500 Kb window-size regions into account simultaneously, was employed. Based on the recombination coefficient estimation, we identified 41 genomic regions that showed the highest 1% recombination coefficient values and might be the putative recombination hot regions (Fig. 4A; Table S8). These 41 recombination hot regions are scattered on chromosomes 2, 5, 6, 7, 8, 9 and 10, of which chromosome 9 has most (15), while chromosome 2 and 6 have least (one) (Table S8). These 41 recombination hot regions spanned 0.8% of the maize genome; however, they captured 17.4% of recombination events across different populations^41^. Interestingly, not all the recombination hot regions are located in the ends of chromosomes, 39.1% are also distributed in pericentromeic genomic regions.

There were two well known recombination hot regions (*a1-sh2*, *sh1-bz*) in maize^35, 36^. As expected, one well-known recombination hot regions (*sh1-bz*) was also identified to have top 1% recombination coefficient values across the whole maize genome. Another well-known recombination hot region *(a1-sh2)* in maize was not detected in the hot regions of top 5% coefficient values but captured within top 37%. Using these two well-known recombination hotspots as negative and positive controls, we randomly selected five recombination hot spots with top 1% recombination coefficient values to be verified. These five new recombination hot spots are located in chromosome 7:9–9.5 Mb, 8:2.5–3, 8:13–13.5 Mb, 8:15.5–16 and 10:10–10.5 Mb with an average tract length of 500 Kb. We tested these five novel and two well known (*a1-sh2*, *sh1-bz*) recombination hot spots in two different F2 segregation populations with a population size of 279 and 282, respectively. All the five recombination hot spots harbored exceptional high recombination events in both F_2_ populations (Fig. 4B,C). Of these five recombination hotspots, 8:13–13.5 and 10:10–10.5 Mb had even higher recombination rate than *a1-bz2* in both two populations, and one located in chromosome 10:10–10.5 Mb had higher recombination rate (14.3 and 12.1 cM/Mb) than both *sh1-bz* and *a1-sh2* loci in both F_2_ populations (Fig. 4B,C). Taken together, 41 novel recombination hot spots were identified and five were verified, showed comparable recombination frequency as these two putative well-known recombination hot spots, and could be the new targets of future recombination research.

## Discussion

The genetic mechanisms of recombination events are largely unknown in maize. Only a few genes such as *phs1*, *rad50*, and *rad51* have been identified to affect recombination events through homologous or mutant cloning^20–22^. However, due to the severe defect of these mutations in plants, utilization of these genes was largely limited. In this study, we identified 41 novel recombination hot spots, which confirmed the nonuniformity of recombination frequency across the maize genome. Further, we mapped the variation of recombination events across 11 RIL populations, and found loci that underpin recombination variation in both *cis-* and *trans-* manners. Comparative genomic analysis of recombination-related genes showed a lot of recombination QTLs are coincident with recombination genes, suggestive of the robustness of QTL mapping for the genetic dissection of recombination variation in plants, as suggested by^27^.

### Genomic features are associated with recombination rate variation in maize

In maize, recombination break point shows strong chromosome- and locus-specific patterns^40^. *Cis-* regulation of recombination break point was demonstrated in a series of studies. The interplay of retrotransposon polymorphisms and intragenic recombination was also observed in maize^48^. The maize genome, which is comprised of more than 85% repeat sequences, more than 39,000 protein-coding genes, and ~20,000 long non-coding elements, exhibits extreme complexity^30, 49^. Thus, the lack of high-resolution genetic maps from diverse genetic backgrounds makes the identification of *cis-*elements underlying recombination variation and their effects elusive. In this study, we provided a comprehensive genome-wide scan of 21 genomic features associated with the recombination variation in maize at a high-resolution.

We confirmed the positive correlation between gene content and recombination break point and further identified that recombination break point is more likely to occur in the 5′ and 3′ UTRs of genes, in agreement with reports in mice and *Arabidopsis* ^33, 34^. The recombination pattern along maize genes and their flanking regions appears to be related to chromatin status, such as the DNA methylation levels and histone methylation levels in the 5′ and 3′ UTRs^32, 50, 51^. Open chromatin status may allow easy access to recombination/DSB complexes, and be related to the GC bias at the 5′ and 3′ ends of genes (Fig. S3). It has been reported that GC bias at the 5′ and 3′ UTRs of human genes could form R-loops^52^. R-Loop and its associated DNA nicks may lead to the initiation of DNA breakage and lead to genome instability^53^, which could be associated with recombination.

Interestingly, we found that recombination is positively associated with nearly all DNA transposons and negatively associated with LTR-retrotransposons, in both current and historical populations. The formation of stem-loop structures by TIRs seems to facilitate formation of recombination intermediates^54–56^. LTR-retrotransposons are mainly clustered around the centromeric regions^30^. It has been suggested that LTRs could recruit the DNA binding factor switch-activating protein 1 and block the formation of the replication fork in centromeric regions, enhancing the stability of the genome^57^. It is likely that LTR could use a similar mechanism to block the formation of Holliday structures in the centromere and repress the recombination.

Noteworthily, there is a dramatic structural variation driven by transposons among maize inbreds^31, 58^, possibly reducing the observed correlation between transposons and recombination variation because some transposons may not exist in the non-B73 parental founders, which was used for all analyses. However, structural variation is not likely to change our overall conclusion, because 1) the possibility of structural variation existing in both parents of the segregating populations should be low; 2) similar association trends between transposons and recombination were found in populations developed from non-B73 lines. Although *cis-*effects of genomic features on recombination frequency were found, the overall *cis-*effects account for only ~60% of the variation, indicative of the relevance of other (*trans-*) effects.

### Recombination mechanism in maize may be complex and different from that in other species

It is important to understand mechanisms regulating recombination since it contributes to species evolution^7, 59^. In this study, we used QTL mapping for four different types of recombination events (GRE, GREchr, GREminus and RHR) to capture loci which were associated with recombination variation and identified hundreds of recombination QTLs. Some recombination QTLs were coincident with the well-known recombination related genes^43^, but others were not. This might be caused by either the fact that only a few recombination related genes have been cloned or that maize has different genetic mechanisms underlying recombination variation. Furthermore, through association analysis between genomic features and recombination variations across different genome regions, we found that some specific *cis*- motifs that show different associations with recombination in animals and maize. For example, the CCTCCCT motif^17, 19^ were positively related with recombination rate in animals, but showed negative relationship with recombination variation in maize. All these results indicate that the genetic mechanisms (both *cis*- and *trans*- genetic factors) underlying recombination variation may vary across different species.

### Utilization of recombination patterns in modern maize breeding

Recombination is important in animal and plant breeding because it can create different combinations of alleles^60^. The more the allele combinations, the higher probability of elite hybrids that could be obtained^60^. Although all crop and animal breeding utilized recombination, how to efficiently improve the rate of recombination is still a big challenge. Here, we combined conventional genetic mapping method and association analysis between genomic features and recombination hot regions, not only were *cis-* genetic factors identified, but also hundreds of *trans-* factors were uncovered to explain most of recombination variation in maize. These *trans-* QTLs provide us the selection targets for high recombination inbreds with all favorable recombination alleles. Specifically, our recombination QTL mapping indicated that recombination pathway genes and other unknown genomic loci are both associated with recombination number variation in recombination hotspots. This QTL mapping information could potentially allow us to increase and decrease recombination events in specific genomic regions. Additionally, these *cis-*QTLs mapped in our study could inform us of how many plants or generations will be needed for the improvement of specific genomic regions. All these *cis*- and *trans*- recombination QTL information and the newly-idenfied recombination hot spots will accelerate our progress of maize breeding.

## Methods

### QTL mapping of global recombination variation in maize

In previous study, we’ve constructed 11 recombination inbred line populations -B73/BY804, SK/ZHENG58, BK/YU87-1, K22/CI7, K22/DAN340, KUI3/B77, ZONG3/YU87-1, DE3/BY815, K22/BY815, KUI3/BY815 and KUI3/SC55, and obtained high density linkage maps of these segregating populations^41^. Based on these 11 RIL high-density linkage maps, we could estimate the recombination events by counting the recombination break points. Took the number of recombination evets of all chromosomes (GRE) and each chromosome (GREchr) of each family individuals as phenotype, together with high-density linkage map, we mapped quantitative traits locus (QTL) associated with recombination variation by composite interval mapping. The software Winqtlcart 2.5^61^ was employed. We conducted the 1000 permutations for all recombination traits both in 11 RIL and 23 DH populations using R/qtl permutation test with “scanone” function and “hk” method. The significance of LOD values with 0.05 were obtained. The average LOD cut off value was 2.58 for all recombination traits. To make it more comparable, the genomic region with Likelyhood (LOD) beyond the average LOD score 2.58 was defined as a QTL, and the QTL confidence interval was identified according to genomic range with 1 LOD drop from the LOD peak of the QTL (p < 0.01). To verify our mapping results, 23 DH populations^40^ genotyped by the same SNP platform were also collected to dissect recombination number variation through QTL mapping. For the QTL mapping of recombination variation in each chromosome, if QTL was located in the chromosome, of which the recombination number was analyzed, such QTL was defined as *cis-prone* recombination QTL. If QTL is not mapped to the chromosome, of which the recombination number was analyzed, the QTL was defined as *trans*- recombination QTL.

### QTL mapping of local recombination (recombination hot region depletion and recombination hot region specific) variation

Our previous study has identified 143 recombination hot regions, which harbored a substantial number of recombination events. The total number of recombination and the number of recombination in each chromosome quantify the overall recombination variation across the whole genome or whole chromosome, showing the global recombination variation. The recombination hot spots largely represent the local recombination variation, which might be different from global recombination variation. To test the difference of genetic mechanisms underlying global and local recombination variation, we’ve also conducted QTL mapping on the variation of whole-genome recombination number subtracting the recombination number from recombination hot regions (GREminus), and the QTL mapping for the variation of recombination number from recombination hot regions (RHR). Then, we compared the mapping results between GRE, GREminus and RHR. The QTL, which was mapped to the neighboring region (downstream and upstream 10 Mb) of recombination hot spots, was defined as *cis-*QTL, otherwise it’s *trans-* QTL for RHR.

### Estimation of historical recombination rate in maize natural population

To estimate the historical recombination of maize, we downloaded the HapMap2 SNP dataset^42^, and employed the previously described Markov-Chain Monte Carlo method-Ldhat^62^ to estimate recombination rate in the natural population. The natural population from the HapMap2 dataset^42^ was divided into two subgroups: temperate and tropical sub-groups. The recombination rate value was estimated as 4*N*_*e*_ *r*/kb separately for each chromosome in each population^62^. SNP data in each chromosome was divided into segments of 2,000 SNPs, with an overlap of 500 SNPs according to the physical coordinate in the B73 reference genome. The 4*N*_*e*_ *r*/kb was calculated with a block penalty of 5 and 10 million iterations and a removal burn-in of the first quarter of the samples.

### Estimation of the sequence patterns and genome parameter

We downloaded the B73 V2 reference genome sequence data from the Maizesequence web site http://ftp.maizesequence.org/. In the reference genome of B73, 11 sliding window sizes (10 kb, 20 kb, 30 kb, 40 kb, 50 kb, 100 kb, 200 kb, 300 kb, 500 kb, 1 Mb, and 2 Mb) were used to find distinct sequence motifs. Maize gene number and gene copy number variance (CNV) information was downloaded from the maize genome sequence project (http://ftp.maizesequence.org/ and http://plants.ensembl.org/). CpG islands were predicted as described in previous studies^23^ and the motifs CCTCCCT, CCCCCCC, CTCTCCC, CCCCACCCC, CCNCCNTNNCCNC and GC contents were obtained from an in-house Perl script by parsing the B73 V2 genome sequence. Repetitive sequence features were estimated by the RepeatMasker software downloaded from http://www.repeatmasker.org/ with default parameters. In this study, 12 types of TE-like elements, including Harbinger, MULE, hAT, LINE, Copia, Helitron, EnSpm, Sine, Gypsy, Satellite, Simple, and Stowaway, were annotated.

### Correlation and regression analysis

To study the correlation between recombination rate and genomic patterns, we used 11 different sliding window sizes in an empirical analysis to estimate the correlation value. These windows were defined as 10 kb, 20 kb, 30 kb, 40 kb, 50 kb, 100 kb, 200 kb, 300 kb, 500 kb, 1 Mb, and 2 Mb to verify the stabilitity of the results. For the regression analysis, we used ‘lm’ function in R by treating the genomic patterns as the independent variable, explaining how these patterns contributed to the recombination rate to uncover the relationships between total genomic patterns and recombination rate. For relationships between genomic patterns and recombination rate, R function cor.test was used to do the analysis. The analysis was also conducted in 11 different sliding window sizes. All these analyses were conducted by R 2.15.3 (http://www.r-project.org/).

### Prediction of recombination hotspot

From the 11 RIL populations, we firstly counted the recombination events in a 500k sliding window across the whole genome. Simultaneously taking the number of recombination events, gene density, transposon density and the distance from centromere into account, we used the formula to calculate recombination coefficient (Y) as follows:

1 $${\rm{Y}}=({\rm{X}}\times {\rm{Z}}\times {\rm{M}})/{\rm{N}}$$

where, X is total recombination events; Z is gene desntiy; M is TE density; N is length from centromere (Mb) in (1). Recombination coefficient is a normalized statistic, which is more than 0. The higher the recombination coefficient, the higher the probability of recombination, which make it a robust statistic to identify the recombination hotspot. Accordingly, the genomic regions with recombination coefficient in the top 5 were considered as recombination hotspots. Two F_2_ populations reported in previous study were also used to verify recombination hotspots indefied in this study^63^. Two putatitve recombination hot spots (*a1-sh2*, *sh1-bz1*)^35, 36^ and five new randomly selected recombination hotspots were tested with high-density PCR markers using these two segregating populations^63^. The primer information of these seven recombination hotspots could be found in Table S9.

## Electronic supplementary material

Supplementary tables and figures
