# Supplementary material for: Complexity of genetic mechanisms conferring nonuniformity of recombination in maize
Source: Sci Rep. 2017 Apr 26;7:1205. doi: 10.1038/s41598-017-01240-2 (PMC5430679; doi:10.1038/s41598-017-01240-2)
Supplement: Supplementary file 1 — Supplementary tables and figures [file 41598_2017_1240_MOESM1_ESM.doc]

**Complexity of genetic mechanisms conferring nonuniformity of recombination in maize**

Qingchun Pan^1^, Min Deng^1^, Jianbing Yan^1^ & Lin Li^1*^

* Corresponding author: [hzaulilin@mail.hzau.edu.cn](../../../../C:%5CUsers%5CAdministrator.Sc-201406251427%5CDesktop%5Crecombination_8_30%5Chzaulilin@mail.hzau.edu.cn).

^1^National Key Laboratory of Crop Genetic Improvement, Huazhong Agricultural University, Wuhan 430070, China.

Supplementary material

Fig S1-Fig S7

Table S1-S9


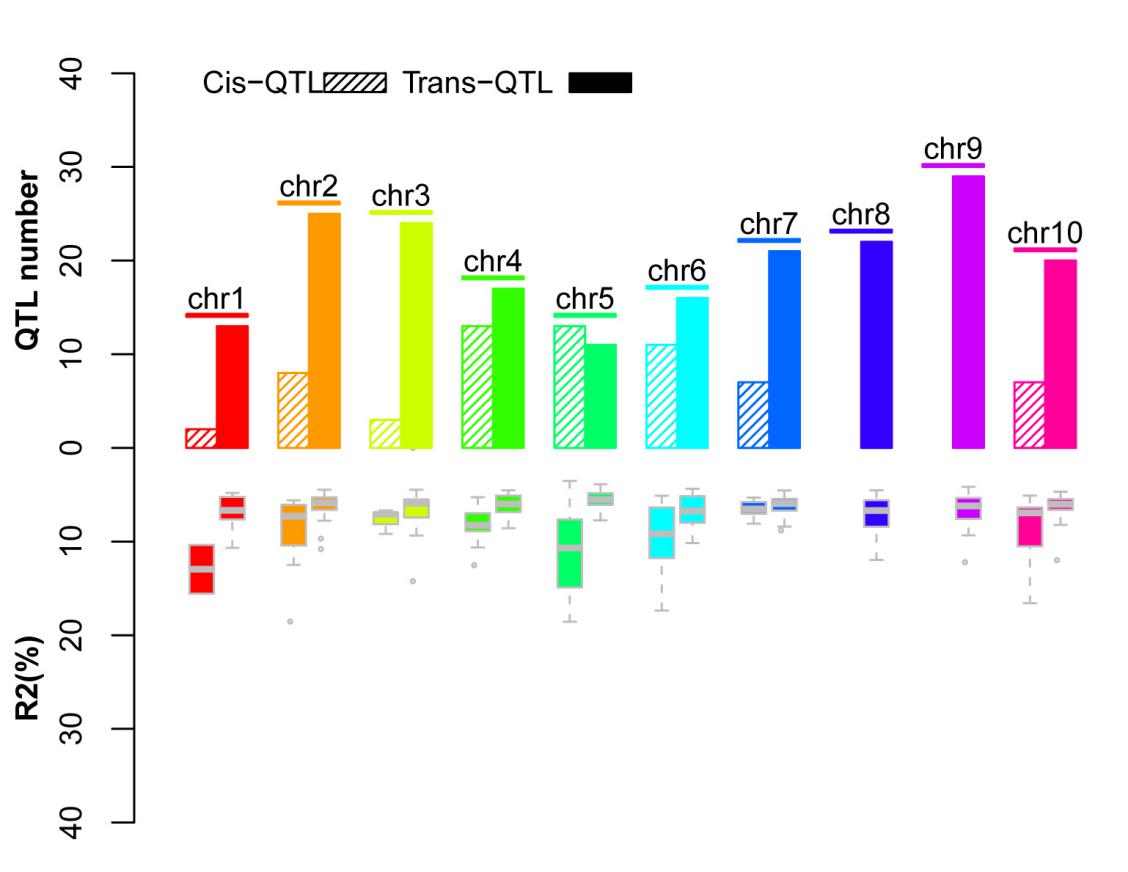


**Supplementary Figure 1**. Distribution of the number and effect of recombination *cis*- and *trans*- QTLs in all 11 RIL populations. Upper is the number of cis-prone and trans- QTLs for each chromosome. Below is the corresponding cis- and trans- QTL effect.

**
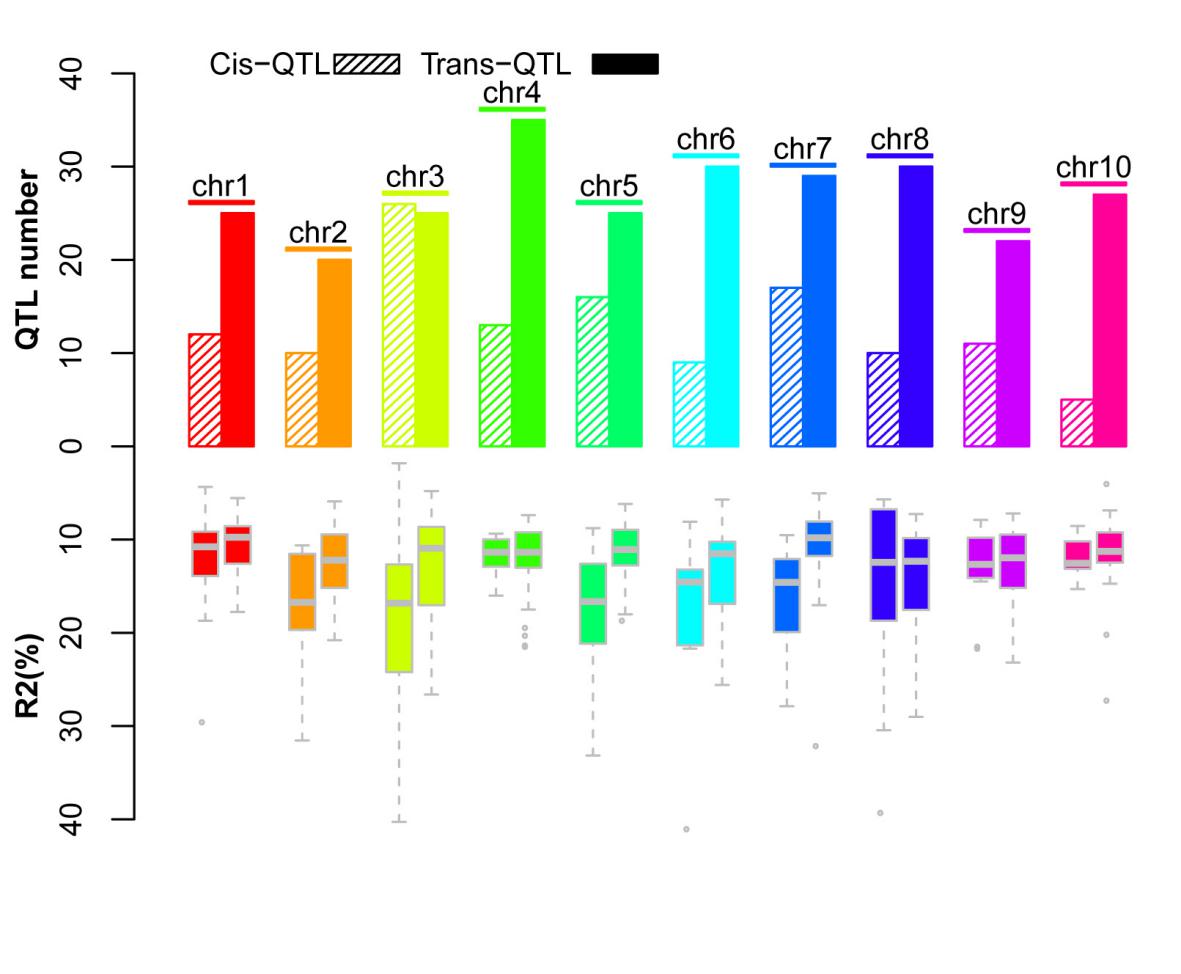
**

**Supplementary Figure 2**. Distribution of the number and effect of recombination *cis*- and *trans*- QTLs in all 23 DH populations. Upper is the number of cis-prone and trans- QTLs for each chromosome. Below is the corresponding cis- and trans- QTL effect.


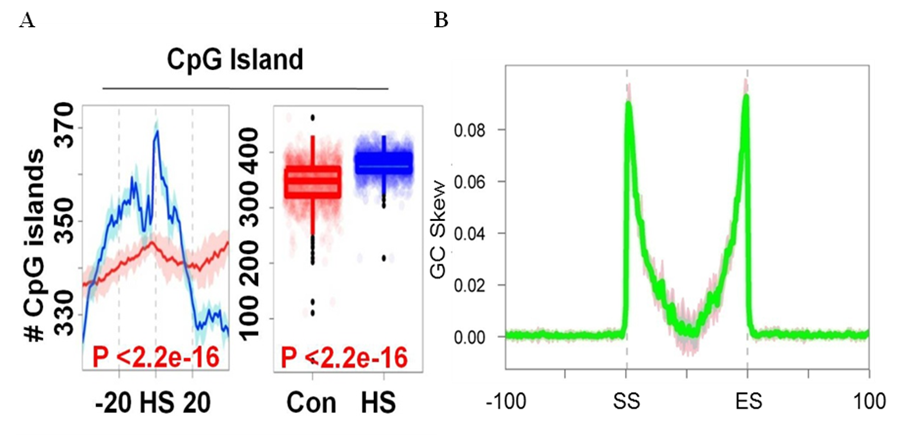


**Supplementary Figure 3**. CpG islands distributed around hotspot regions and GC skew distributed around gene content. **(A)** Distribution of CpG islands around hotspot regions in segregating populations. “HS” is the short name of hotspot, while “Con” shows the control random samples. “-20” and “20” represent 20 Mb upstream and downstream genomic regions of hotspots. Blue and red represent recombination hotspots and control random samples, respectively. **(B)** GC composition skew values in 1Kb bins spanning the -100Kb of upstream and +100Kb of downstream of gene content. GC skew value was calculated as (G-C)/(G+C). SS and ES indicate the start and end of transcription site, respectively.


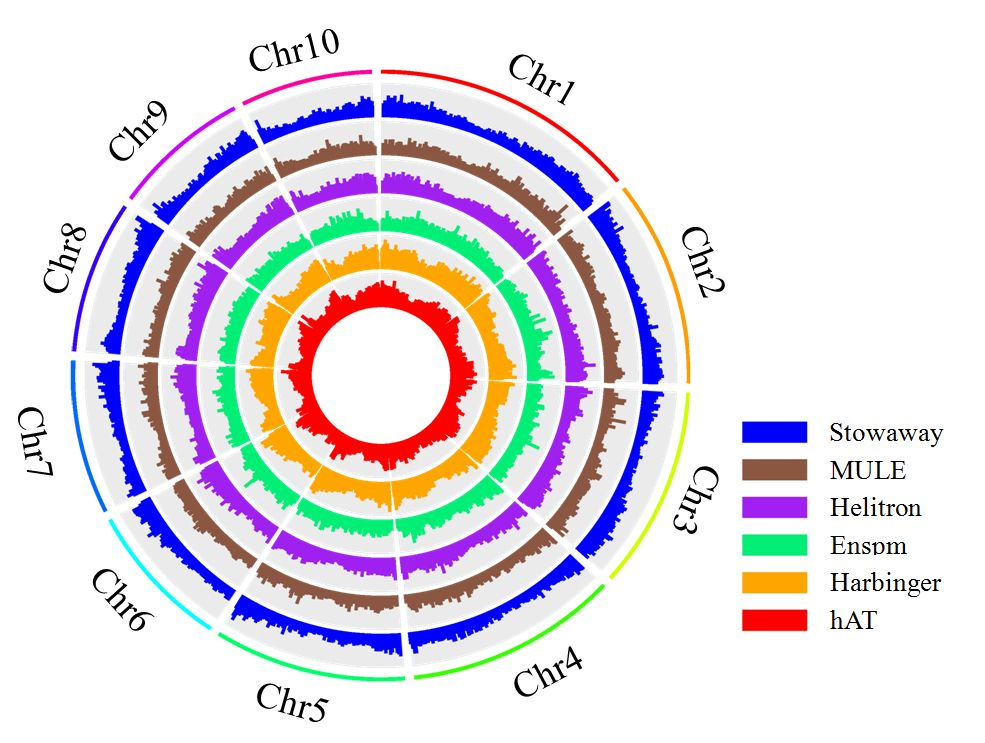


**Supplementary Figure 4**. Genome wide distribution of six types of DNA transposons. The histogram plot along each chromosome indicates the number of DNA transposons in 1Mb sliding bin.


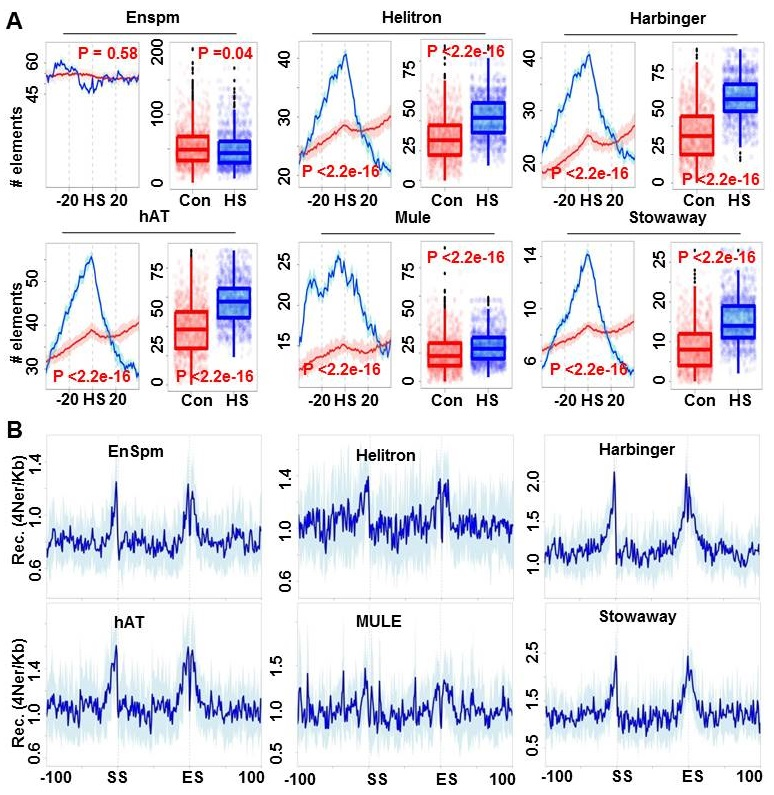


**Supplementary Figure 5**. Distribution of six DNA transposons around hotspot regions in segregating and natural populations. **(A)** Distribution of six DNA transposons around hotspot regions in segregating populations. “HS” is the short name of hotspot, while “Con” shows the control random samples. “-20” and “20” represent 20 Mb upstream and downstream genomic regions of hotspots. Blue and red represent recombination hotspots and control random samples, respectively. **(B)** Distribution of six DNA transposons around hotspot regions in natural populations. SS and ES are the start and end transcription site, respectively.

**
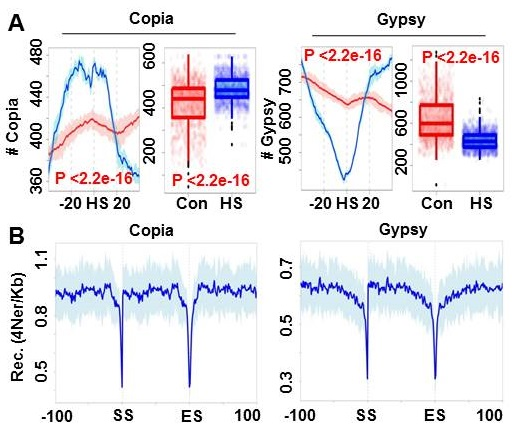
**

**Supplementary Figure 6**. Distribution of Copia and Gypsy retrotransposons around hotspot regions in segregating and natural populations. **(A)** Distribution of Copia and Gypsy retrotransposons around hotspot regions in segregating populations. “HS” is the short name of hotspot, while “Con” shows the control random samples. “-20” and “20” represent 20 Mb upstream and downstream genomic regions of hotspots. Blue and red represent recombination hotspots and control random samples, respectively. **(B)** Distribution of Copia and Gypsy retrotransposons around hotspot regions in natural populations. SS and ES are the start and end transcription site, respectively.

**
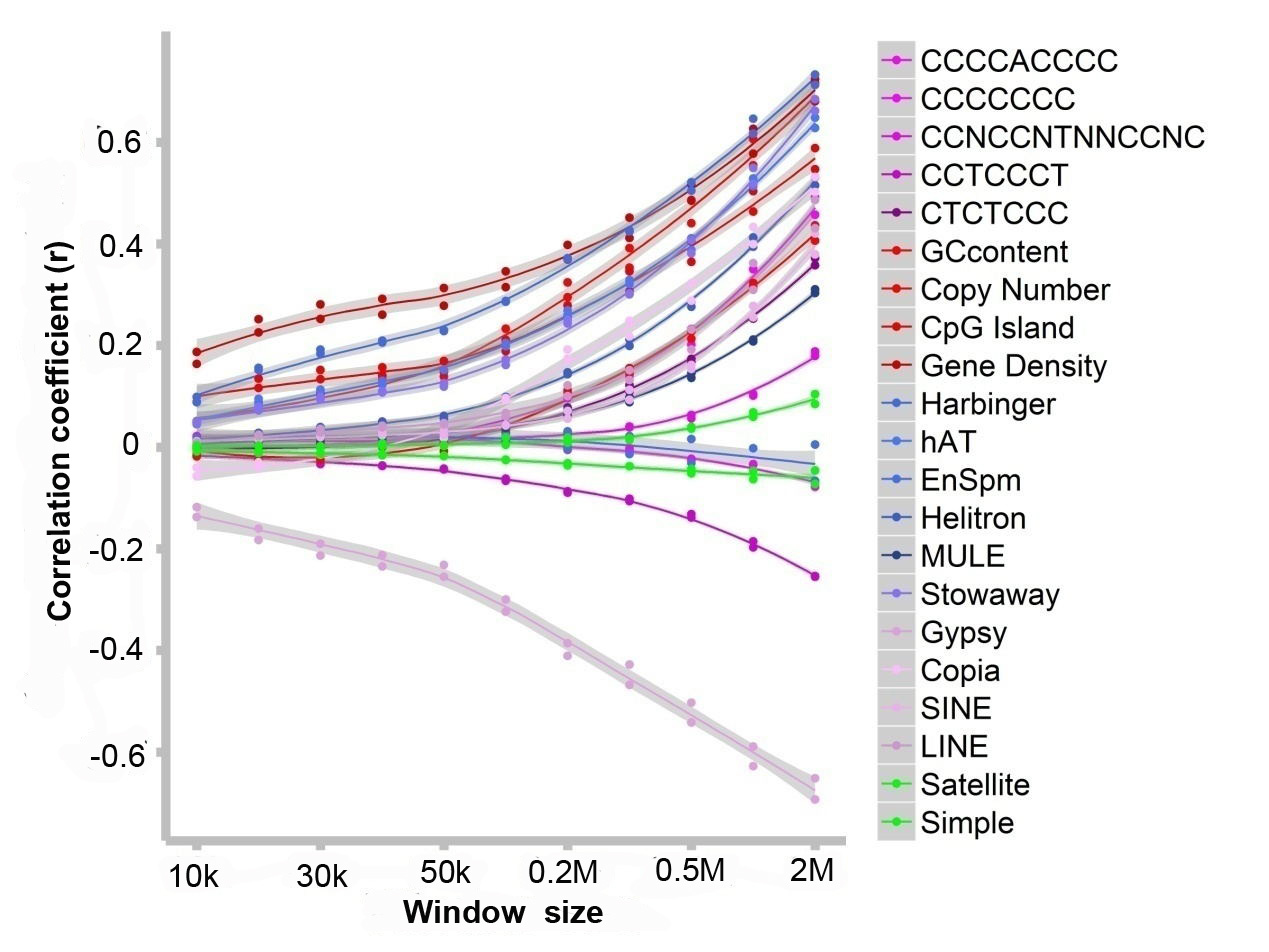
**

**Supplementary Figure 7**. Correlations over 11 sliding window sizes between 21 different genome contents and recombination rate in temperate and tropical subpopulations of natural populations

**Supplementary Table 1.** Summary of recombination QTL mapping in 11 RIL populations

| Trait^a^ | Population | Chr | Peak_gen (cM) | LOD | Additive | *R2* (%) | Peak_bin (Mb) | Left_bin (Mb) | Right_bin (Mb) | QTL_type^b^ |
| --- | --- | --- | --- | --- | --- | --- | --- | --- | --- | --- |
| chr1 | B73/BY804 | 2 | 21.1 | 3.57 | -0.85 | 6.93 | 3.8-3.9 | 3.4-3.5 | 4.2-4.2 | *Trans* |
| chr1 | K22/BY815 | 2 | 35.9 | 2.61 | 1 | 4.8 | 11.4-11.4 | 11.1-11.2 | 11.7-11.7 | *Trans* |
| chr1 | K22/BY815 | 5 | 87.7 | 5.09 | 1.75 | 9.93 | 164.7-164.7 | 160.7-161.2 | 167.7-167.9 | *Trans* |
| chr1 | KUI3/B77 | 10 | 15.6 | 3.03 | -0.85 | 6.66 | 4-4.1 | 2.5-2.5 | 4.7-4.7 | *Trans* |
| chr1 | K22/CI7 | 1 | 95.8 | 5.17 | 1.36 | 10.35 | 53.4-54.6 | 53.2-53.2 | 56.3-56.3 | *Cis* |
| chr1 | K22/CI7 | 1 | 104.6 | 7.86 | 1.84 | 15.57 | 67-69.1 | 65.9-66.6 | 74.2-74.4 | *Cis* |
| chr1 | K22/CI7 | 5 | 171.8 | 2.81 | -0.83 | 5.22 | 214.4-214.4 | 213.5-213.7 | 215.1-215.1 | *Trans* |
| chr1 | K22/CI7 | 6 | 57.5 | 4.63 | 1.06 | 8.57 | 111.9-112.2 | 109.3-109.9 | 112.7-113.3 | *Trans* |
| chr1 | DAN340/K22 | 5 | 163.2 | 2.77 | 0.95 | 6.11 | 210.5-210.5 | 209-209 | 211.4-211.4 | *Trans* |
| chr1 | KUI3/SC55 | 3 | 166.9 | 5.03 | -1.53 | 10.67 | 213.8-213.8 | 213-213.4 | 213.8-213.8 | *Trans* |
| chr1 | ZHENG58/SK | 5 | 158.3 | 4.04 | -1.52 | 7.64 | 205.6-205.7 | 205.5-205.6 | 205.8-206 | *Trans* |
| chr1 | ZHENG58/SK | 6 | 34.8 | 2.66 | 0.95 | 4.98 | 100.1-100.1 | 98.6-98.8 | 102.5-104.1 | *Trans* |
| chr1 | YU87-1/BK | 3 | 137.9 | 2.86 | -1.16 | 5.23 | 196.7-197.3 | 195.3-195.7 | 201.3-201.5 | *Trans* |
| chr1 | YU87-1/BK | 8 | 80.2 | 3.18 | -1.38 | 7.45 | 123.5-124.4 | 122.9-122.9 | 130.4-131.2 | *Trans* |
| chr1 | ZONG3/YU87-1 | 3 | 3.3 | 2.8 | -1.18 | 5.34 | 1.5-1.5 | 1.3-1.3 | 1.7-1.8 | *Trans* |
| chr10 | B73/BY804 | 4 | 10.4 | 2.96 | -0.48 | 5.73 | 2.9-2.9 | 1.5-1.5 | 3.1-3.1 | *Trans* |
| chr10 | B73/BY804 | 5 | 73.4 | 2.81 | 0.45 | 5.46 | 20.8-21 | 19.8-20.3 | 23-23.5 | *Trans* |
| chr10 | DE3/BY815 | 8 | 89.9 | 2.8 | 0.42 | 4.78 | 129.4-131.2 | 121.9-121.9 | 131.4-131.5 | *Trans* |
| chr10 | K22/BY815 | 10 | 105.3 | 3.4 | 0.79 | 6.94 | 144.8-144.8 | 144.5-144.7 | 145.4-145.9 | *Cis* |
| chr10 | KUI3/B77 | 2 | 172.5 | 2.63 | 0.39 | 5.92 | 223.3-224.1 | 222.9-222.9 | 225.5-225.5 | *Trans* |
| chr10 | KUI3/B77 | 10 | 47.9 | 3.73 | -0.61 | 8.3 | 14.1-14.1 | 11.9-13.3 | 14.8-15 | *Cis* |
| chr10 | BY815/KUI3 | 3 | 33.1 | 3.02 | 0.51 | 6.14 | 9-9.1 | 7.6-7.6 | 9.5-9.5 | *Trans* |
| chr10 | BY815/KUI3 | 7 | 75.6 | 3.46 | 0.68 | 6.76 | 122.1-122.1 | 121-121.2 | 123.7-123.7 | *Trans* |
| chr10 | K22/CI7 | 1 | 204.5 | 3.23 | -0.42 | 6.27 | 262.2-262.5 | 257.4-258 | 262.9-262.9 | *Trans* |
| chr10 | K22/CI7 | 1 | 213.5 | 3.24 | -0.43 | 6.4 | 271.8-271.8 | 270.7-271.2 | 274.4-274.4 | *Trans* |
| chr10 | K22/CI7 | 8 | 134.5 | 2.63 | 0.48 | 5.13 | 168.3-168.5 | 168.2-168.2 | 170.1-170.1 | *Trans* |
| chr10 | K22/CI7 | 8 | 152.6 | 2.62 | -0.41 | 5.46 | 173-173.1 | 172.8-172.8 | 174-174 | *Trans* |
| chr10 | DAN340/K22 | 6 | 60.2 | 3.19 | -0.52 | 5.26 | 112.7-112.8 | 109-109.1 | 115.4-115.6 | *Trans* |
| chr10 | DAN340/K22 | 10 | 19 | 2.64 | -0.53 | 5.1 | 4.9-4.9 | 4.1-4.1 | 5.7-5.8 | *Cis* |
| chr10 | DAN340/K22 | 10 | 27.9 | 3.83 | -0.66 | 6.59 | 6.1-6.1 | 6.1-6.1 | 11.8-11.9 | *Cis* |
| chr10 | DAN340/K22 | 10 | 41.4 | 8 | 1.18 | 12.72 | 42.1-42.5 | 25.3-27.2 | 65-73.7 | *Cis* |
| chr10 | DAN340/K22 | 10 | 48.9 | 8.61 | 1.03 | 16.58 | 86.8-87 | 85.6-86.5 | 99.2-99.2 | *Cis* |
| chr10 | KUI3/SC55 | 3 | 57.2 | 4.1 | 0.52 | 8.23 | 16.6-16.6 | 13.3-13.5 | 17-17.4 | *Trans* |
| chr10 | KUI3/SC55 | 10 | 48.3 | 3 | 0.44 | 6.14 | 10.4-10.4 | 8.9-9.5 | 11.7-11.7 | *Cis* |
| chr10 | ZHENG58/SK | 3 | 32.5 | 3.53 | 0.49 | 6.6 | 6.1-6.4 | 5.9-5.9 | 6.9-6.9 | *Trans* |
| chr10 | YU87-1/BK | 1 | 30 | 5.37 | 0.62 | 12 | 7.1-7.3 | 7月7日 | 7.7-7.9 | *Trans* |
| chr10 | YU87-1/BK | 1 | 39.8 | 2.74 | 0.46 | 6.62 | 10.1-10.8 | 9.4-10 | 14.9-15.2 | *Trans* |
| chr10 | YU87-1/BK | 4 | 108.1 | 3.02 | 0.46 | 6.7 | 169.8-170 | 167.2-169 | 171.3-171.5 | *Trans* |
| chr10 | ZONG3/YU87-1 | 3 | 285.5 | 3.35 | -0.6 | 6.12 | 230.4-230.4 | 229.4-229.6 | 232-232 | *Trans* |
| chr10 | ZONG3/YU87-1 | 6 | 19.2 | 2.66 | -0.58 | 5.61 | 8.7-8.7 | 7月7日 | 16.1-24 | *Trans* |
| chr10 | ZONG3/YU87-1 | 8 | 50.6 | 3.06 | 0.59 | 5.53 | 9.9-9.9 | 8.8-8.8 | 10月10日 | *Trans* |
| chr2 | B73/BY804 | 4 | 62.7 | 3.96 | 0.85 | 7.49 | 23.8-25.5 | 22.8-22.8 | 26.6-27.5 | *Trans* |
| chr2 | B73/BY804 | 5 | 148.1 | 5.74 | 1.08 | 10.82 | 204.8-204.8 | 203.8-204.1 | 205.3-205.4 | *Trans* |
| chr2 | B73/BY804 | 5 | 154.6 | 5.04 | 1.04 | 9.7 | 206.9-206.9 | 206.6-206.6 | 208.1-208.2 | *Trans* |
| chr2 | K22/BY815 | 6 | 22.9 | 2.93 | 0.85 | 5.91 | 57.5-75.4 | 34.5-34.6 | 86.3-86.3 | *Trans* |
| chr2 | KUI3/B77 | 2 | 101.2 | 3.39 | -0.99 | 8.35 | 170.2-172.5 | 156.8-157.6 | 170.2-172.5 | *Cis* |
| chr2 | KUI3/B77 | 2 | 107.9 | 3.15 | -0.71 | 6.89 | 182-182 | 180.7-180.7 | 182.2-182.2 | *Cis* |
| chr2 | KUI3/B77 | 5 | 10.4 | 2.74 | -0.64 | 5.59 | 2.3-2.3 | 2.1-2.1 | 2.9-3.2 | *Trans* |
| chr2 | BY815/KUI3 | 1 | 180.6 | 3.24 | 0.96 | 6.43 | 211.3-211.3 | 209-209 | 213-213 | *Trans* |
| chr2 | K22/CI7 | 2 | 83.6 | 7.16 | 2.21 | 18.56 | 44.3-44.3 | 43.4-44.3 | 47.4-47.4 | *Cis* |
| chr2 | K22/CI7 | 2 | 88.6 | 3.28 | 1.04 | 7.69 | 59.7-60.8 | 55-55.6 | 62.5-63.8 | *Cis* |
| chr2 | K22/CI7 | 2 | 100 | 5.28 | -1.88 | 12.49 | 157.6-157.6 | 153-154.2 | 164.2-164.2 | *Cis* |
| chr2 | K22/CI7 | 7 | 0 | 3.01 | 0.8 | 4.55 | 0.8-1 | 0.8-1 | 1.1-1.2 | *Trans* |
| chr2 | K22/CI7 | 7 | 5.8 | 3.19 | 0.92 | 5.62 | 2.1-2.1 | 1.3-1.3 | 2.6-2.6 | *Trans* |
| chr2 | DAN340/K22 | 5 | 178 | 2.81 | 0.61 | 5.43 | 213.3-213.3 | 212.7-212.8 | 213.5-213.5 | *Trans* |
| chr2 | KUI3/SC55 | 1 | 100.8 | 2.7 | -0.7 | 5.39 | 69.9-70.8 | 67-68.1 | 71.6-72 | *Trans* |
| chr2 | KUI3/SC55 | 1 | 170.7 | 3.77 | 0.85 | 7.77 | 208.3-208.8 | 207.1-207.2 | 211-211.3 | *Trans* |
| chr2 | KUI3/SC55 | 7 | 12.3 | 2.95 | -0.76 | 6.25 | 2.1-2.1 | 2月2日 | 2.5-2.5 | *Trans* |
| chr2 | ZHENG58/SK | 4 | 67.6 | 3.55 | 0.86 | 6.62 | 29.8-30.8 | 26.4-26.6 | 31.8-32.2 | *Trans* |
| chr2 | ZHENG58/SK | 8 | 77.7 | 2.67 | -0.73 | 4.88 | 118.1-118.1 | 112.3-112.4 | 119-119.7 | *Trans* |
| chr2 | ZHENG58/SK | 10 | 86.6 | 2.93 | 0.83 | 5.3 | 143.5-143.7 | 142.9-142.9 | 144.1-144.1 | *Trans* |
| chr2 | YU87-1/BK | 7 | 5.9 | 2.63 | -0.76 | 5.86 | 2.1-2.1 | 1.3-1.3 | 2.6-2.6 | *Trans* |
| chr2 | ZONG3/YU87-1 | 2 | 110.8 | 3.36 | -0.95 | 5.75 | 36.8-36.8 | 33.5-33.5 | 38.6-38.7 | *Cis* |
| chr2 | ZONG3/YU87-1 | 2 | 121.2 | 3.19 | -0.94 | 5.61 | 44.3-44.3 | 43.4-43.4 | 44.4-46 | *Cis* |
| chr2 | ZONG3/YU87-1 | 2 | 262 | 3.36 | -1.01 | 6.39 | 216.3-216.6 | 215.3-215.5 | 217.4-217.6 | *Cis* |
| chr2 | ZONG3/YU87-1 | 3 | 2.3 | 4.39 | -1.09 | 7.6 | 1.5-1.5 | 1.3-1.3 | 1.7-1.8 | *Trans* |
| chr2 | ZONG3/YU87-1 | 7 | 225.5 | 2.65 | -0.83 | 4.5 | 168.1-168.1 | 166.1-166.5 | 168.4-168.6 | *Trans* |
| chr2 | ZONG3/YU87-1 | 7 | 239 | 3.11 | 1.09 | 4.93 | 171.8-171.8 | 171.7-171.7 | 172.1-172.2 | *Trans* |
| chr2 | ZONG3/YU87-1 | 9 | 0 | 3.72 | -1 | 6.47 | 0.1-0.4 | 0.1-0.4 | 1.3-1.3 | *Trans* |
| chr2 | ZONG3/YU87-1 | 9 | 9.1 | 4.32 | -1.07 | 7.47 | 2.3-2.3 | 2.1-2.1 | 2.5-2.5 | *Trans* |
| chr2 | ZONG3/YU87-1 | 9 | 14.3 | 3.27 | -0.93 | 5.7 | 3.9-4.3 | 3.3-3.3 | 4.7-4.7 | *Trans* |
| chr3 | DE3/BY815 | 1 | 41.8 | 3.35 | -0.62 | 6.2 | 12.5-12.5 | 12.2-12.2 | 13-13 | *Trans* |
| chr3 | DE3/BY815 | 1 | 62.5 | 3.04 | 0.68 | 5.51 | 23.2-23.2 | 22.3-22.4 | 24.2-24.2 | *Trans* |
| chr3 | DE3/BY815 | 1 | 69.5 | 4.29 | 0.69 | 7.4 | 30.3-30.3 | 27.2-27.6 | 33.3-33.5 | *Trans* |
| chr3 | DE3/BY815 | 7 | 91.3 | 3.28 | -0.56 | 5.53 | 145.2-145.2 | 140.7-141.5 | 145.7-145.8 | *Trans* |
| chr3 | DE3/BY815 | 7 | 137.6 | 4.02 | 0.64 | 7.01 | 168.1-168.1 | 166.9-166.9 | 168.2-168.2 | *Trans* |
| chr3 | K22/BY815 | 8 | 128.1 | 2.7 | -0.6 | 5.37 | 169-169 | 168-168 | 169.1-169.1 | *Trans* |
| chr3 | K22/BY815 | 8 | 137.4 | 4.55 | -0.77 | 9.12 | 170.8-170.9 | 170.1-170.1 | 171.2-171.2 | *Trans* |
| chr3 | KUI3/B77 | 1 | 114.6 | 2.78 | 0.51 | 5.92 | 86.6-86.6 | 79.4-80.2 | 88.6-89.3 | *Trans* |
| chr3 | KUI3/B77 | 1 | 185.9 | 3.4 | -0.58 | 7.49 | 215.3-216.2 | 214.3-214.9 | 216.5-216.5 | *Trans* |
| chr3 | KUI3/B77 | 1 | 191.7 | 4.3 | -0.65 | 9.36 | 223-223.3 | 220.4-220.6 | 223.9-223.9 | *Trans* |
| chr3 | KUI3/B77 | 4 | 132.2 | 2.73 | 0.52 | 5.77 | 216.2-218.1 | 206.1-210.7 | 228-228.6 | *Trans* |
| chr3 | BY815/KUI3 | 1 | 14.8 | 4.65 | 0.96 | 8.43 | 6.2-6.2 | 6月6日 | 6.2-6.2 | *Trans* |
| chr3 | BY815/KUI3 | 1 | 44.1 | 3.52 | -0.83 | 6.39 | 17.5-17.5 | 16.4-16.4 | 17.8-18 | *Trans* |
| chr3 | BY815/KUI3 | 3 | 205.4 | 3.93 | 0.81 | 7.1 | 228.5-229 | 227.6-227.6 | 229.4-229.4 | *Cis* |
| chr3 | BY815/KUI3 | 5 | 124.3 | 2.62 | 0.88 | 4.47 | 182.1-182.2 | 181.6-181.6 | 182.4-182.5 | *Trans* |
| chr3 | BY815/KUI3 | 8 | 118 | 3.28 | -0.75 | 5.96 | 162.6-163.2 | 161.1-161.1 | 163.7-163.7 | *Trans* |
| chr3 | K22/CI7 | 5 | 93.6 | 2.82 | -0.57 | 5.89 | 167.9-168.1 | 164.7-166 | 170.5-170.5 | *Trans* |
| chr3 | DAN340/K22 | 3 | 117 | 3.54 | -0.88 | 6.68 | 203.2-204.2 | 201.6-201.6 | 205-205.1 | *Cis* |
| chr3 | KUI3/SC55 | 2 | 70.5 | 2.79 | 0.59 | 5.65 | 27.6-28 | 23.9-24.7 | 28.7-28.7 | *Trans* |
| chr3 | ZHENG58/SK | 2 | 191.4 | 4.25 | 0.89 | 7.9 | 232.8-232.8 | 231.3-231.9 | 233-233 | *Trans* |
| chr3 | ZHENG58/SK | 4 | 102.4 | 2.86 | -0.64 | 5.48 | 160.3-160.6 | 157.6-157.6 | 161.1-161.1 | *Trans* |
| chr3 | ZONG3/YU87-1 | 3 | 223.7 | 4.81 | -0.98 | 9.19 | 207.5-207.5 | 207-207.2 | 208-208.6 | *Cis* |
| chr3 | ZONG3/YU87-1 | 7 | 81.3 | 2.83 | -1.03 | 5.65 | 28.4-29.2 | 26.2-27.5 | 29.3-29.3 | *Trans* |
| chr3 | ZONG3/YU87-1 | 7 | 92.1 | 7.34 | -2.01 | 14.25 | 107-107.2 | 101.9-106.6 | 107.5-107.5 | *Trans* |
| chr4 | B73/BY804 | 5 | 90.4 | 3.1 | -1 | 6.29 | 69.3-69.3 | 68.5-69.3 | 76.4-78.4 | *Trans* |
| chr4 | B73/BY804 | 7 | 84.4 | 2.78 | -0.74 | 5.57 | 131-131 | 129.9-129.9 | 132.8-133.9 | *Trans* |
| chr4 | B73/BY804 | 7 | 91.8 | 4.48 | -1 | 8.57 | 141.9-141.9 | 141.1-141.5 | 143.3-143.3 | *Trans* |
| chr4 | DE3/BY815 | 1 | 207.6 | 2.77 | -0.63 | 4.51 | 274.9-275 | 272.8-273.1 | 275-275.9 | *Trans* |
| chr4 | DE3/BY815 | 4 | 54.5 | 5.31 | -0.98 | 9.8 | 17.5-17.5 | 16.8-17.2 | 18.2-20.2 | *Cis* |
| chr4 | DE3/BY815 | 4 | 86.9 | 3.1 | 0.73 | 5.26 | 157.5-157.5 | 156.5-156.5 | 157.9-157.9 | *Cis* |
| chr4 | DE3/BY815 | 8 | 143.2 | 2.94 | -0.66 | 4.84 | 169.7-169.8 | 169.2-169.2 | 170.1-170.1 | *Trans* |
| chr4 | K22/BY815 | 1 | 127.7 | 3.88 | 0.92 | 7.31 | 184.6-184.6 | 182.9-183.8 | 191-191.1 | *Trans* |
| chr4 | KUI3/B77 | 4 | 49 | 2.71 | 0.59 | 5.39 | 19.4-19.6 | 17.5-17.5 | 21.7-21.7 | *Cis* |
| chr4 | KUI3/B77 | 5 | 23.3 | 3.25 | 0.66 | 6.67 | 4.8-4.8 | 3.9-3.9 | 5.2-5.2 | *Trans* |
| chr4 | KUI3/B77 | 7 | 12.1 | 2.62 | 0.6 | 5.58 | 2.6-2.6 | 2-2.1 | 3.7-3.7 | *Trans* |
| chr4 | BY815/KUI3 | 4 | 81 | 3.55 | 0.97 | 7.58 | 74.6-75.7 | 73.4-74.4 | 79.5-80.5 | *Cis* |
| chr4 | BY815/KUI3 | 4 | 90.6 | 5.54 | 1.15 | 10.62 | 151.7-151.9 | 147.6-150 | 152.2-152.2 | *Cis* |
| chr4 | BY815/KUI3 | 4 | 104.4 | 6.1 | -1.24 | 12.55 | 158.1-158.1 | 157.9-157.9 | 161.6-162.3 | *Cis* |
| chr4 | BY815/KUI3 | 4 | 111.7 | 3.91 | -0.99 | 8.74 | 166-166 | 164.1-164.2 | 167-167 | *Cis* |
| chr4 | K22/CI7 | 4 | 7.9 | 3.32 | -0.89 | 6.98 | 2.8-2.8 | 2.1-2.1 | 2.9-2.9 | *Cis* |
| chr4 | K22/CI7 | 4 | 19.5 | 4.88 | 1.05 | 8.88 | 5.7-5.8 | 4.9-4.9 | 6月6日 | *Cis* |
| chr4 | DAN340/K22 | 4 | 97.7 | 4.28 | 0.68 | 8.37 | 186.6-186.9 | 185.6-185.6 | 187.9-187.9 | *Cis* |
| chr4 | DAN340/K22 | 4 | 103.8 | 4.35 | 0.68 | 8.26 | 203.1-203.8 | 202.3-202.3 | 205.2-206 | *Cis* |
| chr4 | DAN340/K22 | 4 | 111.6 | 3.07 | 0.65 | 5.94 | 229.3-229.3 | 229-229.3 | 229.8-230.3 | *Cis* |
| chr4 | DAN340/K22 | 6 | 41.2 | 2.9 | -0.49 | 5.48 | 96.9-96.9 | 95.9-96.2 | 97.3-97.6 | *Trans* |
| chr4 | DAN340/K22 | 7 | 4.1 | 3.49 | -0.52 | 6.62 | 1.2-1.2 | 0.8-0.8 | 1.3-1.3 | *Trans* |
| chr4 | KUI3/SC55 | 6 | 151.9 | 2.6 | -0.58 | 5.04 | 162.2-162.4 | 161.4-161.7 | 162.9-162.9 | *Trans* |
| chr4 | KUI3/SC55 | 7 | 17.6 | 3.66 | -0.68 | 7.18 | 2.5-2.5 | 2.1-2.1 | 3月3日 | *Trans* |
| chr4 | KUI3/SC55 | 10 | 80.4 | 2.63 | -0.58 | 5.09 | 114.2-115.6 | 100-105.3 | 118.5-119.8 | *Trans* |
| chr4 | ZHENG58/SK | 4 | 46.2 | 4.35 | 0.69 | 7.66 | 13.8-14 | 11.9-12.1 | 14.4-14.4 | *Cis* |
| chr4 | ZHENG58/SK | 9 | 3 | 4.12 | 0.68 | 7.45 | 1.5-2.1 | 1月1日 | 2.3-2.3 | *Trans* |
| chr4 | YU87-1/BK | 5 | 217.3 | 2.84 | 0.69 | 5.98 | 215.6-215.6 | 215-215 | 215.9-215.9 | *Trans* |
| chr4 | ZONG3/YU87-1 | 1 | 72.1 | 3.57 | -0.81 | 6.86 | 26.7-26.7 | 25.6-25.9 | 27.2-27.6 | *Trans* |
| chr5 | B73/BY804 | 7 | 142.1 | 3.14 | 0.66 | 5.89 | 168.4-168.4 | 168.1-168.1 | 169.1-169.3 | *Trans* |
| chr5 | DE3/BY815 | 5 | 45.8 | 2.79 | -0.92 | 3.53 | 10.1-10.1 | 10月10日 | 11.7-11.7 | *Cis* |
| chr5 | DE3/BY815 | 5 | 54.2 | 3.37 | -1.05 | 4.06 | 12.6-12.6 | 12.4-12.5 | 13.1-13.1 | *Cis* |
| chr5 | DE3/BY815 | 5 | 72.2 | 6.22 | 1.93 | 8.47 | 22.8-22.8 | 19.7-19.7 | 22.8-22.8 | *Cis* |
| chr5 | DE3/BY815 | 5 | 80.1 | 8.53 | -2.82 | 17.6 | 36.5-39.5 | 35.7-35.7 | 40.8-42.7 | *Cis* |
| chr5 | DE3/BY815 | 5 | 94.3 | 8.11 | 2.59 | 14.66 | 67.5-68.4 | 66-67.1 | 76.4-76.6 | *Cis* |
| chr5 | K22/BY815 | 1 | 163.5 | 2.82 | 0.78 | 3.88 | 230.2-230.2 | 228.6-229.1 | 232.7-233.3 | *Trans* |
| chr5 | K22/BY815 | 5 | 83 | 3.9 | -1.01 | 6.06 | 144.7-144.8 | 139.5-139.5 | 147.7-150.4 | *Cis* |
| chr5 | K22/BY815 | 5 | 94.3 | 5.39 | -1.24 | 7.64 | 170.1-171.2 | 169.7-169.7 | 172-172 | *Cis* |
| chr5 | K22/BY815 | 5 | 120 | 11.4 | 2.38 | 18.56 | 204.2-204.6 | 204.2-204.2 | 204.8-204.8 | *Cis* |
| chr5 | K22/BY815 | 5 | 132.5 | 11.2 | -2.17 | 17.66 | 208.4-208.4 | 208.1-208.1 | 210-210 | *Cis* |
| chr5 | BY815/KUI3 | 4 | 135.3 | 2.81 | 0.71 | 5.54 | 184.9-184.9 | 182.4-183.1 | 184.9-185 | *Trans* |
| chr5 | KUI3/SC55 | 6 | 121.8 | 2.96 | 0.59 | 5.51 | 152.2-152.2 | 151.2-151.5 | 152.5-152.5 | *Trans* |
| chr5 | KUI3/SC55 | 9 | 71.9 | 2.83 | 0.58 | 5.42 | 115-115.6 | 112.1-113.7 | 116.9-117.1 | *Trans* |
| chr5 | KUI3/SC55 | 10 | 0 | 3.76 | -0.69 | 7.73 | 0.6-1.7 | 0.6-1.7 | 1.9-1.9 | *Trans* |
| chr5 | ZHENG58/SK | 9 | 97.3 | 3.53 | -0.75 | 7.06 | 146.5-146.5 | 143.2-144.8 | 146.7-146.7 | *Trans* |
| chr5 | YU87-1/BK | 5 | 82.5 | 3.63 | -1.36 | 7.84 | 26.7-27.8 | 20.5-20.5 | 31.6-31.9 | *Cis* |
| chr5 | YU87-1/BK | 5 | 89.5 | 5.16 | -1.71 | 10.87 | 49.8-49.9 | 35.6-36.5 | 55-55 | *Cis* |
| chr5 | YU87-1/BK | 5 | 94.8 | 4.08 | 1.35 | 10.69 | 59.5-62.2 | 56.4-57.2 | 62.7-62.7 | *Cis* |
| chr5 | YU87-1/BK | 5 | 100.4 | 5.95 | 2.7 | 14.9 | 80.8-80.8 | 74.6-78.6 | 126.8-126.8 | *Cis* |
| chr5 | ZONG3/YU87-1 | 4 | 76.8 | 2.72 | -0.86 | 4.9 | 21.7-21.7 | 19.1-19.1 | 22.8-26.1 | *Trans* |
| chr5 | ZONG3/YU87-1 | 7 | 258.3 | 3.46 | 0.97 | 6.29 | 174.5-175 | 173.8-173.8 | 175.3-175.3 | *Trans* |
| chr6 | B73/BY804 | 1 | 142.7 | 5.31 | 0.68 | 9.92 | 210.7-210.7 | 208.2-208.3 | 211.5-212.6 | *Trans* |
| chr6 | B73/BY804 | 1 | 147.9 | 5.45 | 0.69 | 10.16 | 215.8-216.7 | 213.2-214.9 | 217.5-217.5 | *Trans* |
| chr6 | DE3/BY815 | 1 | 37.2 | 2.63 | -0.68 | 4.37 | 12-12.1 | 11-11.4 | 12.2-12.2 | *Trans* |
| chr6 | DE3/BY815 | 3 | 144.7 | 3.2 | -0.75 | 5.27 | 217.4-217.4 | 217.1-217.2 | 217.7-217.8 | *Trans* |
| chr6 | DE3/BY815 | 6 | 1.2 | 3.71 | -0.97 | 6.78 | 2.4-2.7 | 0.3-0.7 | 4月4日 | *Cis* |
| chr6 | DE3/BY815 | 6 | 9.7 | 6.36 | -1.34 | 10.87 | 8月8日 | 7月7日 | 8.1-8.7 | *Cis* |
| chr6 | DE3/BY815 | 6 | 15.7 | 4.85 | -1.5 | 9.17 | 14.5-14.5 | 9.1-9.5 | 18.2-18.8 | *Cis* |
| chr6 | DE3/BY815 | 6 | 27.9 | 7.16 | 1.42 | 12.66 | 59.9-66 | 36.4-39.3 | 67.3-76.7 | *Cis* |
| chr6 | K22/BY815 | 6 | 13.2 | 2.86 | -0.96 | 5.68 | 14.4-14.4 | 9.2-9.5 | 16.2-24.6 | *Cis* |
| chr6 | K22/BY815 | 6 | 26 | 7.04 | 1.29 | 12.88 | 76.2-78.6 | 57.5-75.4 | 80-81.4 | *Cis* |
| chr6 | K22/BY815 | 6 | 34.9 | 4.91 | -1.24 | 10.44 | 95.6-96.3 | 90.5-90.5 | 97.2-97.3 | *Cis* |
| chr6 | KUI3/B77 | 9 | 43.5 | 2.95 | -0.55 | 5.62 | 20.8-20.8 | 19.9-20.3 | 20.9-20.9 | *Trans* |
| chr6 | KUI3/B77 | 10 | 94.5 | 3.47 | -0.68 | 7.73 | 140-140 | 139.8-139.8 | 140-140 | *Trans* |
| chr6 | BY815/KUI3 | 5 | 206.9 | 2.65 | -0.57 | 4.82 | 213.9-214.1 | 213.2-213.2 | 214.1-214.1 | *Trans* |
| chr6 | BY815/KUI3 | 6 | 61.9 | 2.71 | 0.59 | 5.11 | 124.8-124.8 | 115.1-115.6 | 128.1-128.7 | *Cis* |
| chr6 | K22/CI7 | 7 | 83.6 | 3.32 | -0.52 | 6.89 | 133.6-133.6 | 132.7-132.8 | 137.7-137.7 | *Trans* |
| chr6 | DAN340/K22 | 6 | 91.4 | 3.95 | -0.92 | 8.21 | 148.2-148.2 | 147.7-147.9 | 148.5-148.8 | *Cis* |
| chr6 | DAN340/K22 | 6 | 104 | 7.67 | 1.49 | 17.37 | 152.2-152.2 | 151.6-151.6 | 153.2-153.2 | *Cis* |
| chr6 | DAN340/K22 | 6 | 112.7 | 2.83 | 0.8 | 5.97 | 154.9-154.9 | 154.6-154.6 | 155.6-155.6 | *Cis* |
| chr6 | ZHENG58/SK | 1 | 251 | 5.26 | -0.81 | 9.93 | 281-281.1 | 280.2-280.2 | 281.2-281.3 | *Trans* |
| chr6 | YU87-1/BK | 9 | 83.5 | 3.31 | -0.59 | 6.79 | 133.7-133.7 | 130.1-132 | 134.1-134.3 | *Trans* |
| chr6 | ZONG3/YU87-1 | 4 | 243.6 | 3.59 | 0.71 | 6.69 | 237.5-237.5 | 237-237 | 237.5-237.5 | *Trans* |
| chr6 | ZONG3/YU87-1 | 4 | 253.3 | 4.48 | 0.79 | 8.26 | 239-239 | 238.5-238.5 | 239.1-239.1 | *Trans* |
| chr6 | ZONG3/YU87-1 | 5 | 146.6 | 3.85 | 0.74 | 7.11 | 163.7-163.9 | 163.2-163.3 | 166.9-166.9 | *Trans* |
| chr6 | ZONG3/YU87-1 | 10 | 188.5 | 3.09 | 0.64 | 5.65 | 148.1-148.1 | 147.8-147.8 | 148.4-148.4 | *Trans* |
| chr7 | B73/BY804 | 7 | 129.4 | 2.74 | -0.76 | 6.53 | 165.5-165.5 | 165.2-165.2 | 165.8-165.9 | *Cis* |
| chr7 | B73/BY804 | 7 | 142.1 | 4.13 | 1 | 8.09 | 168.4-168.4 | 168.2-168.2 | 170.1-170.2 | *Cis* |
| chr7 | DE3/BY815 | 3 | 17 | 3.15 | -0.66 | 5.95 | 3.4-3.5 | 3.3-3.3 | 3.6-3.6 | *Trans* |
| chr7 | K22/BY815 | 1 | 105.1 | 2.93 | 0.76 | 5.43 | 82-82 | 72.8-74.2 | 91.2-91.5 | *Trans* |
| chr7 | K22/BY815 | 7 | 63.2 | 3.43 | 1.13 | 5.95 | 107-107 | 101.8-105.4 | 107.8-107.8 | *Cis* |
| chr7 | K22/BY815 | 7 | 128.6 | 4.06 | 0.91 | 7.54 | 171.2-171.2 | 170.7-170.8 | 172.7-172.7 | *Cis* |
| chr7 | K22/BY815 | 10 | 2 | 2.69 | -0.75 | 5.45 | 1.9-1.9 | 1.1-1.3 | 2.5-2.5 | *Trans* |
| chr7 | KUI3/B77 | 2 | 80.6 | 4.53 | -0.63 | 8.83 | 70.7-72.1 | 60.9-60.9 | 81.7-105 | *Trans* |
| chr7 | KUI3/B77 | 2 | 106.7 | 2.74 | 0.5 | 5.91 | 180.6-180.6 | 177.9-178.1 | 182-182 | *Trans* |
| chr7 | KUI3/B77 | 3 | 45.7 | 3.07 | 0.47 | 5.97 | 9.5-9.5 | 9月9日 | 10.7-11.4 | *Trans* |
| chr7 | KUI3/B77 | 3 | 51.2 | 3.4 | 0.49 | 6.7 | 13.3-13.3 | 12.2-12.2 | 18.2-18.2 | *Trans* |
| chr7 | K22/CI7 | 7 | 92.1 | 2.76 | -0.54 | 5.31 | 144.6-146 | 144-144.4 | 146.5-147.2 | *Cis* |
| chr7 | K22/CI7 | 7 | 100.4 | 2.92 | -0.49 | 5.62 | 153.3-153.6 | 152.8-152.9 | 153.8-153.8 | *Cis* |
| chr7 | K22/CI7 | 8 | 70.1 | 2.74 | -0.45 | 5.22 | 98.4-98.4 | 97-97 | 101.2-101.8 | *Trans* |
| chr7 | K22/CI7 | 8 | 80.2 | 3.14 | -0.48 | 5.94 | 121.9-122.4 | 116.9-118.2 | 124.6-125.2 | *Trans* |
| chr7 | DAN340/K22 | 7 | 49.1 | 3.31 | -0.6 | 6.54 | 15.3-15.5 | 14.9-14.9 | 27.5-27.5 | *Cis* |
| chr7 | KUI3/SC55 | 3 | 184.5 | 2.69 | 0.5 | 5.58 | 220.7-220.7 | 219.7-219.7 | 220.7-220.7 | *Trans* |
| chr7 | KUI3/SC55 | 3 | 191.7 | 4.11 | 0.63 | 8.37 | 222.7-222.7 | 221.7-221.9 | 223.1-223.3 | *Trans* |
| chr7 | ZHENG58/SK | 5 | 139.8 | 3.19 | 0.62 | 6.41 | 195.7-196.2 | 194-194 | 198.2-198.8 | *Trans* |
| chr7 | ZHENG58/SK | 5 | 146.7 | 3.56 | 0.65 | 6.84 | 202.4-202.8 | 201.9-202.1 | 204.5-204.6 | *Trans* |
| chr7 | YU87-1/BK | 8 | 138.3 | 3.55 | 0.78 | 8.4 | 169.1-169.1 | 168.5-168.9 | 169.3-169.6 | *Trans* |
| chr7 | YU87-1/BK | 9 | 130.4 | 2.93 | -0.76 | 6.94 | 153.4-153.4 | 152.1-152.1 | 153.6-153.6 | *Trans* |
| chr7 | ZONG3/YU87-1 | 3 | 6.5 | 2.98 | -0.74 | 6.19 | 1.6-1.6 | 1.6-1.6 | 2.1-2.1 | *Trans* |
| chr7 | ZONG3/YU87-1 | 4 | 173.9 | 2.61 | -0.63 | 4.55 | 185.4-185.4 | 185.2-185.2 | 186.2-186.2 | *Trans* |
| chr7 | ZONG3/YU87-1 | 4 | 186.2 | 3.4 | -0.72 | 5.88 | 191.6-191.6 | 191.4-191.4 | 198.1-198.1 | *Trans* |
| chr7 | ZONG3/YU87-1 | 5 | 308 | 3.3 | 0.72 | 5.75 | 215.9-215.9 | 215.6-215.6 | 216.7-216.8 | *Trans* |
| chr8 | B73/BY804 | 6 | 74.6 | 5.01 | -1.32 | 10.49 | 142.3-142.5 | 141.7-142.3 | 142.6-142.7 | *Trans* |
| chr8 | B73/BY804 | 6 | 87 | 2.9 | 0.95 | 5.57 | 148.7-148.7 | 147.7-147.7 | 149-149 | *Trans* |
| chr8 | K22/BY815 | 3 | 159.5 | 6.01 | 1.01 | 11.97 | 217.7-217.8 | 217.6-217.6 | 218.2-218.2 | *Trans* |
| chr8 | K22/BY815 | 6 | 103.4 | 2.91 | 0.56 | 5.62 | 162.5-162.5 | 160.2-160.2 | 162.7-162.7 | *Trans* |
| chr8 | KUI3/B77 | 3 | 104.6 | 2.59 | -0.48 | 5.7 | 179.3-179.3 | 173.6-175.2 | 181.7-181.7 | *Trans* |
| chr8 | KUI3/B77 | 5 | 75.2 | 3.31 | 0.69 | 7.13 | 47.6-54.8 | 42.9-54.4 | 63.3-64.7 | *Trans* |
| chr8 | K22/CI7 | 1 | 187.5 | 3.36 | 0.55 | 6.95 | 243.4-243.5 | 241.5-241.5 | 245.3-245.5 | *Trans* |
| chr8 | DAN340/K22 | 3 | 3 | 4.96 | -0.87 | 10.15 | 6.3-6.3 | 5.4-5.4 | 6.6-6.6 | *Trans* |
| chr8 | KUI3/SC55 | 3 | 32.5 | 2.63 | -0.54 | 5.43 | 6.8-6.9 | 6月6日 | 7.1-7.1 | *Trans* |
| chr8 | KUI3/SC55 | 4 | 47.2 | 3.47 | 0.64 | 7.29 | 18-18 | 16.5-17.2 | 18.1-18.1 | *Trans* |
| chr8 | ZHENG58/SK | 2 | 79.5 | 2.62 | -0.49 | 4.88 | 23.9-24 | 22-22.7 | 27-27.1 | *Trans* |
| chr8 | ZHENG58/SK | 6 | 149 | 4.25 | -0.61 | 7.99 | 166.8-166.8 | 165.9-166.1 | 167.1-167.4 | *Trans* |
| chr8 | YU87-1/BK | 3 | 24.6 | 3.62 | -0.6 | 8.41 | 3.6-3.6 | 3.4-3.5 | 3.8-3.8 | *Trans* |
| chr8 | YU87-1/BK | 9 | 47.9 | 2.83 | -0.51 | 6.43 | 17.8-17.8 | 15.9-15.9 | 19.7-19.7 | *Trans* |
| chr8 | ZONG3/YU87-1 | 2 | 203.9 | 3.34 | 0.65 | 6.12 | 188.4-188.4 | 187.8-188.1 | 189.3-189.4 | *Trans* |
| chr8 | ZONG3/YU87-1 | 5 | 169.6 | 5.7 | 0.97 | 10.75 | 174.3-174.6 | 174.1-174.3 | 175.2-175.2 | *Trans* |
| chr8 | ZONG3/YU87-1 | 5 | 191 | 3.33 | -0.78 | 6.3 | 184.3-184.3 | 181-181.4 | 184.4-184.4 | *Trans* |
| chr8 | ZONG3/YU87-1 | 5 | 198.8 | 4.59 | -0.87 | 8.55 | 186.7-186.7 | 186.7-186.7 | 187.1-188.5 | *Trans* |
| chr8 | ZONG3/YU87-1 | 5 | 204.6 | 4.1 | -0.8 | 8.11 | 188.8-188.8 | 188.8-188.8 | 189.8-189.8 | *Trans* |
| chr8 | ZONG3/YU87-1 | 5 | 288.3 | 2.65 | 0.59 | 4.84 | 213-213 | 212.8-212.8 | 213.3-213.3 | *Trans* |
| chr8 | ZONG3/YU87-1 | 7 | 108.2 | 2.65 | 0.69 | 4.52 | 121.3-121.3 | 118.3-118.4 | 121.8-121.8 | *Trans* |
| chr9 | B73/BY804 | 1 | 62.7 | 2.75 | 0.64 | 5.35 | 28.5-28.6 | 26.9-26.9 | 30.3-30.7 | *Trans* |
| chr9 | B73/BY804 | 1 | 70.8 | 3.3 | 0.54 | 6.97 | 41.4-42.8 | 40.4-40.4 | 43.8-43.8 | *Trans* |
| chr9 | B73/BY804 | 5 | 131.9 | 3.43 | 0.51 | 6.19 | 196.6-196.6 | 196-196.3 | 198-198 | *Trans* |
| chr9 | B73/BY804 | 5 | 140.1 | 2.71 | 0.46 | 4.91 | 201.6-201.9 | 200.1-200.5 | 202.4-203 | *Trans* |
| chr9 | DE3/BY815 | 3 | 19.1 | 3.3 | -0.61 | 5.93 | 3.6-3.6 | 3.4-3.5 | 3.7-3.8 | *Trans* |
| chr9 | DE3/BY815 | 4 | 138.9 | 2.98 | 0.59 | 5.73 | 226.2-226.5 | 200.3-205.4 | 228.6-229.3 | *Trans* |
| chr9 | DE3/BY815 | 7 | 109.6 | 2.88 | 0.56 | 5.17 | 159-159 | 157.6-157.9 | 159.8-159.8 | *Trans* |
| chr9 | K22/BY815 | 7 | 4.4 | 2.62 | -0.56 | 4.15 | 2月2日 | 1.3-1.3 | 3.1-3.2 | *Trans* |
| chr9 | KUI3/B77 | 2 | 156.6 | 3.52 | -0.59 | 7.58 | 213.9-214 | 211.7-211.7 | 214.2-214.6 | *Trans* |
| chr9 | KUI3/B77 | 10 | 39.9 | 3.75 | 0.61 | 8.13 | 10.1-10.1 | 8.9-9.4 | 11.2-11.2 | *Trans* |
| chr9 | BY815/KUI3 | 3 | 118.2 | 4.14 | 0.86 | 8.22 | 174.8-175.6 | 173-173.4 | 176.6-176.6 | *Trans* |
| chr9 | DAN340/K22 | 6 | 5.7 | 3.17 | -0.46 | 6.45 | 4月4日 | 2.9-3.2 | 4.3-4.3 | *Trans* |
| chr9 | DAN340/K22 | 7 | 132 | 4.17 | 0.54 | 8.29 | 170.4-170.6 | 169.8-170.1 | 171.8-171.9 | *Trans* |
| chr9 | DAN340/K22 | 7 | 142.9 | 3.15 | 0.47 | 6.44 | 173.2-173.2 | 172.7-172.7 | 173.3-173.3 | *Trans* |
| chr9 | KUI3/SC55 | 5 | 178.5 | 3.73 | 0.53 | 6.98 | 210.3-210.3 | 208.9-209 | 210.5-210.5 | *Trans* |
| chr9 | KUI3/SC55 | 6 | 9.7 | 4.53 | 0.63 | 9.33 | 9.1-9.5 | 4.6-6 | 13.5-13.5 | *Trans* |
| chr9 | KUI3/SC55 | 6 | 18 | 6.05 | 0.72 | 12.22 | 23.8-24.2 | 14.4-14.4 | 24.6-27.1 | *Trans* |
| chr9 | KUI3/SC55 | 8 | 0 | 2.66 | -0.45 | 5.13 | 0.1-0.3 | 0.1-0.3 | 0.5-0.6 | *Trans* |
| chr9 | KUI3/SC55 | 8 | 9.2 | 4.36 | -0.58 | 8.23 | 2.5-2.5 | 0.8-0.9 | 3.4-3.4 | *Trans* |
| chr9 | KUI3/SC55 | 8 | 14.3 | 3.14 | -0.5 | 6.14 | 4.4-4.4 | 3.9-3.9 | 4.5-4.5 | *Trans* |
| chr9 | YU87-1/BK | 3 | 130.3 | 4.08 | 0.68 | 9.1 | 191-191.3 | 189.8-189.8 | 194.5-194.5 | *Trans* |
| chr9 | YU87-1/BK | 4 | 126.8 | 3.32 | 0.61 | 7.42 | 0-0 | 181.4-181.4 | 187.4-188.3 | *Trans* |
| chr9 | ZONG3/YU87-1 | 1 | 318.4 | 3.1 | 0.61 | 5.93 | 260.7-260.7 | 259-259 | 262.2-262.2 | *Trans* |
| chr9 | ZONG3/YU87-1 | 1 | 329 | 2.8 | -0.78 | 5.01 | 267-267.4 | 266.2-267 | 267.9-267.9 | *Trans* |
| chr9 | ZONG3/YU87-1 | 3 | 150.7 | 2.65 | -0.54 | 4.63 | 173.6-174.8 | 173-173.1 | 175.8-175.9 | *Trans* |
| chr9 | ZONG3/YU87-1 | 8 | 211.6 | 2.81 | 0.58 | 5.41 | 172.4-172.4 | 171.9-171.9 | 172.5-172.7 | *Trans* |
| chr9 | ZONG3/YU87-1 | 8 | 221.3 | 3.44 | 0.63 | 6.34 | 173.6-173.7 | 172.9-173 | 174-174.3 | *Trans* |
| Total | B73/BY804 | 1 | 21.2 | 2.65 | 1.97 | 5.14 | 7-7.3 | 5.4-5.4 | 8.9-8.9 | Total |
| Total | B73/BY804 | 5 | 146.5 | 4.61 | 3.39 | 9.83 | 204.1-204.3 | 203.8-204.1 | 205.1-205.1 | Total |
| Total | DE3/BY815 | 5 | 64.6 | 3.12 | -3.09 | 5.97 | 16.2-16.2 | 14.6-14.7 | 19.2-19.2 | Total |
| Total | K22/BY815 | 1 | 105.1 | 3.62 | 2.93 | 7.18 | 82-82 | 78.7-80.2 | 89.2-89.2 | Total |
| Total | K22/BY815 | 3 | 63.4 | 3.13 | 2.89 | 5.94 | 13.3-13.5 | 13.1-13.1 | 15.3-16.6 | Total |
| Total | K22/BY815 | 3 | 74.3 | 2.82 | 2.56 | 5.39 | 23.1-23.4 | 20.4-20.6 | 23.8-24.9 | Total |
| Total | BY815/KUI3 | 2 | 72.7 | 2.91 | -3.64 | 5.57 | 27.5-27.6 | 24.8-24.8 | 27.8-27.9 | Total |
| Total | BY815/KUI3 | 2 | 83.9 | 5.99 | 5.35 | 12.2 | 36.9-36.9 | 36.8-36.9 | 37.1-38.1 | Total |
| Total | K22/CI7 | 1 | 212.8 | 2.97 | -2.24 | 5.9 | 271.3-271.3 | 266.2-266.2 | 274.9-274.9 | Total |
| Total | K22/CI7 | 1 | 232.4 | 3.34 | 2.75 | 6.83 | 283.4-283.4 | 281.9-281.9 | 285.3-286.3 | Total |
| Total | DAN340/K22 | 2 | 93.1 | 3.12 | 2.27 | 5.53 | 161.7-161.7 | 153.1-153.9 | 170.4-176.5 | Total |
| Total | DAN340/K22 | 3 | 64.8 | 3.9 | -3.46 | 7.25 | 129.1-129.1 | 127.9-127.9 | 133.2-134.1 | Total |
| Total | DAN340/K22 | 7 | 14.5 | 3.96 | -2.63 | 7.97 | 5-5.1 | 1.3-1.3 | 5.5-5.5 | Total |
| Total | KUI3/SC55 | 4 | 123.6 | 3.96 | -2.34 | 7.93 | 186.3-186.6 | 185.6-185.7 | 187.4-187.4 | Total |
| Total | KUI3/SC55 | 4 | 163.5 | 3.9 | 2.35 | 7.94 | 236.9-236.9 | 236.5-236.5 | 237.3-237.3 | Total |
| Total | ZHENG58/SK | 2 | 171.5 | 2.59 | -2.15 | 4.52 | 224.5-228.3 | 223.3-223.7 | 226.4-227 | Total |
| Total | ZHENG58/SK | 3 | 96.2 | 3.37 | -2.49 | 6.04 | 153.8-155.7 | 151.3-151.9 | 156.5-156.5 | Total |
| Total | ZHENG58/SK | 4 | 67.6 | 3.26 | 2.43 | 5.71 | 29.8-30.8 | 28.1-29.2 | 31.7-31.7 | Total |
| Total | YU87-1/BK | 2 | 151.9 | 2.6 | -2.64 | 5.32 | 209-209 | 206.9-207.2 | 209.1-209.1 | Total |
| Total | YU87-1/BK | 2 | 173.1 | 3.9 | 3.32 | 8.48 | 225.8-225.8 | 223.2-223.2 | 226.4-226.7 | Total |
| Total | YU87-1/BK | 2 | 182.5 | 2.98 | 3.1 | 7.93 | 232.1-232.1 | 230-230.9 | 232.1-232.1 | Total |
| Total | YU87-1/BK | 3 | 35.6 | 2.71 | -2.49 | 5.62 | 5.6-5.7 | 4.7-4.7 | 6.1-6.3 | Total |
| Total | YU87-1/BK | 4 | 120.2 | 3.92 | 3.03 | 8.56 | 179.6-179.6 | 178.8-179.6 | 180.2-180.2 | Total |
| Total | YU87-1/BK | 4 | 129.9 | 4.85 | 3.41 | 10.76 | 185.7-185.7 | 181.4-181.4 | 187.4-188.3 | Total |
| Total | YU87-1/BK | 4 | 136.5 | 4.68 | 3.26 | 9.91 | 189.2-189.2 | 187.4-188.3 | 190-190 | Total |
| Total | ZONG3/YU87-1 | 3 | 2.3 | 3.5 | -3.42 | 6.1 | 1.5-1.5 | 1.3-1.3 | 1.7-1.8 | Total |

a is the recombination events of each and total chromosome; b, mapped QTL type, details information could be found from methods section.

**Supplementary Table 2.** Summary of recombination QTL mapping in 23 DH populations

| Trait^a^ | Pop | Chr | Peak_gen (cM) | LOD | Additive | *R2* (%) | Peak_bin (Mb) | Left_bin (Mb) | Right_bin (Mb) | QTL_type^b^ |
| --- | --- | --- | --- | --- | --- | --- | --- | --- | --- | --- |
| chr1 | CFD002 | 9 | 99.4 | 2.63 | 0.34 | 10.86 | 151.6-151.7 | 149.2-149.3 | 152.1-152.1 | *Trans* |
| chr1 | CFD003 | 1 | 82 | 3.62 | 0.62 | 12.98 | 201.5-201.6 | 200.1-200.1 | 202-204.1 | *Cis* |
| chr1 | CFD004 | 1 | 99 | 3.59 | -0.44 | 12.83 | 109.6-103.3 | 92.2-95.1 | 122.9-128.4 | *Cis* |
| chr1 | CFD004 | 2 | 76.6 | 2.94 | 0.34 | 7.78 | 114.5-124.9 | 83.6-83.2 | 137.9-142.5 | *Trans* |
| chr1 | CFD005 | 8 | 5.5 | 3.34 | -0.49 | 13.59 | 3.9-3.9 | 0-1.5 | 4.3-4.4 | *Trans* |
| chr1 | CFD006 | 3 | 74.1 | 3.13 | 0.43 | 10.49 | 177.1-178.3 | 176.3-176.5 | 177-178.8 | *Trans* |
| chr1 | CFD007 | 1 | 79.7 | 3.3 | 0.42 | 10.03 | 77.5-80.7 | 73.3-73.3 | 81.2-82.6 | *Cis* |
| chr1 | CFD007 | 4 | 38.7 | 5.34 | -0.57 | 17.77 | 14.6-14.6 | 12.9-13.2 | 17-17.3 | *Trans* |
| chr1 | CFD009 | 1 | 55.9 | 2.9 | 0.44 | 4.36 | 52.6-52.9 | 48.5-48.5 | 52.3-53.3 | *Cis* |
| chr1 | CFD009 | 1 | 191.9 | 3.29 | 0.46 | 4.89 | 298.8-299.5 | 298.3-298.3 | 300-300.5 | *Cis* |
| chr1 | CFD009 | 2 | 38.4 | 3.44 | 0.62 | 6.54 | 14.3-139.7 | 13.4-13.4 | 18.3-19 | *Trans* |
| chr1 | CFD009 | 2 | 47.9 | 2.79 | 0.52 | 5.56 | 19.8-19.8 | 18.3-19 | 20-20 | *Trans* |
| chr1 | CFD010 | 1 | 70.3 | 2.89 | 0.28 | 10.59 | 187.6-188.2 | 164.6-172.1 | 195.7-195.9 | *Cis* |
| chr1 | CFD011 | 5 | 93.7 | 2.83 | -0.42 | 10.57 | 191.7-191.8 | 191.1-191.6 | 193.7-194.2 | *Trans* |
| chr1 | CFD011 | 5 | 99.7 | 3.41 | -0.46 | 12.58 | 195.5-195.7 | 193.7-194.2 | 196.6-198.2 | *Trans* |
| chr1 | CFD011 | 5 | 105.7 | 3.07 | -0.44 | 11.43 | 198.8-198.8 | 196.6-198.2 | 201.6-204.9 | *Trans* |
| chr1 | CFD012 | 7 | 110.2 | 3.97 | 0.46 | 11.88 | 169.3-169.4 | 168.9-168.9 | 172.8-172.8 | *Trans* |
| chr1 | CFD012 | 8 | 90.8 | 4.28 | -0.49 | 12.98 | 164-164.1 | 162.6-163.5 | 165.3-165.3 | *Trans* |
| chr1 | CFF001 | 1 | 104.9 | 2.68 | 0.54 | 8.28 | 119.8-122.5 | 112.6-119.4 | 123.3-152.1 | *Cis* |
| chr1 | CFF001 | 1 | 115.2 | 5.73 | 0.57 | 18.72 | 172.9-176.8 | 170.5-172.9 | 191.1-193.6 | *Cis* |
| chr1 | CFF002 | 4 | 57.5 | 3.21 | 0.5 | 9.74 | 30.7-31.1 | 22.5-25.3 | 37-42.2 | *Trans* |
| chr1 | CFF002 | 4 | 91.3 | 2.82 | -0.47 | 8.59 | 173.2-173.4 | 163.3-165.7 | 173.8-173.8 | *Trans* |
| chr1 | CFF003 | 1 | 193.8 | 5.52 | -1.86 | 29.61 | 222.7-223.8 | 222.7-223.8 | 224.3-228.1 | *Cis* |
| chr1 | CFF003 | 4 | 79 | 3 | 0.51 | 8.38 | 157.6-158.1 | 156.1-156.2 | 161.4-161.6 | *Trans* |
| chr1 | CFF003 | 4 | 84.7 | 3.18 | 0.52 | 8.86 | 161.9-162.3 | 161.4-161.6 | 166-166 | *Trans* |
| chr1 | CFF003 | 7 | 9.2 | 2.87 | 0.49 | 8 | 2.1-2.1 | 1.3-1.3 | 2.1-2.1 | *Trans* |
| chr1 | CFF003 | 7 | 17.8 | 3.08 | 0.51 | 8.56 | 2.6-2.7 | 2.1-2.1 | 4.1-4.5 | *Trans* |
| chr1 | CFF004 | 3 | 111.2 | 2.6 | -0.47 | 13.98 | 214.6-215.5 | 211.1-213.8 | 216.3-216.4 | *Trans* |
| chr1 | CFF006 | 1 | 188 | 2.63 | 1.01 | 10.94 | 222.9-223.3 | 214.3-217.2 | 223.8-224.1 | *Cis* |
| chr1 | CFF007 | 1 | 129.3 | 4.96 | -0.59 | 14.85 | 226.3-226.3 | 224.4-224.4 | 226.3-226.3 | *Cis* |
| chr1 | CFF008 | 7 | 11 | 2.69 | -0.4 | 9.23 | 2.5-2.5 | 1.3-1.3 | 3月3日 | *Trans* |
| chr1 | CFF009 | 1 | 139.6 | 3.7 | -0.42 | 10.17 | 211.5-211.5 | 208.2-210.5 | 213.3-214.7 | *Cis* |
| chr1 | CFF010 | 4 | 51.2 | 2.85 | 0.39 | 9.25 | 20.9-21.8 | 18.2-18.2 | 25.8-27 | *Trans* |
| chr1 | CFF010 | 10 | 40 | 3.9 | 0.56 | 12.94 | 7.5-10.3 | 5.6-5.7 | 10.6-11.2 | *Trans* |
| chr1 | CFF013 | 9 | 67.8 | 4.4 | -0.78 | 14.2 | 99.3-100.3 | 89.3-97.9 | 100.3-101.8 | *Trans* |
| chr1 | CFF015 | 7 | 139.3 | 2.68 | -0.37 | 8.57 | 172.8-172.8 | 171.2-171.5 | 173.8-173.9 | *Trans* |
| chr2 | CFD001 | 2 | 41.8 | 2.92 | -0.41 | 10.62 | 14.5-15.1 | 13.8-13.9 | 19.2-20 | *Cis* |
| chr2 | CFD001 | 4 | 51.7 | 2.87 | 0.43 | 12.48 | 80.1-85.3 | 70.6-73 | 90.1-92.5 | *Trans* |
| chr2 | CFD002 | 8 | 93.2 | 2.88 | 0.28 | 13.81 | 164.1-164.1 | 162.2-163.2 | 164.8-164.9 | *Trans* |
| chr2 | CFD003 | 2 | 67 | 4.69 | 0.82 | 16.76 | 145.2-152.5 | 144.2-145 | 160-170.5 | *Cis* |
| chr2 | CFD004 | 2 | 91.5 | 3.52 | -1.01 | 10.68 | 182.9-183.4 | 182.3-182.6 | 185-185.2 | *Cis* |
| chr2 | CFD005 | 3 | 58.3 | 3.89 | -0.41 | 15.83 | 173.6-174.8 | 172.3-172.3 | 178.1-183.3 | *Trans* |
| chr2 | CFD005 | 3 | 71.2 | 3.21 | -0.36 | 13.37 | 187-187.4 | 186-186 | 188.5-189.3 | *Trans* |
| chr2 | CFD006 | 4 | 73.4 | 2.71 | -0.29 | 8.39 | 170.9-170.9 | 161.6-166 | 172.1-172.2 | *Trans* |
| chr2 | CFD006 | 7 | 24.3 | 3.79 | -0.35 | 12 | 9.5-13.6 | 9.2-9.3 | 27.8-38.2 | *Trans* |
| chr2 | CFD007 | 3 | 80.5 | 3.61 | -0.39 | 12.41 | 14.6-170.8 | 167.8-168.7 | 172.7-175.4 | *Trans* |
| chr2 | CFD009 | 2 | 84.6 | 3.25 | -1.6 | 19.7 | 192.4-194.1 | 189.6-192.4 | 221-221 | *Cis* |
| chr2 | CFD010 | 9 | 68.5 | 3.8 | -0.47 | 11.85 | 139.2-139.4 | 136.9-138.9 | 142.8-142.8 | *Trans* |
| chr2 | CFD012 | 3 | 63.2 | 3.19 | -0.38 | 10.15 | 117.8-118.2 | 113.5-115.5 | 120.2-121.7 | *Trans* |
| chr2 | CFF001 | 2 | 124.3 | 3.63 | 0.63 | 11.54 | 188.4-188.9 | 187.6-187.6 | 189.3-189.6 | *Cis* |
| chr2 | CFF001 | 2 | 137 | 5.5 | -0.81 | 19.21 | 207.8-208.9 | 206.1-207.4 | 211.8-212.4 | *Cis* |
| chr2 | CFF002 | 10 | 77.6 | 4.24 | 0.92 | 14.54 | 135.7-136.6 | 135-135 | 142.1-142.1 | *Trans* |
| chr2 | CFF004 | 5 | 49.1 | 3.72 | -0.49 | 20.79 | 20.8-20.8 | 21.4-22.7 | 23.5-42.2 | *Trans* |
| chr2 | CFF004 | 6 | 137.9 | 4.53 | -0.48 | 18.34 | 165.1-165.6 | 164.5-164.8 | 166.5-166.6 | *Trans* |
| chr2 | CFF004 | 7 | 5 | 3.7 | 0.42 | 16 | 1.3-2 | 0-1 | 2.5-2.5 | *Trans* |
| chr2 | CFF006 | 2 | 8.3 | 3.78 | -1.33 | 15.79 | 4.1-4.1 | 3.7-3.7 | 4.1-4.1 | *Cis* |
| chr2 | CFF006 | 2 | 78.6 | 5.44 | -1.39 | 31.54 | 41.9-42.4 | 37.2-41.5 | 43.2-43.7 | *Cis* |
| chr2 | CFF006 | 2 | 87.2 | 3.45 | -1.04 | 22.02 | 55.8-60.3 | 43.2-43.2 | 63.9-68.6 | *Cis* |
| chr2 | CFF008 | 3 | 60.7 | 2.69 | -0.53 | 11.55 | 21.6-25.5 | 21.4-21.4 | 27.6-36.7 | *Trans* |
| chr2 | CFF008 | 6 | 50.1 | 4.05 | -0.66 | 17.87 | 109.1-111.9 | 108.3-108.3 | 125.4-127.9 | *Trans* |
| chr2 | CFF009 | 1 | 93.4 | 2.69 | -0.31 | 7.42 | 91.2-91.2 | 88.3-88.3 | 107.2-107.4 | *Trans* |
| chr2 | CFF009 | 7 | 0 | 3.15 | 0.49 | 8.76 | 0-1 | 0-1 | 0.7-0.8 | *Trans* |
| chr2 | CFF010 | 7 | 60.5 | 2.93 | 0.35 | 10.43 | 121.2-124.4 | 117.4-121 | 127.3-127.9 | *Trans* |
| chr3 | CFD001 | 3 | 45.9 | 6.55 | 1.32 | 34.57 | 9.6-10.1 | 8.2-9.1 | 10.4-10.5 | *Cis* |
| chr3 | CFD001 | 3 | 54.1 | 5.62 | 0.71 | 23.35 | 16.8-18.2 | 12.6-14 | 25.3-26.5 | *Cis* |
| chr3 | CFD001 | 3 | 63.9 | 4.04 | 0.63 | 17.48 | 85.7-92.1 | 60.7-65.7 | 102.1-107.5 | *Cis* |
| chr3 | CFD001 | 8 | 51.6 | 2.91 | 0.37 | 5.94 | 78.4-79 | 72.5-72.6 | 96.3-99.3 | *Trans* |
| chr3 | CFD001 | 10 | 106.2 | 2.73 | 0.32 | 4.8 | 144.8-145 | 142.8-142.8 | 145.6-147.1 | *Trans* |
| chr3 | CFD001 | 10 | 117.1 | 2.99 | 0.34 | 5.21 | 148.1-148.1 | 147.5-147.7 | 148.9-220.4 | *Trans* |
| chr3 | CFD003 | 3 | 49.3 | 6.05 | 0.96 | 23.86 | 18.2-18.2 | 20.4-22.4 | 23.1-32.1 | *Cis* |
| chr3 | CFD004 | 3 | 37.9 | 6.06 | 2.1 | 13.2 | 24.9-28.3 | 24-24.9 | 28.6-28.8 | *Cis* |
| chr3 | CFD004 | 3 | 44.8 | 7.85 | -2.57 | 24.22 | 48-48 | 48-48 | 49.4-52.7 | *Cis* |
| chr3 | CFD004 | 3 | 49.8 | 8.12 | -2.83 | 25.5 | 116.4-117.2 | 116.4-117.2 | 126.2-126.2 | *Cis* |
| chr3 | CFD004 | 3 | 57.6 | 2.66 | 1.35 | 10.49 | 141-141.7 | 137.7-139.7 | 141.7-148 | *Cis* |
| chr3 | CFD006 | 3 | 33.6 | 4.05 | 0.92 | 2.23 | 6.3-6.3 | 5.7-5.8 | 6.9-7.5 | *Cis* |
| chr3 | CFD006 | 3 | 40.5 | 33.43 | 5.67 | 58.19 | 12.6-17.9 | 9-9.1 | 12.6-17.9 | *Cis* |
| chr3 | CFD006 | 3 | 49.5 | 9.25 | 3.05 | 23.82 | 32.0-36.6 | 19.8-25.1 | 43.2-43.2 | *Cis* |
| chr3 | CFD006 | 3 | 52.5 | 8.02 | 1.69 | 9.67 | 156.5-156.9 | 110.5-117.7 | 159.8-164.6 | *Cis* |
| chr3 | CFD006 | 3 | 61.9 | 2.76 | 0.74 | 1.81 | 172.5-172.6 | 167.5-171.4 | 170.1-173.4 | *Cis* |
| chr3 | CFD009 | 3 | 36.1 | 3.64 | 0.74 | 16.18 | 9.6-11.2 | 7-7.6 | 12.6-12.6 | *Cis* |
| chr3 | CFD009 | 5 | 116.2 | 3.53 | -0.41 | 8.17 | 211.1-211.1 | 210-210 | 210.7-211.7 | *Trans* |
| chr3 | CFD010 | 3 | 55 | 5.63 | -0.62 | 19.69 | 17.3-19.8 | 17.4-20.2 | 26-37.1 | *Cis* |
| chr3 | CFD011 | 3 | 53.8 | 10.04 | 1.09 | 40.28 | 52.3-54.5 | 52-52.2 | 52.3-54.5 | *Cis* |
| chr3 | CFD011 | 3 | 63.9 | 6.06 | 1.33 | 28.94 | 156.9-157.1 | 149.2-149.4 | 157.1-157.5 | *Cis* |
| chr3 | CFD012 | 3 | 82.3 | 3.02 | 1.16 | 15.67 | 128.1-128.1 | 120.8-121.9 | 128.1-128.1 | *Cis* |
| chr3 | CFF001 | 3 | 45 | 7.21 | -1.02 | 28.6 | 21.1-21.7 | 21.6-21.7 | 31.4-33.8 | *Cis* |
| chr3 | CFF002 | 10 | 82.9 | 2.77 | 0.62 | 9.96 | 140.2-141.2 | 136.8-136.8 | 141.4-141.4 | *Trans* |
| chr3 | CFF003 | 1 | 263.7 | 4.62 | 0.53 | 14.93 | 289.2-289.5 | 287-287.1 | 291.5-291.9 | *Trans* |
| chr3 | CFF003 | 6 | 29.7 | 2.61 | 0.3 | 8.08 | 32.5-34.6 | 30.1-32.5 | 68.2-72.8 | *Trans* |
| chr3 | CFF004 | 1 | 37.7 | 3.03 | 0.54 | 9.32 | 33.1-33.1 | 22.3-25.6 | 35-35.1 | *Trans* |
| chr3 | CFF004 | 10 | 66.6 | 3.16 | -0.54 | 13.76 | 130.6-133.1 | 130.3-130.5 | 135-135 | *Trans* |
| chr3 | CFF004 | 10 | 76.7 | 4.06 | -0.59 | 17.03 | 137.5-138.3 | 133.7-136.1 | 140.2-140.2 | *Trans* |
| chr3 | CFF006 | 3 | 64.6 | 4.22 | -1.14 | 15.78 | 149.6-150.4 | 137.8-147.2 | 154.1-154.1 | *Cis* |
| chr3 | CFF006 | 3 | 74.7 | 4.34 | -1.12 | 16.19 | 156.7-156.8 | 153-153.9 | 157.2-157.8 | *Cis* |
| chr3 | CFF006 | 3 | 79.8 | 4.48 | -1.33 | 19.06 | 162.4-165.7 | 157.2-157.8 | 162.6-165.2 | *Cis* |
| chr3 | CFF006 | 6 | 3.7 | 4.63 | 1.03 | 17.57 | 2.9-20 | 0.7-2 | 25.5-39.4 | *Trans* |
| chr3 | CFF006 | 7 | 110 | 6.01 | 1.23 | 24.52 | 141.6-144.8 | 125.6-141.5 | 154.3-158.9 | *Trans* |
| chr3 | CFF006 | 8 | 130.9 | 5.98 | 1.23 | 24.37 | 171.8-172 | 171.2-171.5 | 172.2-172.6 | *Trans* |
| chr3 | CFF007 | 10 | 4 | 3.06 | 0.29 | 8.46 | 136-136.1 | 133.7-134.5 | 137.2-137.2 | *Trans* |
| chr3 | CFF008 | 3 | 119.3 | 4.9 | -0.51 | 15.62 | 209-210.4 | 202.8-202.9 | 212.8-213.4 | *Cis* |
| chr3 | CFF008 | 4 | 49.3 | 2.75 | -0.37 | 9.37 | 21.7-31.1 | 19.1-21.7 | 37.1-37.3 | *Trans* |
| chr3 | CFF008 | 4 | 60.7 | 5.06 | -0.5 | 16.89 | 72.9-82.6 | 72.1-72.2 | 83-83.9 | *Trans* |
| chr3 | CFF008 | 5 | 50.7 | 2.88 | -0.5 | 9.51 | 15.2-16.6 | 14.7-15.3 | 17.1-17.9 | *Trans* |
| chr3 | CFF008 | 5 | 61.5 | 4.51 | -0.53 | 14.2 | 61.7-70.8 | 55.2-60.5 | 67.4-74.9 | *Trans* |
| chr3 | CFF008 | 7 | 88.5 | 3.37 | 0.41 | 10.93 | 141.5-145.5 | 139.6-139.6 | 146.5-146.5 | *Trans* |
| chr3 | CFF008 | 7 | 93.7 | 4.45 | 0.46 | 13.99 | 158.3-160.4 | 156-157.9 | 164.2-165 | *Trans* |
| chr3 | CFF009 | 1 | 135.7 | 3.36 | -0.37 | 8.64 | 207.6-208.2 | 202.6-205.7 | 208.2-210.5 | *Trans* |
| chr3 | CFF009 | 1 | 144.6 | 3.75 | -0.4 | 9.77 | 215.2-217.5 | 211.5-213.1 | 231.2-231.4 | *Trans* |
| chr3 | CFF010 | 3 | 4.8 | 2.8 | 0.37 | 9.71 | 2.2-2.6 | 1.6-1.6 | 2.6-2.6 | *Cis* |
| chr3 | CFF010 | 3 | 12.9 | 3.73 | 0.46 | 12.67 | 3.5-3.6 | 2.6-2.6 | 3.7-3.8 | *Cis* |
| chr3 | CFF010 | 3 | 19.6 | 3.46 | 0.51 | 11.82 | 4.7-4.7 | 4.4-4.4 | 4.9-4.9 | *Cis* |
| chr3 | CFF013 | 10 | 9.6 | 6.75 | -0.53 | 22.84 | 2.3-2.3 | 2.2-2.2 | 2.4-2.5 | *Trans* |
| chr3 | CFF015 | 4 | 39.2 | 8.48 | 0.74 | 26.62 | 17.4-17.4 | 16.6-17.3 | 17.8-18.2 | *Trans* |
| chr3 | CFF015 | 4 | 47.2 | 6.44 | 0.61 | 19.98 | 36.9-38 | 35.2-36.5 | 38.8-43.1 | *Trans* |
| chr4 | CFD001 | 4 | 58.7 | 4.07 | 0.27 | 11.36 | 150.8-151.7 | 147.1-147.7 | 151.7-152.2 | *Cis* |
| chr4 | CFD001 | 5 | 132.5 | 3.5 | -0.25 | 9.58 | 206.6-206.7 | 205.6-205.8 | 208.1-208.1 | *Trans* |
| chr4 | CFD001 | 6 | 91.8 | 3.83 | 0.26 | 10.89 | 156.8-157 | 156.5-156.5 | 157.3-158.5 | *Trans* |
| chr4 | CFD001 | 7 | 82.3 | 3.05 | -0.31 | 8.26 | 125.1-127.6 | 123.8-124.2 | 128.1-128.6 | *Trans* |
| chr4 | CFD001 | 7 | 97.9 | 2.75 | 0.33 | 8.45 | 136.3-136.3 | 134-137.5 | 135.9-149.9 | *Trans* |
| chr4 | CFD001 | 7 | 107.5 | 5.25 | 0.38 | 15.09 | 160.4-160.8 | 157.5-159.7 | 161.8-161.8 | *Trans* |
| chr4 | CFD002 | 4 | 16.2 | 3.62 | -0.35 | 13.81 | 7-10.5 | 5.2-5.3 | 4.1-13.2 | *Cis* |
| chr4 | CFD002 | 4 | 24.8 | 4.28 | -0.38 | 16.01 | 14.5-14.6 | 4.1-13.2 | 15.6-17.1 | *Cis* |
| chr4 | CFD002 | 8 | 21.2 | 5.08 | 0.42 | 19.49 | 8.6-8.6 | 6.1-6.9 | 9.9-11.2 | *Trans* |
| chr4 | CFD003 | 10 | 29.9 | 3.62 | -0.35 | 12.51 | 9.4-10.3 | 7.6-7.6 | 11.8-14.5 | *Trans* |
| chr4 | CFD004 | 3 | 69.6 | 3.16 | 0.3 | 10.38 | 166.8-168.5 | 162.2-164.6 | 176.7-178.3 | *Trans* |
| chr4 | CFD004 | 7 | 97.9 | 3.8 | 0.34 | 12.47 | 160.8-162.3 | 143.1-154.9 | 163-163 | *Trans* |
| chr4 | CFD004 | 9 | 44.7 | 3.03 | 0.46 | 9.92 | 25.5-26.6 | 24.4-25.3 | 26.8-26.8 | *Trans* |
| chr4 | CFD006 | 3 | 14.6 | 3.19 | 0.3 | 8.29 | 3.5-3.5 | 2.6-2.9 | 4.7-4.7 | *Trans* |
| chr4 | CFD006 | 4 | 62.1 | 3.32 | 0.32 | 9.39 | 26.1-29.3 | 25.5-26.1 | 32.5-36.5 | *Cis* |
| chr4 | CFD006 | 5 | 0 | 3.11 | 0.31 | 8.07 | 2.1-2.1 | 2.1-2.1 | 5-6.3 | *Trans* |
| chr4 | CFD007 | 1 | 182.5 | 3.85 | -0.57 | 12.83 | 287.7-290.3 | 287.3-287.7 | 291.9-291.9 | *Trans* |
| chr4 | CFD007 | 4 | 37.7 | 3.08 | 0.39 | 9.56 | 14-14 | 12.9-13.2 | 14.6-14.6 | *Cis* |
| chr4 | CFD009 | 2 | 12.8 | 2.68 | -0.39 | 13.68 | 4.4-4.9 | 3.7-4.2 | 7.3-9.1 | *Trans* |
| chr4 | CFD009 | 3 | 21.9 | 3.03 | -0.37 | 14.66 | 3.7-3.9 | 2.7-3.5 | 4.7-4.8 | *Trans* |
| chr4 | CFD009 | 9 | 41.3 | 2.75 | -0.52 | 13.18 | 13-18.2 | 11.6-11.6 | 18.4-20.6 | *Trans* |
| chr4 | CFD010 | 2 | 44.2 | 3.02 | 0.4 | 10.91 | 23.1-23.5 | 16.8-152.3 | 23.7-24.8 | *Trans* |
| chr4 | CFD010 | 2 | 79.4 | 4.07 | -0.51 | 12.75 | 197.2-198.4 | 193.5-195 | 202.6-203.2 | *Trans* |
| chr4 | CFD010 | 2 | 96.8 | 6.38 | 0.72 | 21.39 | 215-215 | 213.2-213.4 | 215.5-215.7 | *Trans* |
| chr4 | CFD010 | 2 | 103 | 6.43 | 0.63 | 21.52 | 221.7-222.7 | 216-216.7 | 226.4-228.9 | *Trans* |
| chr4 | CFD011 | 2 | 120.4 | 2.79 | 0.39 | 10.02 | 209.3-210.3 | 195.4-195.4 | 228.9-228.9 | *Trans* |
| chr4 | CFD011 | 3 | 98.9 | 4.63 | 0.54 | 17.51 | 183.2-184.1 | 182.6-182.8 | 184.9-185.9 | *Trans* |
| chr4 | CFD011 | 3 | 143.9 | 2.59 | -0.39 | 9.25 | 219.8-220.5 | 218-218.5 | 221.5-221.7 | *Trans* |
| chr4 | CFF002 | 1 | 97.6 | 2.63 | -0.41 | 8.18 | 71-71 | 67-69.3 | 72.8-73.4 | *Trans* |
| chr4 | CFF003 | 4 | 141.2 | 3.39 | 0.44 | 11.11 | 234.1-234.8 | 231.7-232.9 | 235.7-235.7 | *Cis* |
| chr4 | CFF003 | 4 | 175.5 | 3.71 | -0.45 | 12.26 | 240.2-240.2 | 239.1-239.1 | 240.5-240.5 | *Cis* |
| chr4 | CFF006 | 1 | 34 | 4.18 | 1.23 | 20.34 | 11.5-12.5 | 10.9-11.5 | 13-13 | *Trans* |
| chr4 | CFF006 | 5 | 175.8 | 2.81 | 0.7 | 12.3 | 206.3-206.5 | 204.5-205.4 | 206.6-206.6 | *Trans* |
| chr4 | CFF006 | 8 | 113.5 | 2.9 | -0.74 | 12.89 | 165.7-165.7 | 164.4-165.5 | 166.7-166.7 | *Trans* |
| chr4 | CFF007 | 1 | 68.8 | 4.21 | -0.4 | 11.76 | 38.6-38.6 | 35-35.1 | 39.9-39.9 | *Trans* |
| chr4 | CFF007 | 10 | 0.8 | 2.98 | -0.31 | 8.16 | 134.6-134.7 | 133.7-134.5 | 136.7-136.8 | *Trans* |
| chr4 | CFF008 | 4 | 54.5 | 3.18 | -0.34 | 13.61 | 42.4-42.4 | 31.3-36.5 | 52.9-52.9 | *Cis* |
| chr4 | CFF009 | 4 | 58.8 | 3.75 | -0.41 | 10.1 | 58.3-62.3 | 35.2-37.1 | 63.7-68.2 | *Cis* |
| chr4 | CFF009 | 4 | 82.2 | 4.67 | 0.46 | 12.75 | 169.5-169.6 | 164.1-164.1 | 169.8-170 | *Cis* |
| chr4 | CFF010 | 7 | 76.8 | 2.96 | 0.32 | 9.22 | 137.5-137.5 | 133.1-133.4 | 140.7-141.5 | *Trans* |
| chr4 | CFF010 | 8 | 61.6 | 2.96 | -0.34 | 10.33 | 96.3-101.4 | 75.4-81.2 | 105.2-105.3 | *Trans* |
| chr4 | CFF012 | 1 | 101 | 3.52 | -0.55 | 11.36 | 96.1-96.1 | 80.6-88 | 107.2-107.2 | *Trans* |
| chr4 | CFF012 | 1 | 106.2 | 3.93 | -0.62 | 12.59 | 107.2-107.2 | 96.6-96.6 | 120.4-126.2 | *Trans* |
| chr4 | CFF015 | 2 | 112.5 | 2.92 | 0.34 | 8.38 | 175.4-175.6 | 169.1-169.8 | 177.9-177.9 | *Trans* |
| chr4 | CFF015 | 4 | 34.8 | 2.98 | -0.36 | 9.36 | 15.3-16.4 | 13.4-13.8 | 15.3-16.4 | *Cis* |
| chr4 | CFF015 | 4 | 84.1 | 4.54 | -0.7 | 12.9 | 172.1-172.1 | 171.8-171.8 | 172.8-172.8 | *Cis* |
| chr4 | CFF015 | 4 | 91.5 | 3.44 | -0.49 | 9.98 | 177.3-177.3 | 175.1-175.3 | 177.6-178.5 | *Cis* |
| chr5 | CFD001 | 6 | 72.6 | 3.11 | -0.41 | 11.93 | 147.9-147.9 | 142.3-144 | 148.8-149 | *Trans* |
| chr5 | CFD004 | 5 | 90.3 | 6.72 | 0.94 | 20.61 | 183.6-183.6 | 183.6-183.6 | 184.1-185.5 | *Cis* |
| chr5 | CFD004 | 5 | 101 | 10.43 | -1.15 | 33.16 | 194.2-194.7 | 191.1-194 | 195.4-195.4 | *Cis* |
| chr5 | CFD005 | 1 | 149.4 | 4.12 | 0.57 | 17.14 | 291-291.7 | 288-289.6 | 292.3-292.5 | *Trans* |
| chr5 | CFD005 | 5 | 78.4 | 2.95 | -0.32 | 12.28 | 186.7-186.9 | 180.9-183.5 | 191.1-191.9 | *Cis* |
| chr5 | CFD005 | 5 | 86.2 | 3.6 | -0.36 | 14.71 | 194.2-194.6 | 191.1-191.9 | 201.2-202.8 | *Cis* |
| chr5 | CFD006 | 1 | 0 | 4.94 | 1.53 | 18.73 | 3-3.4 | 2.7-2.9 | 4-4.4 | *Trans* |
| chr5 | CFD006 | 1 | 15.6 | 3.28 | -1.39 | 11.83 | 3.7-4.5 | 2-2.4 | 5.1-6.5 | *Trans* |
| chr5 | CFD009 | 3 | 123.5 | 2.79 | 0.36 | 11.06 | 232-232 | 221.5-224.2 | 232-232 | *Trans* |
| chr5 | CFD010 | 1 | 96.7 | 2.7 | -0.27 | 8.95 | 223-223.2 | 217.9-222.1 | 231.3-235.4 | *Trans* |
| chr5 | CFD010 | 2 | 8.9 | 2.79 | 0.28 | 9.27 | 3.4-3.4 | 2.5-2.5 | 3.7-3.9 | *Trans* |
| chr5 | CFD010 | 5 | 109 | 2.89 | -0.28 | 9.62 | 211.7-211.7 | 209.6-210.3 | 212.2-212.2 | *Cis* |
| chr5 | CFD010 | 8 | 97.7 | 2.73 | -0.27 | 8.79 | 169.8-169.8 | 169.1-169.1 | 170.9-171.5 | *Trans* |
| chr5 | CFD011 | 8 | 10 | 2.88 | 0.37 | 11.33 | 2.9-2.9 | 1.8-2.3 | 5-5.2 | *Trans* |
| chr5 | CFD012 | 9 | 109.1 | 4.34 | 0.39 | 17.36 | 154.3-154.4 | 153.6-153.6 | 154.3-154.4 | *Trans* |
| chr5 | CFF001 | 5 | 38.7 | 4.71 | 0.44 | 15.94 | 13.4-13.6 | 12.6-13.4 | 13.8-13.8 | *Cis* |
| chr5 | CFF001 | 5 | 51 | 7.29 | 0.58 | 23.27 | 24.5-29.7 | 17.1-18.6 | 31.9-32.4 | *Cis* |
| chr5 | CFF001 | 5 | 57.6 | 6.52 | 0.6 | 21.16 | 33.1-42.7 | 31.9-32.4 | 43.1-43.6 | *Cis* |
| chr5 | CFF001 | 5 | 118.7 | 4.28 | 0.38 | 12.92 | 200-200.4 | 199.8-200.4 | 200.8-202.8 | *Cis* |
| chr5 | CFF001 | 5 | 126.9 | 4.64 | 0.39 | 13.9 | 205.4-205.4 | 200.8-202.8 | 205.9-206.7 | *Cis* |
| chr5 | CFF002 | 4 | 68.1 | 2.89 | 0.36 | 8.74 | 150-150.3 | 131.8-131.8 | 150.5-153 | *Trans* |
| chr5 | CFF003 | 2 | 123.8 | 3.14 | -0.44 | 10.95 | 188.1-188.1 | 185.6-186.4 | 190.2-191.1 | *Trans* |
| chr5 | CFF006 | 2 | 163.9 | 2.71 | -0.79 | 12.77 | 228-230.9 | 217.6-228 | 232.9-232.9 | *Trans* |
| chr5 | CFF006 | 5 | 79.7 | 3.57 | -0.92 | 17.48 | 68.5-69.3 | 60.8-68.4 | 69.3-74.3 | *Cis* |
| chr5 | CFF006 | 5 | 88.2 | 3.54 | -0.97 | 17.35 | 82.6-85.7 | 69.3-74.3 | 86-86 | *Cis* |
| chr5 | CFF006 | 8 | 122.1 | 3.67 | 0.9 | 18.01 | 169.5-169.5 | 166.6-166.7 | 171.8-172 | *Trans* |
| chr5 | CFF007 | 5 | 18 | 7.17 | -0.74 | 21.16 | 2.8-3.4 | 2.6-2.6 | 3.5-3.5 | *Cis* |
| chr5 | CFF008 | 1 | 181.5 | 2.65 | -0.35 | 10.29 | 285.1-286.9 | 281.7-285.1 | 288.1-289.5 | *Trans* |
| chr5 | CFF008 | 1 | 191 | 2.89 | -0.35 | 11.08 | 292.1-293.2 | 288.1-289.5 | 294-294.1 | *Trans* |
| chr5 | CFF008 | 7 | 119.2 | 3.03 | 0.34 | 11.72 | 171.9-171.9 | 171.7-171.7 | 172.9-172.9 | *Trans* |
| chr5 | CFF008 | 10 | 62.3 | 4.29 | 0.61 | 17.54 | 29.9-76.5 | 18.7-31 | 77.3-87.1 | *Trans* |
| chr5 | CFF009 | 2 | 171.3 | 3.09 | -0.33 | 6.17 | 233.7-234.4 | 233-233.1 | 234.7-234.7 | *Trans* |
| chr5 | CFF009 | 4 | 144 | 3.14 | -0.35 | 6.72 | 235.3-235.3 | 234.1-234.4 | 235.7-235.7 | *Trans* |
| chr5 | CFF010 | 7 | 38.5 | 3.45 | -0.38 | 11.03 | 7.5-7.5 | 5.6-6.1 | 8.1-8.9 | *Trans* |
| chr5 | CFF010 | 7 | 48.8 | 3.45 | -0.35 | 9.68 | 14-14 | 8.1-8.9 | 20.6-27.4 | *Trans* |
| chr5 | CFF010 | 8 | 74 | 4.7 | -0.4 | 13.72 | 118.2-118.2 | 113.9-117.2 | 131.8-141.8 | *Trans* |
| chr5 | CFF010 | 8 | 83.2 | 2.84 | -0.32 | 8.63 | 148.2-149.9 | 142.7-145.8 | 156.6-159 | *Trans* |
| chr5 | CFF012 | 5 | 73.8 | 2.88 | 0.51 | 9.83 | 151.7-154 | 121.1-123.4 | 154.5-154.8 | *Cis* |
| chr5 | CFF012 | 5 | 104.8 | 7.16 | -0.87 | 24.17 | 155.8-157.2 | 155.8-163.3 | 158.8-164.5 | *Cis* |
| chr5 | CFF015 | 2 | 10.2 | 2.67 | -0.48 | 7.81 | 3.1-3.4 | 3.1-3.1 | 3.7-3.7 | *Trans* |
| chr6 | CFD001 | 6 | 12.1 | 5.25 | 0.92 | 21.69 | 65.5-65.5 | 30.2-30.3 | 72.5-79.6 | *Cis* |
| chr6 | CFD001 | 7 | 73.9 | 3.65 | 0.43 | 11.86 | 120-120.1 | 119.6-119.6 | 121-121.3 | *Trans* |
| chr6 | CFD001 | 7 | 90.7 | 4.8 | 0.47 | 15.16 | 131.1-131.4 | 128.1-128.6 | 134-137.5 | *Trans* |
| chr6 | CFD002 | 4 | 43 | 4.56 | -0.58 | 18.17 | 157-157 | 153.5-153.5 | 159.7-161.7 | *Trans* |
| chr6 | CFD002 | 8 | 112.9 | 3.57 | 0.41 | 14.4 | 170.1-170.2 | 169.5-169.5 | 170.3-170.4 | *Trans* |
| chr6 | CFD002 | 8 | 122.9 | 4.34 | 0.44 | 17.1 | 171.7-172 | 170.6-170.8 | 173.6-173.6 | *Trans* |
| chr6 | CFD003 | 6 | 92.2 | 4.37 | 0.6 | 16.93 | 156.9-157.3 | 154.9-154.9 | 157.6-158.3 | *Cis* |
| chr6 | CFD004 | 7 | 43 | 3.06 | -0.28 | 10.23 | 9.9-10.2 | 8.2-8.7 | 11.4-13.2 | *Trans* |
| chr6 | CFD004 | 7 | 49.8 | 2.71 | -0.27 | 9.16 | 17.7-17.7 | 13.6-13.8 | 29.5-47.9 | *Trans* |
| chr6 | CFD005 | 5 | 88.9 | 2.89 | -0.26 | 11.49 | 195-195.7 | 193.9-194.1 | 202.8-202.8 | *Trans* |
| chr6 | CFD006 | 4 | 154.5 | 2.63 | -0.34 | 5.71 | 239.4-239.5 | 238.3-238.3 | 240.5-241.2 | *Trans* |
| chr6 | CFD006 | 6 | 93.2 | 16.18 | 1.09 | 41.08 | 156.4-156.4 | 155.8-155.8 | 158.8-159.2 | *Cis* |
| chr6 | CFD006 | 6 | 105.9 | 9.82 | -0.9 | 21.34 | 158.8-159.2 | 156-156.3 | 160.6-160.6 | *Cis* |
| chr6 | CFD006 | 6 | 114.5 | 3.23 | -0.6 | 8.09 | 161.9-161.9 | 156.4-157.3 | 162-162 | *Cis* |
| chr6 | CFD007 | 7 | 95.6 | 4.66 | 0.4 | 15.59 | 167.2-168.3 | 166.6-166.6 | 168.6-168.6 | *Trans* |
| chr6 | CFD007 | 7 | 104.3 | 5.35 | 0.43 | 17.65 | 171.7-171.7 | 170.8-170.8 | 171.7-171.7 | *Trans* |
| chr6 | CFD009 | 4 | 112.2 | 4.97 | -0.53 | 22.98 | 215.7-225.9 | 200.2-206 | 228-234.8 | *Trans* |
| chr6 | CFD010 | 3 | 35.8 | 3.92 | 0.58 | 15.09 | 6.3-6.3 | 5.9-5.9 | 6.6-6.6 | *Trans* |
| chr6 | CFD010 | 3 | 50.3 | 2.82 | -0.41 | 10.51 | 12.2-12.3 | 10.6-12.1 | 17.4-20.2 | *Trans* |
| chr6 | CFD010 | 3 | 58.4 | 2.73 | -0.35 | 10.23 | 41.4-47.6 | 21.6-24.4 | 73.8-75.6 | *Trans* |
| chr6 | CFD012 | 2 | 67.8 | 3.62 | 0.38 | 11.55 | 46-46.6 | 41.8-41.8 | 57.1-60.8 | *Trans* |
| chr6 | CFD012 | 5 | 86.9 | 2.67 | -0.46 | 9.29 | 188.8-189.2 | 179-179 | 195-195 | *Trans* |
| chr6 | CFD012 | 8 | 16.6 | 2.77 | -0.28 | 8.66 | 5.3-5.9 | 4.3-4.3 | 9-10.3 | *Trans* |
| chr6 | CFF002 | 8 | 49.7 | 3.15 | -0.41 | 7.99 | 13.6-14.6 | 14.8-14.8 | 15.1-15.5 | *Trans* |
| chr6 | CFF003 | 6 | 72.8 | 4.15 | -1.5 | 14.47 | 105.1-105.1 | 103.4-105.1 | 105.5-105.5 | *Cis* |
| chr6 | CFF004 | 1 | 23.6 | 4.02 | 0.45 | 16.9 | 13-13.7 | 7.7-8.6 | 18-18.2 | *Trans* |
| chr6 | CFF004 | 2 | 73.6 | 2.73 | 0.36 | 10.84 | 58.4-60.1 | 42-45.2 | 62.7-67.5 | *Trans* |
| chr6 | CFF006 | 2 | 112.7 | 3.53 | 1.08 | 16.9 | 167.4-179.4 | 149.2-165.6 | 183.9-186.9 | *Trans* |
| chr6 | CFF006 | 4 | 4.8 | 4.92 | -0.77 | 25.59 | 3-3.9 | 0-1.3 | 5.3-5.3 | *Trans* |
| chr6 | CFF006 | 4 | 122.6 | 4.88 | -0.68 | 25.31 | 182-183.6 | 175.8-175.8 | 183.7-184.9 | *Trans* |
| chr6 | CFF008 | 2 | 65.2 | 2.73 | 0.31 | 11.42 | 23.9-26.5 | 21.8-22.7 | 28.1-28.7 | *Trans* |
| chr6 | CFF012 | 2 | 69.6 | 3.02 | -0.45 | 9.54 | 48.6-53.8 | 41.2-42.4 | 55-55 | *Trans* |
| chr6 | CFF012 | 6 | 126 | 4.49 | -0.63 | 14.55 | 164.5-164.5 | 164-164 | 164.6-164.6 | *Cis* |
| chr6 | CFF013 | 5 | 3 | 4.48 | -0.5 | 14.72 | 2.3-2.3 | 1.6-2.2 | 3.3-3.4 | *Trans* |
| chr7 | CFD001 | 7 | 71.5 | 2.78 | 0.67 | 12.8 | 118.6-118.6 | 109.2-117.4 | 121-121.3 | *Cis* |
| chr7 | CFD001 | 7 | 79.9 | 3.21 | 0.71 | 14.58 | 123.8-124.2 | 122-123.6 | 131.1-131.1 | *Cis* |
| chr7 | CFD002 | 1 | 35.2 | 3.4 | 0.57 | 15.3 | 24.6-24.7 | 21.7-24.5 | 27.6-30.6 | *Trans* |
| chr7 | CFD002 | 7 | 89.9 | 3.04 | 0.42 | 13.32 | 165.7-165.7 | 162.1-162.3 | 166.7-167 | *Cis* |
| chr7 | CFD002 | 7 | 99.3 | 2.72 | 0.4 | 12.09 | 171.2-171.5 | 166.7-167 | 171.2-171.5 | *Cis* |
| chr7 | CFD003 | 7 | 51.4 | 4.86 | 0.82 | 17.18 | 109.3-109.3 | 94.2-107.4 | 109.7-112.4 | *Cis* |
| chr7 | CFD003 | 7 | 69.4 | 4.03 | -0.84 | 20.93 | 63-63 | 63-63 | 85.3-85.3 | *Cis* |
| chr7 | CFD004 | 5 | 59.9 | 2.73 | -0.51 | 8.47 | 58.8-59.4 | 47.6-54.6 | 63.1-64.6 | *Trans* |
| chr7 | CFD004 | 10 | 58.6 | 5.27 | -0.42 | 17.04 | 130.3-130.5 | 113.5-117.8 | 133.8-133.8 | *Trans* |
| chr7 | CFD004 | 10 | 68.4 | 4.07 | -0.38 | 13.49 | 135.9-135.9 | 135.3-135.3 | 136.2-136.5 | *Trans* |
| chr7 | CFD005 | 1 | 26.7 | 2.81 | 0.34 | 11.11 | 16.4-16.8 | 15.2-15.5 | 19.7-21.7 | *Trans* |
| chr7 | CFD005 | 6 | 37.5 | 2.61 | 0.4 | 10.26 | 133.4-135.5 | 126.2-128.1 | 137.6-138.2 | *Trans* |
| chr7 | CFD005 | 6 | 45.4 | 2.81 | 0.52 | 10.97 | 147.9-148 | 138.7-144 | 148.4-150.8 | *Trans* |
| chr7 | CFD007 | 7 | 51.1 | 2.72 | 0.62 | 11.27 | 124.6-124.6 | 119-119.6 | 124.9-125.2 | *Cis* |
| chr7 | CFD007 | 7 | 56.5 | 5.14 | 0.81 | 20.33 | 129.9-129.9 | 128.4-129.4 | 130-130 | *Cis* |
| chr7 | CFD009 | 6 | 58.1 | 2.6 | -0.29 | 12.44 | 148.2-185.6 | 138.5-139.5 | 151.2-151.9 | *Trans* |
| chr7 | CFD010 | 1 | 77.4 | 4.61 | -0.36 | 15.82 | 196.2-197.3 | 194.7-194.9 | 198.2-198.2 | *Trans* |
| chr7 | CFD010 | 7 | 113.4 | 3.32 | 0.3 | 11.04 | 171.8-171.8 | 171-171 | 172.9-172.9 | *Cis* |
| chr7 | CFD010 | 7 | 119.8 | 2.83 | 0.28 | 9.52 | 172.9-172.9 | 172.9-172.9 | 173.2-173.2 | *Cis* |
| chr7 | CFD011 | 7 | 33 | 3.33 | 0.37 | 12.52 | 92.9-101.7 | 83-83 | 106.7-107.4 | *Cis* |
| chr7 | CFF001 | 6 | 108.1 | 3.13 | 0.38 | 5.04 | 163-164 | 162.2-162.5 | 165.5-165.5 | *Trans* |
| chr7 | CFF001 | 7 | 58.9 | 2.93 | -0.57 | 11.01 | 107.4-107.7 | 105.9-106.6 | 121-121.3 | *Cis* |
| chr7 | CFF001 | 7 | 67.9 | 5.77 | -1.01 | 19.92 | 125.9-126.5 | 123.6-125.4 | 127.3-127.9 | *Cis* |
| chr7 | CFF002 | 2 | 0 | 3.4 | 0.32 | 9.53 | 0-1.3 | 0-1.3 | 4-4.2 | *Trans* |
| chr7 | CFF002 | 3 | 155.2 | 2.66 | 0.29 | 7.36 | 219.8-220.7 | 217.7-218.8 | 223.7-224.5 | *Trans* |
| chr7 | CFF003 | 7 | 66 | 8.01 | 0.97 | 27.04 | 96.8-96.8 | 86.8-89.1 | 99.5-100.2 | *Cis* |
| chr7 | CFF003 | 7 | 76.5 | 4.79 | -0.74 | 15.54 | 117.7-117.7 | 118.6-121 | 121.2-121.2 | *Cis* |
| chr7 | CFF004 | 4 | 58.3 | 6.84 | 0.63 | 32.17 | 121.6-125 | 100.4-110.5 | 138-146.1 | *Trans* |
| chr7 | CFF004 | 5 | 70.2 | 3.62 | 0.45 | 14.65 | 71.9-72.4 | 64.8-70 | 88-93.3 | *Trans* |
| chr7 | CFF004 | 5 | 79.2 | 2.63 | 0.39 | 11.57 | 167.3-167.3 | 151.1-163.3 | 167.3-167.3 | *Trans* |
| chr7 | CFF006 | 7 | 62.4 | 4.78 | 2.65 | 27.88 | 94-101.7 | 92.7-92.8 | 104-108.4 | *Cis* |
| chr7 | CFF009 | 8 | 99 | 3.76 | 0.28 | 9.5 | 171.5-171.6 | 171.2-171.5 | 172-172 | *Trans* |
| chr7 | CFF012 | 3 | 18.7 | 2.96 | -0.34 | 8.41 | 4.7-4.7 | 3.7-3.8 | 5.9-6 | *Trans* |
| chr7 | CFF012 | 3 | 116.8 | 2.85 | -0.33 | 8.07 | 212.7-212.7 | 202.8-204.5 | 215.7-215.7 | *Trans* |
| chr7 | CFF013 | 4 | 97.5 | 2.94 | -0.34 | 7.26 | 180.4-180.6 | 178.6-178.6 | 180.7-180.7 | *Trans* |
| chr7 | CFF013 | 4 | 103.1 | 3.06 | -0.33 | 7.04 | 182-182 | 180.7-180.7 | 196.2-196.4 | *Trans* |
| chr7 | CFF013 | 5 | 33.9 | 2.63 | 0.31 | 6.16 | 9.3-10 | 7.7-7.7 | 10.4-11.4 | *Trans* |
| chr7 | CFF013 | 7 | 20.9 | 6.87 | 0.69 | 19.82 | 115.6-115.6 | 110.6-110.6 | 115.7-116 | *Cis* |
| chr7 | CFF015 | 3 | 92.7 | 3.59 | 0.31 | 10.34 | 170.3-170.5 | 166.4-166.4 | 170.9-171.9 | *Trans* |
| chr7 | CFF015 | 6 | 106.8 | 2.84 | 0.28 | 8.26 | 163.2-163.2 | 162-162 | 163.6-164 | *Trans* |
| chr7 | CFF015 | 6 | 113.3 | 3.51 | 0.31 | 10.11 | 164.7-164.7 | 163.6-164 | 166.5-166.6 | *Trans* |
| chr7 | CFF015 | 8 | 1.8 | 2.79 | -0.27 | 7.8 | 16-16 | 15.6-16 | 17.2-17.2 | *Trans* |
| chr8 | CFD002 | 7 | 81.3 | 3.46 | 0.28 | 12.17 | 162-162 | 160.2-161.3 | 162.1-162.3 | *Trans* |
| chr8 | CFD002 | 7 | 88.5 | 4.12 | 0.31 | 14.19 | 165.5-165.5 | 162.8-163.9 | 167.2-168 | *Trans* |
| chr8 | CFD002 | 7 | 100.7 | 2.96 | 0.27 | 10.69 | 171.7-172.2 | 171.2-171.5 | 172.7-172.8 | *Trans* |
| chr8 | CFD002 | 10 | 18.8 | 3.18 | 0.27 | 10.74 | 5.6-5.7 | 4.9-5 | 6.1-6.1 | *Trans* |
| chr8 | CFD004 | 8 | 2.9 | 3.98 | -0.3 | 13.31 | 2.1-3.2 | 0-1.1 | 5月5日 | *Cis* |
| chr8 | CFD004 | 8 | 11.9 | 3.33 | -0.28 | 11.27 | 8.1-8.4 | 5.2-5.2 | 8.9-9.9 | *Cis* |
| chr8 | CFD005 | 1 | 78.6 | 3.39 | -0.36 | 15.3 | 197.3-197.3 | 196.8-196.8 | 199.7-202.2 | *Trans* |
| chr8 | CFD007 | 8 | 65 | 3.43 | -0.33 | 11.59 | 123-123 | 111-111 | 131.4-131.9 | *Cis* |
| chr8 | CFD009 | 9 | 66.2 | 2.6 | -0.58 | 13.15 | 125.6-132 | 111.3-111.3 | 142-142 | *Trans* |
| chr8 | CFD009 | 9 | 89.9 | 3.65 | 0.79 | 28.49 | 149.8-149.9 | 149.8-149.9 | 150.2-150.7 | *Trans* |
| chr8 | CFD010 | 10 | 1 | 4.3 | 0.38 | 15.07 | 1.9-2.4 | 1.9-2.4 | 4.9-4.9 | *Trans* |
| chr8 | CFD011 | 1 | 61 | 3.1 | 0.6 | 12.54 | 215.9-217.5 | 214.3-214.9 | 224.4-224.4 | *Trans* |
| chr8 | CFD012 | 3 | 139.6 | 3.21 | 0.37 | 12.21 | 214.6-215 | 213.8-213.8 | 217.7-218.8 | *Trans* |
| chr8 | CFF001 | 1 | 216.5 | 2.89 | 0.37 | 9.85 | 292.1-292.5 | 290.2-291.9 | 292.7-293.6 | *Trans* |
| chr8 | CFF001 | 1 | 222.7 | 3.79 | 0.42 | 12.72 | 295.4-295.6 | 295.2-295.2 | 295.9-295.9 | *Trans* |
| chr8 | CFF001 | 2 | 2.1 | 3.73 | -0.42 | 12.44 | 2.1-2.1 | 1.4-1.6 | 3.5-3.5 | *Trans* |
| chr8 | CFF001 | 3 | 127.3 | 2.64 | -0.36 | 8.59 | 215.5-215.5 | 214.8-214.8 | 217.6-217.8 | *Trans* |
| chr8 | CFF002 | 1 | 177.8 | 3.12 | 0.46 | 7.27 | 241-242.6 | 234.8-234.8 | 242.9-243.5 | *Trans* |
| chr8 | CFF003 | 6 | 72.9 | 2.92 | -0.33 | 9.18 | 105.3-105.3 | 103.4-105.1 | 105.5-105.5 | *Trans* |
| chr8 | CFF003 | 6 | 77.9 | 2.91 | -0.33 | 9.14 | 107.8-107.8 | 106.9-106.9 | 107.8-108.1 | *Trans* |
| chr8 | CFF003 | 9 | 122.2 | 2.79 | 0.32 | 8.73 | 145.5-145.8 | 142.9-144 | 146.9-147.5 | *Trans* |
| chr8 | CFF004 | 3 | 69.7 | 3.78 | 0.49 | 17.55 | 159.7-159.7 | 156.6-159 | 159.7-159.8 | *Trans* |
| chr8 | CFF004 | 4 | 83.7 | 4.31 | 0.47 | 22.48 | 160.3-163.4 | 157-157 | 160.3-163.4 | *Trans* |
| chr8 | CFF004 | 4 | 89.5 | 4.33 | 0.46 | 20.51 | 166.9-166.9 | 160.3-163.4 | 167.2-169.2 | *Trans* |
| chr8 | CFF004 | 6 | 97.9 | 4.75 | 0.49 | 23.84 | 154.2-154.2 | 153.2-153.7 | 154.3-155.2 | *Trans* |
| chr8 | CFF004 | 6 | 103.9 | 4.06 | 0.45 | 20.95 | 155.2-156.5 | 154.3-155.2 | 157.3-158 | *Trans* |
| chr8 | CFF006 | 2 | 48.9 | 6.28 | -1.59 | 29.01 | 16.4-16.7 | 13.8-16 | 16.8-17.3 | *Trans* |
| chr8 | CFF006 | 2 | 57.5 | 6.23 | -1.56 | 28.83 | 20.5-20.8 | 19.1-19.1 | 22.9-28 | *Trans* |
| chr8 | CFF006 | 10 | 84.2 | 2.87 | -0.91 | 11.03 | 134.6-136.7 | 134.4-134.5 | 138.2-138.2 | *Trans* |
| chr8 | CFF007 | 9 | 14.4 | 3.49 | -0.34 | 9.84 | 8.9-9.1 | 8.6-8.6 | 10-10.3 | *Trans* |
| chr8 | CFF009 | 9 | 54.5 | 2.69 | 0.26 | 7.62 | 109.8-109.8 | 107.4-107.4 | 113.8-113.8 | *Trans* |
| chr8 | CFF010 | 1 | 97.2 | 3.49 | 0.32 | 11.78 | 96.1-103.3 | 88-93.1 | 107.2-171.7 | *Trans* |
| chr8 | CFF013 | 8 | 67.2 | 5.36 | 0.57 | 15.76 | 104.3-104.3 | 103.9-103.9 | 104.6-104.8 | *Cis* |
| chr8 | CFF013 | 8 | 77.3 | 11.26 | 0.99 | 30.46 | 112.5-116 | 111.3-111.8 | 112.5-116 | *Cis* |
| chr8 | CFF015 | 8 | 29.2 | 3.48 | 0.36 | 6.66 | 72-88.7 | 58.5-66 | 89-89 | *Cis* |
| chr8 | CFF015 | 8 | 47.2 | 2.94 | 0.58 | 5.69 | 114-115.3 | 111.3-113.7 | 115.7-117.8 | *Cis* |
| chr8 | CFF015 | 8 | 63 | 15.26 | -1.06 | 39.33 | 118.2-118.3 | 118.2-118.2 | 118.3-120 | *Cis* |
| chr8 | CFF015 | 8 | 81.5 | 7.68 | 0.79 | 18.71 | 130.7-130.7 | 119-119.3 | 134.1-134.1 | *Cis* |
| chr9 | CFD002 | 5 | 65.2 | 3.36 | -0.38 | 11.83 | 31.9-48.7 | 31.9-48.7 | 68.4-75.1 | *Trans* |
| chr9 | CFD002 | 5 | 76.1 | 5.09 | -0.46 | 16.87 | 163.4-164.4 | 158.7-159.3 | 172.3-173.8 | *Trans* |
| chr9 | CFD002 | 5 | 86.9 | 4.49 | -0.42 | 15.19 | 175.7-180.4 | 174.3-174.6 | 180.9-183.7 | *Trans* |
| chr9 | CFD002 | 10 | 89.8 | 2.62 | 0.33 | 8.08 | 147-147.2 | 146.5-146.5 | 148.6-148.6 | *Trans* |
| chr9 | CFD003 | 6 | 15.1 | 2.96 | 0.37 | 10.18 | 25.3-28.1 | 19.4-23.8 | 55.1-56.8 | *Trans* |
| chr9 | CFD003 | 6 | 22.1 | 3.57 | 0.4 | 12.1 | 86.3-89 | 80-85.2 | 94.8-95.3 | *Trans* |
| chr9 | CFD004 | 9 | 0 | 3.86 | 0.38 | 12.67 | 0.3-1.3 | 0.3-1.3 | 5.2-5.3 | *Cis* |
| chr9 | CFD005 | 4 | 83.2 | 3 | 0.38 | 12.28 | 188-188 | 185.4-185.7 | 191.6-192.4 | *Trans* |
| chr9 | CFD005 | 4 | 90.1 | 3.49 | 0.38 | 14.08 | 201.5-202.8 | 191.4-191.4 | 206.1-211.2 | *Trans* |
| chr9 | CFD005 | 9 | 20 | 5.11 | -0.51 | 21.72 | 11.1-11.1 | 10月10日 | 12-12.7 | *Cis* |
| chr9 | CFD006 | 5 | 80.6 | 2.91 | -0.71 | 8.88 | 167.9-167.9 | 141.1-158.5 | 164-167.8 | *Trans* |
| chr9 | CFD006 | 9 | 15.8 | 3.98 | -0.57 | 12.4 | 6-6.1 | 5.2-5.2 | 7月7日 | *Cis* |
| chr9 | CFD007 | 4 | 27.9 | 3.03 | 0.53 | 11.3 | 10.2-10.2 | 10.1-10.1 | 10.8-10.8 | *Trans* |
| chr9 | CFD009 | 2 | 123.6 | 3.62 | -0.37 | 17.21 | 233.1-233.1 | 222.6-222.9 | 236.8-237 | *Trans* |
| chr9 | CFD011 | 6 | 18.2 | 2.65 | -0.27 | 8.62 | 65.3-78.7 | 60.1-64.2 | 75.4-84.7 | *Trans* |
| chr9 | CFD011 | 9 | 55 | 3.65 | 0.32 | 12.65 | 56.9-76.1 | 35.9-46.9 | 76.8-87.3 | *Cis* |
| chr9 | CFD011 | 9 | 68.5 | 4.32 | 0.39 | 21.51 | 89.3-89.3 | 89.3-89.3 | 126.8-126.8 | *Cis* |
| chr9 | CFF003 | 4 | 58.4 | 3.78 | 0.37 | 11.74 | 143.1-143.4 | 131.9-140 | 146.3-146.3 | *Trans* |
| chr9 | CFF004 | 1 | 30.6 | 2.62 | -0.34 | 12.5 | 19.3-19.3 | 14-16.8 | 34.3-35 | *Trans* |
| chr9 | CFF006 | 4 | 99.8 | 2.82 | 0.81 | 14.02 | 172.1-172.2 | 170-171.6 | 175.3-177.2 | *Trans* |
| chr9 | CFF006 | 6 | 23.5 | 4.43 | 1.1 | 23.19 | 91.6-91.6 | 85.2-85.2 | 92.3-92.3 | *Trans* |
| chr9 | CFF006 | 7 | 118.5 | 3.19 | 0.84 | 16.17 | 159-159.7 | 154.1-159 | 161.9-161.9 | *Trans* |
| chr9 | CFF007 | 9 | 126.1 | 4.18 | -0.45 | 8.62 | 154.4-155.6 | 154.1-154.3 | 154.6-154.7 | *Cis* |
| chr9 | CFF007 | 9 | 158.8 | 6.41 | 0.54 | 13.74 | 155-155.7 | 154.6-154.7 | 155.4-155.7 | *Cis* |
| chr9 | CFF008 | 7 | 54.8 | 4.05 | -0.48 | 18.25 | 100.2-103.3 | 86.5-90.7 | 107.9-116.4 | *Trans* |
| chr9 | CFF009 | 9 | 36.6 | 3.06 | 0.29 | 7.9 | 14.5-14.5 | 13.4-13.5 | 18.6-19.3 | *Cis* |
| chr9 | CFF009 | 9 | 97.1 | 3.28 | 0.37 | 9.14 | 148.1-148.4 | 147.8-147.8 | 149.4-149.4 | *Cis* |
| chr9 | CFF009 | 9 | 108.6 | 5.39 | 0.41 | 14.5 | 151.3-151.5 | 149.4-149.4 | 154.4-154.6 | *Cis* |
| chr9 | CFF010 | 3 | 51.1 | 3.09 | -0.29 | 9.47 | 12.2-215.7 | 11.4-12.1 | 13.3-22.3 | *Trans* |
| chr9 | CFF010 | 3 | 64.1 | 3.25 | -0.29 | 9.91 | 113-113.2 | 92.4-92.4 | 116-121 | *Trans* |
| chr10 | CFD001 | 7 | 57.7 | 2.95 | -0.29 | 11.66 | 99.2-99.3 | 95.8-97.2 | 100.9-103.9 | *Trans* |
| chr10 | CFD001 | 8 | 43.2 | 3.2 | 0.28 | 11.55 | 14.6-14.8 | 12.3-12.6 | 15.1-18.2 | *Trans* |
| chr10 | CFD002 | 7 | 110.4 | 2.66 | 0.24 | 10.15 | 173.3-173.3 | 172.8-172.9 | 173.5-173.5 | *Trans* |
| chr10 | CFD002 | 9 | 56 | 3.22 | -0.3 | 13.72 | 124.6-127.5 | 113.2-113.5 | 1321-135.9 | *Trans* |
| chr10 | CFD004 | 2 | 127.9 | 2.86 | 0.26 | 8.84 | 217.3-217.3 | 213.2-215.6 | 222.1-225 | *Trans* |
| chr10 | CFD006 | 10 | 17.3 | 3.93 | -0.42 | 12.53 | 4-4.7 | 2.4-2.5 | 4.9-4.9 | *Cis* |
| chr10 | CFD007 | 9 | 89.3 | 3.47 | -0.3 | 11.95 | 147.6-147.6 | 146.8-147.1 | 148.1-148.2 | *Trans* |
| chr10 | CFD009 | 5 | 105.5 | 2.79 | -0.49 | 14.74 | 206.3-207.5 | 205.3-205.3 | 209-209.7 | *Trans* |
| chr10 | CFD010 | 10 | 43.4 | 4.06 | 0.36 | 15.31 | 108.5-117.1 | 80-80 | 117.6-117.8 | *Cis* |
| chr10 | CFD011 | 8 | 65.7 | 2.96 | 0.62 | 8.71 | 123.8-123.8 | 123.8-123.9 | 124.9-125.2 | *Trans* |
| chr10 | CFD012 | 1 | 146.2 | 3.09 | -0.29 | 9.68 | 230.9-235.5 | 230-230 | 237.1-237.9 | *Trans* |
| chr10 | CFD012 | 1 | 151.2 | 2.86 | -0.28 | 8.96 | 240.5-240.5 | 237.1-237.9 | 252.1-253.8 | *Trans* |
| chr10 | CFD012 | 3 | 61 | 3.98 | -0.98 | 12.65 | 126.2-128.1 | 100.3-112.2 | 126.2-128.1 | *Trans* |
| chr10 | CFD012 | 3 | 83.3 | 5.48 | 1.11 | 20.23 | 128.1-128.1 | 100.8-100.9 | 128.1-128.1 | *Trans* |
| chr10 | CFF001 | 1 | 5.1 | 3.16 | -0.26 | 12.31 | 3.2-3.4 | 2.7-2.9 | 3.7-4 | *Trans* |
| chr10 | CFF001 | 9 | 85.4 | 2.93 | 0.25 | 10.04 | 136-136.6 | 128.4-133.2 | 136.9-137.4 | *Trans* |
| chr10 | CFF003 | 5 | 110.8 | 3.51 | 0.31 | 10.61 | 167.6-168.4 | 160.1-163.1 | 169.9-170.5 | *Trans* |
| chr10 | CFF003 | 9 | 78.5 | 3.97 | -0.33 | 12.06 | 108-109.3 | 105.2-105.4 | 111.1-112.2 | *Trans* |
| chr10 | CFF006 | 5 | 102 | 4.7 | 0.57 | 27.29 | 141.2-141.2 | 126.8-132.7 | 155.2-158.8 | *Trans* |
| chr10 | CFF007 | 2 | 188 | 4 | 0.22 | 11.26 | 233.1-233.1 | 232.4-232.4 | 233.7-234.7 | *Trans* |
| chr10 | CFF007 | 5 | 4 | 3.82 | -0.25 | 14.24 | 1.8-1.8 | 1.8-1.8 | 4.6-4.9 | *Trans* |
| chr10 | CFF007 | 7 | 22.7 | 3.89 | -0.2 | 10.15 | 3.2-3.7 | 3月3日 | 5-5.1 | *Trans* |
| chr10 | CFF007 | 9 | 105.9 | 2.68 | -0.17 | 6.88 | 151.2-151.3 | 150-150 | 151.3-151.3 | *Trans* |
| chr10 | CFF008 | 10 | 66.2 | 3.49 | 0.3 | 13.13 | 113.5-113.9 | 75.9-76.5 | 117.8-117.9 | *Cis* |
| chr10 | CFF008 | 10 | 75.3 | 2.64 | 0.25 | 10.2 | 124.3-126.6 | 121.8-121.8 | 130.3-132.1 | *Cis* |
| chr10 | CFF010 | 4 | 142.7 | 3.85 | -0.27 | 12.84 | 239-239.1 | 238-238.2 | 239.7-239.8 | *Trans* |
| chr10 | CFF013 | 9 | 37.8 | 3.01 | 0.27 | 9.51 | 14.5-14.6 | 12.1-12.3 | 15.6-15.6 | *Trans* |
| chr10 | CFF015 | 2 | 165.6 | 3.39 | -0.42 | 11.37 | 213.2-213.3 | 198.8-209.4 | 215.1-215.3 | *Trans* |
| chr10 | CFF015 | 8 | 45.4 | 2.66 | 0.25 | 8.3 | 111.3-113.7 | 103.9-104.8 | 115.7-117.8 | *Trans* |
| Total | CFD001 | 3 | 65.1 | 2.79 | 1.85 | 12.51 | 122.1-127.5 | 107.7-122.1 | 127.9-134.8 | Total |
| Total | CFD001 | 3 | 71.1 | 3.85 | 2.17 | 16.82 | 154-154.6 | 152-152 | 156-156 | Total |
| Total | CFD001 | 3 | 81.5 | 2.76 | 1.94 | 12.73 | 161.7-162.3 | 159.9-161.6 | 161.7-162.3 | Total |
| Total | CFD001 | 7 | 53.1 | 2.66 | 1.56 | 9.44 | 9.3-9.5 | 9.2-9.2 | 9.9-10.5 | Total |
| Total | CFD001 | 7 | 71.3 | 3.83 | 1.98 | 14.21 | 118.6-118.6 | 109.2-117.4 | 121-121.3 | Total |
| Total | CFD002 | 4 | 23.3 | 2.83 | -1.6 | 12.58 | 14-14 | 11-11.1 | 14.5-14.6 | Total |
| Total | CFD002 | 4 | 39.8 | 3.31 | -1.82 | 14.67 | 153.5-154.2 | 153.3-153.5 | 154.4-157 | Total |
| Total | CFD003 | 3 | 98.2 | 4.29 | 1.53 | 14.02 | 195.3-196.2 | 194.6-194.8 | 199.7-201.2 | Total |
| Total | CFD003 | 6 | 40.1 | 3.6 | 1.39 | 11.63 | 102.4-103.9 | 96.7-97.3 | 103.3-104.1 | Total |
| Total | CFD003 | 6 | 46.1 | 3.86 | 1.45 | 13.13 | 105.4-106.5 | 103.3-104.1 | 107.9-108.1 | Total |
| Total | CFD004 | 3 | 44.8 | 6.43 | -3.29 | 19.88 | 33.1-48 | 48-48 | 48.4-52.2 | Total |
| Total | CFD004 | 3 | 60.6 | 8.81 | 4.02 | 29.5 | 154.7-155 | 154.5-154.7 | 155.8-156 | Total |
| Total | CFD005 | 1 | 22.7 | 3.22 | 1.22 | 12.37 | 15.7-16 | 12.5-15.1 | 19.7-21.7 | Total |
| Total | CFD005 | 3 | 66.2 | 3.37 | -1.28 | 13.05 | 183.9-184 | 176-176.8 | 184.2-184.8 | Total |
| Total | CFD005 | 3 | 71.5 | 3.58 | -1.3 | 13.75 | 187-187.4 | 186.2-187 | 188.5-189.3 | Total |
| Total | CFD006 | 3 | 40.5 | 10.19 | 6.66 | 35.62 | 12.6-17.9 | 9-9.1 | 12.6-17.9 | Total |
| Total | CFD006 | 3 | 47.5 | 4.94 | 3.5 | 18.98 | 21.6-22.1 | 19.8-25.1 | 28.1-28.6 | Total |
| Total | CFD006 | 6 | 96.6 | 3.14 | 1.93 | 7.39 | 160.6-160.6 | 155.5-155.5 | 158.8-159.2 | Total |
| Total | CFD007 | 1 | 158.5 | 3.24 | 1.24 | 10.75 | 254.5-254.5 | 253.3-253.9 | 260.4-261.4 | Total |
| Total | CFD007 | 1 | 164.1 | 2.67 | 1.18 | 9.13 | 263-264.2 | 260.4-261.4 | 273.7-273.7 | Total |
| Total | CFD010 | 5 | 47.2 | 2.62 | -1.48 | 9.24 | 11.4-11.7 | 10.2-10.3 | 16.7-16.7 | Total |
| Total | CFD011 | 1 | 2.5 | 3.11 | 1.45 | 10.63 | 39.9-39.9 | 37.3-39.1 | 40.5-41.2 | Total |
| Total | CFF001 | 1 | 226.8 | 4.91 | 1.63 | 14.92 | 296.9-297.8 | 295.4-295.6 | 298.3-300.8 | Total |
| Total | CFF001 | 3 | 61.9 | 3.64 | -1.62 | 10.94 | 137.2-140.2 | 137.2-137.2 | 151-151.4 | Total |
| Total | CFF003 | 5 | 32.9 | 3.04 | 1.7 | 11.44 | 10.2-11.3 | 5-5.9 | 12.3-12.4 | Total |
| Total | CFF006 | 2 | 78.6 | 3.49 | -2.71 | 15.57 | 41.9-42.4 | 34.4-36.9 | 44.3-50 | Total |
| Total | CFF006 | 7 | 120.6 | 5.86 | 3.63 | 29.66 | 159.9-160.2 | 154.1-159.8 | 160.6-161.7 | Total |
| Total | CFF006 | 8 | 125.6 | 3.82 | 3.64 | 17.15 | 169.7-169.7 | 168.5-168.9 | 171.8-172 | Total |
| Total | CFF006 | 9 | 6.6 | 3.51 | 2.53 | 15.39 | 5.3-5.5 | 1.3-2.2 | 7.3-8 | Total |
| Total | CFF007 | 5 | 66.1 | 2.62 | 0.98 | 7.47 | 27.8-30.8 | 17.3-18.6 | 31.9-32.8 | Total |
| Total | CFF007 | 6 | 41 | 3.37 | 1.81 | 9.75 | 106.2-110.7 | 105.6-105.9 | 107.8-111 | Total |
| Total | CFF007 | 6 | 79.5 | 2.77 | -1.04 | 7.91 | 154.2-154.8 | 153.7-153.7 | 158.7-158.7 | Total |
| Total | CFF008 | 7 | 46.4 | 3.85 | -1.56 | 15.9 | 7.8-8.2 | 9.3-9.7 | 10.1-10.5 | Total |
| Total | CFF009 | 1 | 139.6 | 2.75 | -1.19 | 7.38 | 211.5-211.5 | 211.3-211.5 | 213.3-214.7 | Total |
| Total | CFF010 | 8 | 123.1 | 3.14 | -1.12 | 10.03 | 172.2-172.2 | 171.9-172 | 172.9-172.9 | Total |
| Total | CFF012 | 4 | 150.8 | 3.31 | 1.9 | 10.55 | 234.8-234.8 | 233.6-233.8 | 234.9-234.9 | Total |
| Total | CFF013 | 3 | 34.1 | 3.12 | -1.25 | 9.57 | 10.5-11 | 9.3-9.3 | 11.2-12.1 | Total |
| Total | CFF015 | 3 | 3.1 | 2.94 | -0.99 | 8.75 | 2.4-2.5 | 1.6-1.7 | 3.7-4.1 | Total |
| Total | CFF015 | 4 | 119.3 | 3.01 | -1.86 | 10.16 | 229.2-231.5 | 227.2-227.5 | 229.2-231.5 | Total |
| Total | CFF015 | 4 | 132.7 | 2.69 | 1.57 | 8.06 | 235.3-235.3 | 235.2-235.3 | 236.1-236.2 | Total |

**Supplementary** **Table 3.** Summary of recombination QTL mapping for GREminus

| Pop | Chr | Peak_gen (cM) | LOD | Additive | *R2* (%) | Peak_bin (Mb) | Left_bin (Mb) | Right_bin (Mb) | Type^a^ |
| --- | --- | --- | --- | --- | --- | --- | --- | --- | --- |
| B73/BY804 | 5 | 146.5 | 5.3 | 3.49 | 10.8 | 204.5-204.5 | 204.1-204.3 | 205.2-205.2 | Total |
| B73/BY804 | 5 | 164 | 2.7 | -2.42 | 5.2 | 209.9-209.9 | 208.3-208.4 | 211.3-211.3 | New |
| YU87-1/BK | 4 | 124.6 | 5.6 | 3.49 | 11.8 | 181.4-181.4 | 180.2-180.2 | 182.1-182.7 | Total |
| YU87-1/BK | 4 | 136.5 | 6.3 | 3.67 | 13.1 | 189.2-189.2 | 187.4-188.3 | 190-190 | Total |
| YU87-1/BK | 4 | 146.2 | 2.9 | 2.54 | 6.3 | 212.3-216.4 | 198.4-199.2 | 225.7-226.3 | New |
| YU87-1/BK | 9 | 75.2 | 2.9 | -2.72 | 7.2 | 122.8-122.8 | 118.9-120.2 | 125.2-125.2 | New |
| DAN340/K22 | 2 | 95.3 | 4.8 | 2.69 | 8.7 | 177.8-177.8 | 170.4-176.5 | 181.2-181.2 | Total |
| DAN340/K22 | 3 | 66.5 | 4.3 | -2.48 | 7.6 | 134.9-134.9 | 121.8-122.1 | 143.8-143.8 | Total |
| DAN340/K22 | 3 | 77.1 | 2.6 | 2.61 | 5.2 | 161.7-162 | 158.5-159.7 | 162.4-162.4 | New |
| DAN340/K22 | 3 | 180.6 | 3.1 | -2.07 | 5.4 | 230.4-230.5 | 229.3-229.3 | 230.5-232 | New |
| DAN340/K22 | 7 | 14.5 | 2.6 | -1.99 | 5 | 5.1-5.1 | 0.8-0.8 | 6.6-6.6 | Total |
| KUI3/SC55 | 4 | 123.6 | 3.6 | -2.26 | 7.2 | 186.3-186.6 | 183-184 | 188-188 | Total |
| KUI3/SC55 | 4 | 163.2 | 3.2 | 2.14 | 6.5 | 236.9-236.9 | 236.5-236.5 | 237.5-237.5 | Total |
| KUI3/B77 | 1 | 263 | 2.6 | -2.12 | 6 | 290.5-290.5 | 290.2-290.2 | 292.3-292.5 | New |
| KUI3/B77 | 1 | 278.3 | 2.6 | 2.22 | 5.9 | 295.5-295.5 | 295.4-295.4 | 296.9-297.8 | New |
| KUI3/B77 | 1 | 286.7 | 4 | 2.53 | 9 | 299.2-299.2 | 298-298 | 299.4-299.4 | New |
| KUI3/B77 | 4 | 103.5 | 3.2 | 2.04 | 7 | 177.2-177.2 | 175.3-175.3 | 177.6-177.6 | New |
| ZHENG58/SK | 3 | 96.2 | 3.8 | -2.6 | 7 | 153.8-155.7 | 150.3-151.3 | 156.5-156.5 | Total |
| ZHENG58/SK | 4 | 67.6 | 2.7 | 2.18 | 4.8 | 29.8-30.8 | 28.1-29.2 | 31.7-31.7 | Total |
| ZONG3/YU87-1 | 3 | 2.3 | 3.5 | -3.39 | 6.2 | 1.5-1.5 | 1.3-1.3 | 1.7-1.8 | Total |
| DE3/BY815 | 4 | 95.2 | 2.9 | 2.56 | 5.3 | 162.3-162.3 | 161.1-161.1 | 163.9-163.9 | New |
| DE3/BY815 | 5 | 64.6 | 3.1 | -2.64 | 5.8 | 16.2-16.2 | 14.6-14.7 | 19.5-19.5 | Total |
| K22/BY815 | 1 | 104.6 | 3.6 | 2.8 | 7.1 | 81.6-81.6 | 72.8-74.2 | 85-88.3 | Total |
| K22/BY815 | 6 | 26.5 | 4.1 | 3.57 | 7.7 | 79.5-79.6 | 75-75.3 | 89.4-89.4 | New |
| K22/BY815 | 6 | 37.4 | 3.7 | -3.53 | 7.1 | 98-98 | 97.6-97.9 | 105.2-105.6 | New |
| a,New is new QTL which was not detected for total recombination events, Total is the QTL detected for total recombination events. | | | | | | | | | |

**Supplementary** **Table 4.** Summary of mapping recombination hots pot QTLs

| Pop | Trait^a^ | Chr | Pos | LOD | Left (cM) | Right (cM) | Left (Mb) | Right (Mb) | Additive | *R2* (%) | Type |
| --- | --- | --- | --- | --- | --- | --- | --- | --- | --- | --- | --- |
| B73/BY804 | 2_2_4 | 2 | 19.4 | 4.68 | 14.5 | 21.4 | 3.4 | 4.1 | 0.112 | 3.86 | Hotspot |
| B73/BY804 | 2_3_5 | 9 | 14.8 | 4.3 | 9.7 | 18 | 5.1 | 8.1 | 0.146 | 7.53 | No hotspot |
| B73/BY804 | 3_2_4 | 2 | 198.7 | 3.1 | 196.5 | 204.7 | 234.3 | 235.9 | 0.122 | 4.4 | No hotspot |
| B73/BY804 | 3_2_4 | 7 | 0.3 | 3.02 | 0 | 8.2 | 0 | 2.1 | 0.153 | 6.21 | No hotspot |
| B73/BY804 | 3_3_5 | 1 | 59.8 | 3.2 | 58.6 | 65.1 | 25.4 | 31.9 | -0.138 | 6.62 | No hotspot |
| B73/BY804 | 3_3_5 | 3 | 24.3 | 3.33 | 23.3 | 28.3 | 4.7 | 6 | 0.086 | 3.66 | Hotspot |
| B73/BY804 | 3_3_5 | 8 | 10.3 | 5.9 | 8.1 | 16.7 | 2.9 | 4.4 | -0.116 | 5.22 | No hotspot |
| B73/BY804 | 4_237_239 | 1 | 177 | 3.25 | 173.6 | 179.8 | 243.1 | 249.3 | -0.106 | 3.82 | No hotspot |
| B73/BY804 | 4_237_239 | 7 | 49 | 4.31 | 45.3 | 55.1 | 9.9 | 21.2 | -0.101 | 3.47 | No hotspot |
| B73/BY804 | 4_238_240 | 7 | 16.1 | 3.13 | 14.3 | 20.3 | 3.2 | 4.7 | 0.145 | 3.58 | No hotspot |
| B73/BY804 | 4_238_240 | 7 | 47.8 | 8.13 | 45.3 | 52.4 | 9.9 | 15.7 | -0.232 | 7.86 | No hotspot |
| B73/BY804 | 4_238_240 | 7 | 78.6 | 3.17 | 76.4 | 83.4 | 123.6 | 130.3 | 0.148 | 5.4 | No hotspot |
| B73/BY804 | 7_4_6 | 7 | 24.8 | 5.46 | 22 | 25.4 | 4.9 | 5.1 | -0.119 | 2.45 | Hotspot |
| B73/BY804 | 7_4_6 | 9 | 23.6 | 3.99 | 18 | 28.8 | 8.1 | 11.5 | 0.159 | 7.58 | No hotspot |
| B73/BY804 | 8_170_172 | 6 | 70 | 3.16 | 65.8 | 74.5 | 130.9 | 142.3 | 0.137 | 5.49 | No hotspot |
| B73/BY804 | 8_171_173 | 9 | 55.2 | 3.74 | 53.2 | 60.7 | 20.8 | 23.5 | 0.151 | 8.12 | No hotspot |
| B73/BY804 | 10_147_149 | 1 | 76.8 | 4.5 | 75 | 79.6 | 46.1 | 54.2 | -0.126 | 5.73 | No hotspot |
| B73/BY804 | 10_147_149 | 9 | 24.2 | 3.98 | 21.3 | 28.8 | 9.4 | 11.5 | 0.083 | 2.72 | No hotspot |
| K22/BY815 | 3_1_3 | 3 | 0 | 3.79 | 0 | 3.5 | 1.1 | 1.6 | -0.071 | 1.61 | Hotspot |
| K22/BY815 | 3_2_4 | 1 | 167.9 | 3.7 | 165 | 169.1 | 232.3 | 240.5 | 0.165 | 8.44 | No hotspot |
| K22/BY815 | 3_2_4 | 5 | 62.8 | 5.69 | 62.3 | 67 | 33 | 55.4 | 0.102 | 3.31 | No hotspot |
| K22/BY815 | 6_164_166 | 2 | 34.4 | 3.21 | 29 | 39.4 | 8.9 | 11.9 | -0.135 | 6.14 | No hotspot |
| K22/BY815 | 6_164_166 | 5 | 47.5 | 3.83 | 46.2 | 49.4 | 13.4 | 14.6 | -0.094 | 3.29 | No hotspot |
| K22/BY815 | 6_165_167 | 2 | 100.9 | 3.62 | 97.9 | 105.3 | 174.7 | 182.6 | -0.149 | 6.21 | No hotspot |
| K22/BY815 | 7_4_6 | 1 | 54.9 | 4.75 | 53.6 | 57.7 | 19 | 19.9 | 0.123 | 5.46 | No hotspot |
| K22/BY815 | 8_4_6 | 2 | 166.6 | 4.29 | 164.5 | 169 | 232.1 | 232.7 | -0.092 | 4.14 | No hotspot |
| K22/BY815 | 8_171_173 | 1 | 195.2 | 3.52 | 193.7 | 197.1 | 271.8 | 276.3 | 0.072 | 2.15 | No hotspot |
| K22/BY815 | 9_153_155 | 9 | 130.7 | 5.24 | 129.8 | 132.5 | 153.6 | 153.9 | 0.041 | 0.76 | Hotspot |
| K22/BY815 | 10_147_149 | 10 | 116.2 | 5.6 | 115.1 | 117.4 | 147 | 147.3 | 0.1 | 3.23 | Hotspot |
| K22/CI7 | 1_5_7 | 2 | 151.7 | 3.63 | 150.7 | 155.1 | 219.3 | 222.7 | -0.107 | 5.47 | No hotspot |
| K22/CI7 | 3_1_3 | 6 | 44.9 | 3.77 | 43.2 | 48.8 | 95.5 | 97.9 | -0.099 | 5.13 | No hotspot |
| K22/CI7 | 3_1_3 | 8 | 21.6 | 3.84 | 17.5 | 23.8 | 5.2 | 6.4 | 0.13 | 7.6 | No hotspot |
| K22/CI7 | 3_2_4 | 9 | 41.6 | 3.36 | 36.7 | 50.6 | 18.7 | 24.1 | 0.135 | 6.39 | No hotspot |
| K22/CI7 | 5_1_3 | 3 | 145.8 | 4.54 | 144.3 | 152.8 | 209 | 212.4 | -0.149 | 6.86 | No hotspot |
| K22/CI7 | 5_1_3 | 5 | 13.1 | 6.21 | 12.2 | 15.5 | 2.6 | 2.9 | 0.081 | 2.01 | Hotspot |
| K22/CI7 | 9_151_153 | 5 | 167.9 | 5.06 | 162.7 | 169.2 | 212.7 | 213.5 | -0.114 | 4.73 | No hotspot |
| BY815/KUI3 | 3_2_4 | 3 | 175.2 | 4.64 | 173.7 | 177.6 | 217.6 | 218 | 0.168 | 6.94 | Hotspot |
| BY815/KUI3 | 3_2_4 | 4 | 180.2 | 3.2 | 174.6 | 185.5 | 238.3 | 239.5 | -0.119 | 3.71 | No hotspot |
| BY815/KUI3 | 4_2_4 | 5 | 196.2 | 5.67 | 194.1 | 197.8 | 213.1 | 213.7 | 0.144 | 7.76 | No hotspot |
| BY815/KUI3 | 4_238_240 | 1 | 42.2 | 3.28 | 36.3 | 46.3 | 12.3 | 16.3 | 0.116 | 4.38 | No hotspot |
| BY815/KUI3 | 4_238_240 | 3 | 186.5 | 4.01 | 183.5 | 188.7 | 219.8 | 221.5 | 0.104 | 3.45 | No hotspot |
| BY815/KUI3 | 5_212_214 | 4 | 11.9 | 4.6 | 10 | 16.1 | 2.9 | 3.5 | 0.165 | 3.13 | No hotspot |
| BY815/KUI3 | 5_212_214 | 6 | 55.3 | 3.04 | 51.9 | 59.2 | 96.9 | 102.5 | 0.124 | 3.21 | No hotspot |
| BY815/KUI3 | 5_212_214 | 7 | 85.4 | 3.91 | 81.1 | 88.4 | 123.8 | 128.7 | -0.157 | 6.83 | No hotspot |
| BY815/KUI3 | 5_212_214 | 10 | 94.9 | 6.34 | 93.7 | 98.8 | 132.6 | 133.3 | 0.182 | 12.03 | No hotspot |
| BY815/KUI3 | 7_1_3 | 9 | 25.8 | 3.28 | 24.9 | 30.4 | 14.5 | 17.7 | 0.146 | 7.77 | No hotspot |
| BY815/KUI3 | 7_4_6 | 7 | 160.1 | 4.87 | 157.1 | 163.2 | 168.7 | 170.6 | -0.148 | 8.55 | No hotspot |
| BY815/KUI3 | 10_2_4 | 4 | 135.6 | 4.7 | 134.4 | 140.6 | 192.6 | 211.1 | -0.106 | 4.86 | No hotspot |
| KUI3/B77 | 3_2_4 | 1 | 160.7 | 5.25 | 157.7 | 162.6 | 189.4 | 197.4 | 0.151 | 8.59 | No hotspot |
| KUI3/B77 | 10_2_4 | 8 | 106.8 | 4.81 | 104.4 | 109.5 | 160.6 | 162.7 | -0.118 | 8.04 | No hotspot |
| KUI3/B77 | 10_4_6 | 4 | 10.4 | 3.29 | 8.1 | 25.4 | 2.8 | 6 | -0.108 | 4.93 | No hotspot |
| ZONG3/YU87-1 | 3_1_3 | 9 | 101.3 | 3.11 | 99.2 | 104.5 | 99 | 103.9 | 0.19 | 5.51 | No hotspot |
| ZONG3/YU87-1 | 5_1_3 | 8 | 219.7 | 4.09 | 219.2 | 222.8 | 173.5 | 173.8 | 0.148 | 2.8 | No hotspot |
| ZONG3/YU87-1 | 7_1_3 | 7 | 54.1 | 3.47 | 47 | 65.5 | 5.5 | 9.7 | -0.169 | 4.82 | No hotspot |
| ZONG3/YU87-1 | 10_1_3 | 4 | 81.9 | 3.59 | 79.8 | 85.1 | 26.4 | 31.7 | -0.174 | 6.44 | No hotspot |
| ZONG3/YU87-1 | 10_1_3 | 9 | 143.1 | 3.71 | 139.4 | 144 | 138.8 | 140.9 | -0.141 | 5.74 | No hotspot |
| ZONG3/YU87-1 | 10_1_3 | 10 | 133.1 | 3.76 | 128 | 137.1 | 136 | 137.3 | 0.144 | 5.33 | Hotspot |
| ZONG3/YU87-1 | 10_147_149 | 1 | 186.8 | 3.15 | 181.2 | 191.9 | 96.1 | 171.5 | -0.173 | 5.22 | No hotspot |
| ZONG3/YU87-1 | 10_147_149 | 5 | 155 | 4.54 | 153.4 | 165.2 | 167.9 | 171.4 | -0.184 | 6.96 | No hotspot |
| ZONG3/YU87-1 | 10_147_149 | 8 | 32.9 | 5.97 | 30.6 | 35.4 | 5.4 | 6.5 | -0.2 | 9.01 | No hotspot |
| ZHENG58/SK | 2_2_4 | 2 | 208.6 | 3.07 | 202.7 | 212.2 | 234.7 | 236.6 | -0.125 | 7.05 | Hotspot |
| ZHENG58/SK | 2_2_4 | 7 | 90.6 | 4.59 | 89.2 | 92.5 | 146 | 148.2 | -0.09 | 3.29 | No hotspot |
| ZHENG58/SK | 3_2_4 | 2 | 167.7 | 3.08 | 162 | 170.6 | 219.7 | 223.3 | 0.118 | 5.27 | No hotspot |
| ZHENG58/SK | 5_1_3 | 2 | 113.2 | 3.73 | 111.7 | 116.4 | 141.7 | 148.7 | -0.092 | 2.79 | No hotspot |
| ZHENG58/SK | 5_2_4 | 4 | 66 | 3.66 | 65.4 | 67.3 | 22.8 | 29.2 | -0.055 | 1.41 | No hotspot |
| ZHENG58/SK | 8_171_173 | 10 | 67.8 | 3.06 | 62.7 | 69.8 | 134.4 | 137.3 | 0.123 | 4.72 | No hotspot |
| ZHENG58/SK | 9_152_154 | 9 | 129.3 | 13.14 | 125.6 | 132.8 | 152.9 | 153.7 | -0.017 | 0.12 | Hotspot |
| a, the left value is the chromosome of recombination hots regions, the middle value is the start position of recombination hots regions, the right value is the end position of recombination hots regions. | | | | | | | | | | | |

**Supplementary** **Table 5.** Summary of the number of the six types of DNA transposons detected in each chromosome

| Transponson | Enspm | Harbinger | hAT | Helitron | MULE | Stowaway |
| --- | --- | --- | --- | --- | --- | --- |
| chr1 | 3859 | 5662 | 6334 | 4467 | 3140 | 1378 |
| chr2 | 3852 | 4306 | 4552 | 3431 | 2417 | 1033 |
| chr3 | 2609 | 4149 | 4628 | 3186 | 2280 | 980 |
| chr4 | 3970 | 3818 | 4305 | 3249 | 2395 | 910 |
| chr5 | 2743 | 3909 | 4408 | 3171 | 2026 | 945 |
| chr6 | 2454 | 2952 | 3031 | 2513 | 1559 | 657 |
| chr7 | 2404 | 2857 | 3222 | 2353 | 1643 | 740 |
| chr8 | 2360 | 3130 | 3598 | 2610 | 1717 | 777 |
| chr9 | 2175 | 2848 | 2957 | 2260 | 1589 | 688 |
| chr10 | 2413 | 2488 | 2733 | 2068 | 1509 | 573 |
| Total | 28839 | 36119 | 39768 | 29308 | 20275 | 8681 |

**Supplementary** **Table 6.** Correlation coefficient between 21 genomic features or motifs and recombination rate in different sliding genomic window sizes across 12 segregating populations

| Patterns | 10K | 20k | 30k | 40k | 50k | 100k | 200k | 300k | 500k | 1M | 2M |
| --- | --- | --- | --- | --- | --- | --- | --- | --- | --- | --- | --- |
| CCCCACCCC | 0.004 | 0.0145 | 0.0129 | 0.0359 | 0.0311 | 0.0184 | 0.0114 | 0.0183 | 0.0083 | 0.026 | 0.0103 |
| CCCCCCC | 0.0133 | 0.0165 | 0.0195 | 0.0188 | 0.0114 | 0.0234 | 0.0538 | 0.0758 | 0.1224 | 0.2351 | 0.3546 |
| CCNCCNTNNCCNC | 0.0096 | 0.0179 | 0.0216 | 0.029 | 0.0187 | 0.021 | 0.0317 | 0.0462 | 0.0539 | 0.0996 | 0.1552 |
| CCTCCCT | -0.002 | -0.008 | -0.012 | -0.013 | -0.026 | -0.035 | -0.052 | -0.066 | -0.094 | -0.151 | -0.231 |
| CTCTCCC | -0.006 | -0.013 | 0.0031 | -0.009 | 0.000 | 0.0002 | 0.0142 | 0.0271 | 0.0414 | 0.0939 | 0.1842 |
| GCcontent | 0.067 | 0.0376 | 0.0267 | 0.0046 | 0.0049 | 0.0162 | 0.0312 | 0.0495 | 0.0908 | 0.1544 | 0.2522 |
| Copy num | 0.0224 | 0.0436 | 0.0574 | 0.0937 | 0.0874 | 0.1539 | 0.2237 | 0.2976 | 0.3609 | 0.5159 | 0.6461 |
| CpG Island | 0.0563 | 0.0607 | 0.0909 | 0.0834 | 0.0912 | 0.1194 | 0.1705 | 0.2189 | 0.2843 | 0.3951 | 0.4808 |
| Gene Density | 0.0494 | 0.0999 | 0.148 | 0.1676 | 0.1859 | 0.2279 | 0.2895 | 0.3522 | 0.4219 | 0.5443 | 0.6602 |
| Harbinger | 0.0149 | 0.0305 | 0.0683 | 0.0912 | 0.1033 | 0.156 | 0.2232 | 0.2817 | 0.3639 | 0.4999 | 0.6102 |
| hAT | -0.01 | 0.0064 | 0.0284 | 0.0424 | 0.0653 | 0.0941 | 0.1334 | 0.1894 | 0.2581 | 0.3746 | 0.4969 |
| EnSpm | -0.001 | 0.0057 | 0.004 | 0.0128 | 0.0032 | 0.0042 | 0.0028 | -0.015 | -0.031 | -0.045 | -0.075 |
| Helitron | -0.006 | -0.005 | 0.0036 | 0.0208 | 0.0207 | 0.0482 | 0.0924 | 0.1394 | 0.1885 | 0.2975 | 0.4223 |
| MULE | 0.0013 | -0.002 | 0.0019 | 0.0099 | 0.0133 | 0.0152 | 0.0341 | 0.0444 | 0.0681 | 0.0982 | 0.1793 |
| Stowaway | 0.0158 | 0.013 | 0.0422 | 0.0587 | 0.0786 | 0.0949 | 0.166 | 0.2354 | 0.2987 | 0.4485 | 0.5855 |
| Gypsy | -0.044 | -0.063 | -0.081 | -0.1000 | -0.108 | -0.159 | -0.237 | -0.293 | -0.366 | -0.474 | -0.552 |
| Copia | -0.007 | -0.047 | -0.061 | -0.069 | -0.063 | -0.041 | 0.0001 | 0.0306 | 0.0927 | 0.2043 | 0.3124 |
| Sine | -0.003 | -0.01 | -0.011 | -0.008 | -0.005 | 0.0091 | 0.0371 | 0.095 | 0.1421 | 0.2344 | 0.3509 |
| LINE | -0.014 | -0.013 | -0.007 | 0.0042 | 0.0119 | 0.0271 | 0.0619 | 0.107 | 0.1616 | 0.2823 | 0.418 |
| Satellite | -0.003 | -0.005 | -0.007 | -0.007 | -0.006 | -0.012 | -0.018 | -0.012 | -0.019 | -0.026 | -0.025 |
| Simple | 0.0038 | 0.0044 | 0.0123 | 0.0041 | 0.0119 | 0.0073 | 0.0285 | 0.0221 | 0.0345 | 0.0692 | 0.0843 |

**Supplementary** **Table 7.** Correlation coefficient between 21 genomic motifs or features and recombination rate in different sliding genomic window sizes in natural populations

| Patterns | 10k | 20k | 30k | 40k | 50k | 100k | 200k | 300k | 500k | 1M | 2M |
| --- | --- | --- | --- | --- | --- | --- | --- | --- | --- | --- | --- |
| CCCCACCCC | 0.0209 | 0.0221 | 0.0175 | 0.0188 | 0.0204 | 0.009 | -0.003 | -0.004 | -0.024 | -0.033 | -0.072 |
| CCCCCCC | 0.0036 | 0.0056 | 0.0081 | 0.0155 | 0.0223 | 0.0526 | 0.1018 | 0.1474 | 0.217 | 0.334 | 0.4746 |
| CCNCCNTNNCCNC | 0.0139 | 0.0126 | 0.0114 | 0.0121 | 0.0156 | 0.016 | 0.0274 | 0.0395 | 0.0597 | 0.1029 | 0.1837 |
| CCTCCCT | -0.014 | -0.024 | -0.033 | -0.036 | -0.043 | -0.064 | -0.088 | -0.103 | -0.135 | -0.191 | -0.254 |
| CTCTCCC | -0.003 | 0.0003 | 0.0033 | 0.0103 | 0.0115 | 0.0347 | 0.0735 | 0.116 | 0.17 | 0.253 | 0.365 |
| GCcontent | -0.007 | -0.018 | -0.015 | -0.007 | 6.9248 | 0.0385 | 0.1062 | 0.1508 | 0.2204 | 0.3174 | 0.4215 |
| Copy Number | 0.0489 | 0.0863 | 0.1042 | 0.1284 | 0.1451 | 0.2222 | 0.3096 | 0.3724 | 0.4636 | 0.5803 | 0.6893 |
| CpG Island | 0.0938 | 0.1256 | 0.1427 | 0.1491 | 0.1588 | 0.1991 | 0.2674 | 0.3261 | 0.3847 | 0.4836 | 0.5677 |
| Gene Density | 0.1754 | 0.2387 | 0.2666 | 0.2761 | 0.2955 | 0.3302 | 0.3835 | 0.4314 | 0.5011 | 0.6022 | 0.7024 |
| Harbinger | 0.0935 | 0.1539 | 0.1877 | 0.2077 | 0.2292 | 0.2868 | 0.3712 | 0.4256 | 0.5131 | 0.6315 | 0.7229 |
| hAT | 0.0481 | 0.0903 | 0.112 | 0.1272 | 0.1516 | 0.2016 | 0.2644 | 0.3247 | 0.3995 | 0.5209 | 0.638 |
| EnSpm | 0.0115 | 0.0194 | 0.0199 | 0.0218 | 0.0207 | 0.0146 | 0.0136 | 0.0039 | -0.007 | -0.024 | -0.03 |
| Helitron | 0.0122 | 0.0275 | 0.0392 | 0.0481 | 0.0588 | 0.0968 | 0.1454 | 0.2055 | 0.2813 | 0.4034 | 0.5228 |
| MULE | 0.0038 | 0.0154 | 0.0219 | 0.0234 | 0.0266 | 0.0416 | 0.0682 | 0.0932 | 0.1407 | 0.2099 | 0.3069 |
| Stowaway | 0.0487 | 0.0754 | 0.0965 | 0.1088 | 0.1224 | 0.167 | 0.2469 | 0.3017 | 0.3942 | 0.5335 | 0.6731 |
| Gypsy | -0.127 | -0.171 | -0.201 | -0.223 | -0.243 | -0.311 | -0.398 | -0.448 | -0.522 | -0.608 | -0.672 |
| Copia | -0.048 | -0.034 | -0.011 | 0.0048 | 0.0226 | 0.0946 | 0.1827 | 0.2332 | 0.3053 | 0.4166 | 0.5167 |
| SINE | 0.0113 | 0.016 | 0.0209 | 0.0258 | 0.0277 | 0.0432 | 0.0638 | 0.1026 | 0.1578 | 0.2666 | 0.3999 |
| LINE | 0.0078 | 0.023 | 0.0323 | 0.0405 | 0.046 | 0.0639 | 0.111 | 0.1418 | 0.2117 | 0.3358 | 0.4588 |
| Satellite | -0.007 | -0.01 | -0.012 | -0.015 | -0.017 | -0.024 | -0.034 | -0.037 | -0.047 | -0.054 | -0.059 |
| Simple | 0.003 | 0.0023 | 0.0017 | 0.0048 | 0.0031 | 0.0118 | 0.0136 | 0.0157 | 0.038 | 0.064 | 0.0943 |

**Supplementary** **Table 8.** Potential recombination hot regions

| Chr | Start | End | Recombination coefficient | Cutoff |
| --- | --- | --- | --- | --- |
| 1 | 66500000 | 67000000 | 175050.3058 | 95% |
| 1 | 92500000 | 93000000 | 121687.7184 | 95% |
| 1 | 98000000 | 98500000 | 119143.3993 | 95% |
| 1 | 113500000 | 114000000 | 158005.3191 | 95% |
| 1 | 116000000 | 116500000 | 123758.6963 | 95% |
| 1 | 179500000 | 180000000 | 165882.9393 | 95% |
| 1 | 182000000 | 182500000 | 110093.9715 | 95% |
| 1 | 182500000 | 183000000 | 110298.2275 | 95% |
| 1 | 188000000 | 188500000 | 208731.8469 | 95% |
| 1 | 194500000 | 195000000 | 130802.1842 | 95% |
| 1 | 208500000 | 209000000 | 159012.9674 | 95% |
| 2 | 9000000 | 9500000 | 163168.789 | 95% |
| 2 | 16500000 | 17000000 | 135940.1908 | 95% |
| 2 | 20000000 | 20500000 | 154328.1314 | 95% |
| 2 | 22500000 | 23000000 | 126583.4701 | 95% |
| 2 | 44000000 | 44500000 | 247385.294 | 95% |
| 2 | 51000000 | 51500000 | 113676.1632 | 95% |
| 2 | 54500000 | 55000000 | 154452.8696 | 95% |
| 2 | 60500000 | 61000000 | 215122.1509 | 95% |
| 2 | 63500000 | 64000000 | 151451.3545 | 95% |
| 2 | 72000000 | 72500000 | 138241.6014 | 95% |
| 2 | 80000000 | 80500000 | 289385.7523 | 95% |
| 2 | 83500000 | 84000000 | 245762.847 | 95% |
| 2 | 104000000 | 104500000 | 122881.4235 | 95% |
| 2 | 107000000 | 107500000 | 219431.1134 | 95% |
| 2 | 108500000 | 109000000 | 114728.3386 | 95% |
| 2 | 113000000 | 113500000 | 137101.7787 | 95% |
| 2 | 113500000 | 114000000 | 174082.0166 | 95% |
| 2 | 115000000 | 115500000 | 198109.4845 | 95% |
| 2 | 118500000 | 119000000 | 174949.8002 | 95% |
| 2 | 120000000 | 120500000 | 154387.5411 | 95% |
| 2 | 120500000 | 121000000 | 162485.9659 | 95% |
| 2 | 121000000 | 121500000 | 126843.8667 | 95% |
| 2 | 121500000 | 122000000 | 126570.0605 | 95% |
| 2 | 122000000 | 122500000 | 139956.8213 | 95% |
| 2 | 122500000 | 123000000 | 256157.7387 | 95% |
| 2 | 123500000 | 124000000 | 236607.2789 | 95% |
| 2 | 124000000 | 124500000 | 204931.2093 | 95% |
| 2 | 125000000 | 125500000 | 144685.8726 | 95% |
| 2 | 125500000 | 126000000 | 248522.1077 | 95% |
| 2 | 128000000 | 128500000 | 115461.9702 | 95% |
| 2 | 220500000 | 221000000 | 126423.615 | 95% |
| 3 | 32000000 | 32500000 | 122589.8325 | 95% |
| 3 | 127500000 | 128000000 | 139264.1799 | 95% |
| 3 | 129500000 | 130000000 | 290723.6159 | 95% |
| 3 | 147000000 | 147500000 | 256615.1716 | 95% |
| 3 | 155500000 | 156000000 | 122312.8129 | 95% |
| 3 | 158500000 | 159000000 | 133038.6985 | 95% |
| 4 | 2500000 | 3000000 | 157967.2179 | 95% |
| 4 | 14000000 | 14500000 | 152052.3824 | 95% |
| 4 | 81500000 | 82000000 | 151924.9599 | 95% |
| 4 | 110000000 | 110500000 | 110351.9069 | 95% |
| 4 | 114000000 | 114500000 | 121925.6791 | 95% |
| 4 | 130500000 | 131000000 | 156519.826 | 95% |
| 4 | 136500000 | 137000000 | 297238.718 | 95% |
| 4 | 151500000 | 152000000 | 148665.4965 | 95% |
| 4 | 156500000 | 157000000 | 119446.2535 | 95% |
| 4 | 173500000 | 174000000 | 120957.8756 | 95% |
| 5 | 65000000 | 65500000 | 122213.7248 | 95% |
| 5 | 81500000 | 82000000 | 154518.9283 | 95% |
| 5 | 151500000 | 152000000 | 114509.7952 | 95% |
| 5 | 164500000 | 165000000 | 135966.7517 | 95% |
| 5 | 168500000 | 169000000 | 202405.8129 | 95% |
| 5 | 184000000 | 184500000 | 125066.3947 | 95% |
| 5 | 186500000 | 187000000 | 160485.2334 | 95% |
| 5 | 195000000 | 195500000 | 165192.7015 | 95% |
| 5 | 204500000 | 205000000 | 139586.344 | 95% |
| 6 | 500000 | 1000000 | 134836.2723 | 95% |
| 6 | 2000000 | 2500000 | 159592.2848 | 95% |
| 6 | 5000000 | 5500000 | 224312.294 | 95% |
| 6 | 6500000 | 7000000 | 239654.0604 | 95% |
| 6 | 8000000 | 8500000 | 213283.4124 | 95% |
| 6 | 21000000 | 21500000 | 112687.0704 | 95% |
| 6 | 24500000 | 25000000 | 142890.8741 | 95% |
| 6 | 28000000 | 28500000 | 136577.5822 | 95% |
| 6 | 34500000 | 35000000 | 161117.295 | 95% |
| 6 | 35500000 | 36000000 | 186381.1305 | 95% |
| 6 | 36500000 | 37000000 | 255593.3609 | 95% |
| 6 | 57500000 | 58000000 | 159453.2757 | 95% |
| 6 | 58000000 | 58500000 | 180482.0908 | 95% |
| 6 | 59500000 | 60000000 | 169492.2492 | 95% |
| 6 | 61500000 | 62000000 | 128795.092 | 95% |
| 6 | 62000000 | 62500000 | 130176.289 | 95% |
| 6 | 82500000 | 83000000 | 196272.0989 | 95% |
| 6 | 89000000 | 89500000 | 202117.7324 | 95% |
| 6 | 89500000 | 90000000 | 132463.7327 | 95% |
| 6 | 96500000 | 97000000 | 309464.7345 | 95% |
| 6 | 97000000 | 97500000 | 160135.2786 | 95% |
| 6 | 97500000 | 98000000 | 200501.5781 | 95% |
| 6 | 103000000 | 103500000 | 130281.5754 | 95% |
| 6 | 109500000 | 110000000 | 198063.3545 | 95% |
| 7 | 2000000 | 2500000 | 214934.0108 | 95% |
| 7 | 4000000 | 4500000 | 164797.5048 | 95% |
| 7 | 5500000 | 6000000 | 186806.3719 | 95% |
| 7 | 7000000 | 7500000 | 125718.6657 | 95% |
| 7 | 8000000 | 8500000 | 125225.6035 | 95% |
| 7 | 9500000 | 10000000 | 221224.433 | 95% |
| 7 | 10500000 | 11000000 | 124667.9689 | 95% |
| 7 | 14500000 | 15000000 | 168190.5081 | 95% |
| 7 | 17000000 | 17500000 | 159982.038 | 95% |
| 7 | 20500000 | 21000000 | 128080.3735 | 95% |
| 7 | 67500000 | 68000000 | 180168.2812 | 95% |
| 8 | 500000 | 1000000 | 120102.2646 | 95% |
| 8 | 1000000 | 1500000 | 277447.9475 | 95% |
| 8 | 1500000 | 2000000 | 302724.8092 | 95% |
| 8 | 4500000 | 5000000 | 267036.0198 | 95% |
| 8 | 5000000 | 5500000 | 250429.4289 | 95% |
| 8 | 5500000 | 6000000 | 195704.3507 | 95% |
| 8 | 6500000 | 7000000 | 163018.7811 | 95% |
| 8 | 7500000 | 8000000 | 115577.9929 | 95% |
| 8 | 11500000 | 12000000 | 267051.103 | 95% |
| 8 | 12500000 | 13000000 | 218321.2158 | 95% |
| 8 | 14500000 | 15000000 | 195970.4362 | 95% |
| 8 | 16000000 | 16500000 | 248412.6572 | 95% |
| 8 | 16500000 | 17000000 | 264045.7611 | 95% |
| 8 | 18500000 | 19000000 | 269437.6793 | 95% |
| 8 | 19500000 | 20000000 | 237825.6016 | 95% |
| 8 | 23500000 | 24000000 | 163786.3088 | 95% |
| 8 | 25000000 | 25500000 | 170420.0935 | 95% |
| 8 | 73500000 | 74000000 | 117146.9571 | 95% |
| 8 | 74500000 | 75000000 | 200779.4688 | 95% |
| 8 | 161000000 | 161500000 | 179411.8088 | 95% |
| 9 | 1000000 | 1500000 | 194330.2102 | 95% |
| 9 | 3000000 | 3500000 | 165330.1605 | 95% |
| 9 | 3500000 | 4000000 | 187161.4315 | 95% |
| 9 | 4000000 | 4500000 | 191207.618 | 95% |
| 9 | 6000000 | 6500000 | 305419.2637 | 95% |
| 9 | 7000000 | 7500000 | 152672.444 | 95% |
| 9 | 8500000 | 9000000 | 116891.195 | 95% |
| 9 | 9000000 | 9500000 | 234346.6923 | 95% |
| 9 | 10000000 | 10500000 | 182968.9036 | 95% |
| 9 | 10500000 | 11000000 | 138729.851 | 95% |
| 9 | 12000000 | 12500000 | 251518.9979 | 95% |
| 9 | 14000000 | 14500000 | 115272.4016 | 95% |
| 9 | 14500000 | 15000000 | 227951.1742 | 95% |
| 9 | 15000000 | 15500000 | 292417.4636 | 95% |
| 9 | 15500000 | 16000000 | 281885.7993 | 95% |
| 9 | 16500000 | 17000000 | 275766.1077 | 95% |
| 9 | 17000000 | 17500000 | 240962.7914 | 95% |
| 9 | 17500000 | 18000000 | 220089.4067 | 95% |
| 9 | 18000000 | 18500000 | 125300.6515 | 95% |
| 9 | 19500000 | 20000000 | 313523.1748 | 95% |
| 9 | 20000000 | 20500000 | 157171.1922 | 95% |
| 9 | 23000000 | 23500000 | 211663.252 | 95% |
| 9 | 24000000 | 24500000 | 189720.1406 | 95% |
| 9 | 38000000 | 38500000 | 206338.3903 | 95% |
| 10 | 5500000 | 6000000 | 128064.7082 | 95% |
| 10 | 6000000 | 6500000 | 276383.4684 | 95% |
| 10 | 6500000 | 7000000 | 159224.4792 | 95% |
| 10 | 7000000 | 7500000 | 162233.9592 | 95% |
| 10 | 8500000 | 9000000 | 215087.5275 | 95% |
| 10 | 10500000 | 11000000 | 253227.0995 | 95% |
| 10 | 11000000 | 11500000 | 223506.3698 | 95% |
| 10 | 11500000 | 12000000 | 140038.6044 | 95% |
| 10 | 13000000 | 13500000 | 150395.6714 | 95% |
| 10 | 13500000 | 14000000 | 117618.1664 | 95% |
| 10 | 14000000 | 14500000 | 206751.7484 | 95% |
| 10 | 14500000 | 15000000 | 268806.523 | 95% |
| 10 | 17500000 | 18000000 | 183406.2304 | 95% |
| 10 | 86500000 | 87000000 | 120261.6073 | 95% |
| 10 | 87000000 | 87500000 | 261383.8495 | 95% |
| 10 | 134500000 | 135000000 | 129719.3405 | 95% |
| 10 | 136000000 | 136500000 | 114366.7278 | 95% |
| 10 | 142500000 | 143000000 | 138341.1792 | 95% |
| 10 | 148500000 | 149000000 | 129394.911 | 95% |
| 2 | 98500000 | 99000000 | 320142.6801 | 99% |
| 5 | 81000000 | 81500000 | 427890.6711 | 99% |
| 5 | 151000000 | 151500000 | 314273.4107 | 99% |
| 6 | 88500000 | 89000000 | 384507.2224 | 99% |
| 7 | 1000000 | 1500000 | 371842.4998 | 99% |
| 7 | 2500000 | 3000000 | 432201.4475 | 99% |
| 7 | 4500000 | 5000000 | 433915.6626 | 99% |
| 7 | 9000000 | 9500000 | 375305.7718 | 99% |
| 8 | 2500000 | 3000000 | 558088.9716 | 99% |
| 8 | 3000000 | 3500000 | 511041.0465 | 99% |
| 8 | 4000000 | 4500000 | 717980.2706 | 99% |
| 8 | 8000000 | 8500000 | 492625.3961 | 99% |
| 8 | 8500000 | 9000000 | 376846.5865 | 99% |
| 8 | 10000000 | 10500000 | 420540.4231 | 99% |
| 8 | 13000000 | 13500000 | 684946.6208 | 99% |
| 8 | 15500000 | 16000000 | 502157.6461 | 99% |
| 8 | 17000000 | 17500000 | 1129351.583 | 99% |
| 8 | 18000000 | 18500000 | 490227.0721 | 99% |
| 8 | 20500000 | 21000000 | 524632.9658 | 99% |
| 8 | 22500000 | 23000000 | 486666.1792 | 99% |
| 9 | 2000000 | 2500000 | 556401.2369 | 99% |
| 9 | 5000000 | 5500000 | 775018.3994 | 99% |
| 9 | 7500000 | 8000000 | 454160.2114 | 99% |
| 9 | 8000000 | 8500000 | 936717.0165 | 99% |
| 9 | 9500000 | 10000000 | 337577.0916 | 99% |
| 9 | 11000000 | 11500000 | 977042.6019 | 99% |
| 9 | 11500000 | 12000000 | 834255.15 | 99% |
| 9 | 12500000 | 13000000 | 868990.0688 | 99% |
| 9 | 13000000 | 13500000 | 693069.5218 | 99% |
| 9 | 16000000 | 16500000 | 1243684.351 | 99% |
| 9 | 18500000 | 19000000 | 566761.3084 | 99% |
| 9 | 20500000 | 21000000 | 711910.1137 | 99% |
| 9 | 21500000 | 22000000 | 452028.0936 | 99% |
| 9 | 26500000 | 27000000 | 823689.5419 | 99% |
| 9 | 89000000 | 89500000 | 357431.9418 | 99% |
| 10 | 2000000 | 2500000 | 411156.0546 | 99% |
| 10 | 2500000 | 3000000 | 376202.5546 | 99% |
| 10 | 4500000 | 5000000 | 459430.9087 | 99% |
| 10 | 10000000 | 10500000 | 370991.6374 | 99% |
| 10 | 77000000 | 77500000 | 401028.6456 | 99% |
| 10 | 92500000 | 93000000 | 491616.1488 | 99% |

**Supplementary** **Table 9**. Detailed information KASP primers for seven recombination hotspots

| Primer name | Position | Primer direction | Primer sequence |
| --- | --- | --- | --- |
| ZG1 | chr3:215931758 | F1 | TAAAACAGGTATGGGCATAT |
|  |  | F2 | TAAAACAGGTATGGGCATAG |
|  |  | R1 | ACCACCACAGCTTATGATGG |
| ZG2 | chr3:216527768 | F1 | GAGCCTCCATGAGCGCCTTG |
|  |  | F2 | GAGCCTCCATGAGCGCCTTC |
|  |  | R1 | ATTGCTGTCAGGCCCCGCGA |
| ZG3 | chr7:8929349 | F1 | CACCACCACAACAATTCGGA |
|  |  | F2 | CACCACCACAACAATTCGGC |
|  |  | R1 | GCAGATCAAAGCTGTGCTGG |
| ZG4 | chr7:9540868 | F1 | CATATGCAGGCAGTCCTTCG |
|  |  | F2 | CATATGCAGGCAGTCCTTCA |
|  |  | R1 | TACCTGACACCACCGAAAGA |
| ZG5 | chr8:2428889 | F1 | GTCACGTCCATGTTTCCTCA |
|  |  | F2 | GTCACGTCCATGTTTCCTCG |
|  |  | R1 | TCATACGGCCTAGACCCATG |
| ZG6 | chr8:3026396 | F1 | ACAACGGTCTGGAAGGAACG |
|  |  | F2 | ACAACGGTCTGGAAGGAACA |
|  |  | R1 | GGCAATCTTTGTCTCTGACA |
| ZG7 | chr8:12989522 | F1 | ACAGGGAAATCACAGGAAAA |
|  |  | F2 | ACAGGGAAATCACAGGAAAG |
|  |  | R1 | CACGAGTTTTTCTCTGAACC |
| ZG8 | chr8:13591616 | F1 | TCCTCGTGATCTCCTCCTCC |
|  |  | F2 | TCCTCGTGATCTCCTCCTCT |
|  |  | R1 | AGACGGCAGGGGTCAAGAAG |
| ZG9 | chr8:15472970 | F1 | TAATCGGCTTTGGTGCAACA |
|  |  | F2 | TAATCGGCTTTGGTGCAACG |
|  |  | R1 | GGCAACAATCGCAATTGTCG |
| ZG10 | chr8:15991485 | F1 | CTCAGCATTTCCACGAGATT |
|  |  | F2 | CTCAGCATTTCCACGAGATC |
|  |  | R1 | AAAGACCTGACATCACTGGG |
| ZG11 | chr9:11499956 | F1 | TTATTGCCATGAACCACACT |
|  |  | F2 | TTATTGCCATGAACCACACC |
|  |  | R1 | CGATTTCTTGGAATGTGCTG |
| ZG12 | chr9:12323651 | F1 | TCGACTTCAACGCAGGCTCA |
|  |  | F2 | TCGACTTCAACGCAGGCTCT |
|  |  | R1 | CGAGTGACATGCAGTCACCC |
| ZG13 | chr10:9912303 | F1 | TCCAGACTGATCCCGTTCTG |
|  |  | F2 | TCCAGACTGATCCCGTTCTC |
|  |  | R1 | TTTGAGCAGGTTGAATGTAG |
| ZG14 | chr10:10583136 | F1 | AGACCAGCAACATGAAACCA |
|  |  | F2 | AGACCAGCAACATGAAACCG |
|  |  | R1 | CATTTTTGGGGCTTCTTTGC |
| MT1 | chr3:215936068 | F1 | GTGCACGCAAAGGCTACCGC |
|  |  | F2 | GTGCACGCAAAGGCTACCGA |
|  |  | R1 | ACCCGAATGCTCGCTCACAC |
| MT2 | chr3:216839696 | F1 | GTGACACGGAACACCGAAGA |
|  |  | F2 | GTGACACGGAACACCGAAGG |
|  |  | R1 | CGGTCGTCGATGAAAATGGC |
| MT3 | chr7:9049351 | F1 | CTGTCGGACTAACAAGGTTT |
|  |  | F2 | CTGTCGGACTAACAAGGTTG |
|  |  | R1 | CACAAGATAAGATCACGGTC |
| MT4 | chr7:9541101 | F1 | AGATACTGGGAGCGGGGTCC |
|  |  | F2 | AGATACTGGGAGCGGGGTCT |
|  |  | R1 | GATCCAAAGACCAGGTCTCG |
| MT5 | chr8:2428889 | F1 | GTCACGTCCATGTTTCCTCA |
|  |  | F2 | GTCACGTCCATGTTTCCTCG |
|  |  | R1 | TCATACGGCCTAGACCCATG |
| MT6 | chr8:3026396 | F1 | ACAACGGTCTGGAAGGAACG |
|  |  | F2 | ACAACGGTCTGGAAGGAACA |
|  |  | R1 | GGCAATCTTTGTCTCTGACA |
| MT7 | chr8:12904069 | F1 | CTCGCTTGGATGGTCCATGC |
|  |  | F2 | CTCGCTTGGATGGTCCATGT |
|  |  | R1 | GCTCATGCTTCCCTTTCAGC |
| MT8 | chr8:13442133 | F1 | TACCCGACTGCCTTGTTGCC |
|  |  | F2 | TACCCGACTGCCTTGTTGCT |
|  |  | R1 | CAGTTACTAGCACACAACGC |
| MT9 | chr8:15472948 | F1 | AGTTTTGGTGGTTGTCGCCA |
|  |  | F2 | AGTTTTGGTGGTTGTCGCCG |
|  |  | R1 | ATCCTTCGTTGCACCAAAGC |
| MT10 | chr8:15991485 | F1 | CTCAGCATTTCCACGAGATT |
|  |  | F2 | CTCAGCATTTCCACGAGATC |
|  |  | R1 | AAAGACCTGACATCACTGGG |
| MT11 | chr9:11499956 | F1 | TTATTGCCATGAACCACACT |
|  |  | F2 | TTATTGCCATGAACCACACC |
|  |  | R1 | CGATTTCTTGGAATGTGCTG |
| MT12 | chr9:12022858 | F1 | GCAAGGGGAAGGTGTGGCTT |
|  |  | F2 | GCAAGGGGAAGGTGTGGCTC |
|  |  | R1 | ATCTCGTAGACCTCGTTGGG |
| MT13 | chr10:9893726 | F1 | CACAGTAGAAGAGTGGCAAT |
|  |  | F2 | CACAGTAGAAGAGTGGCAAC |
|  |  | R1 | CTCTTTCTTGTTGTCAGGGA |
| MT14 | chr10:10599360 | F1 | ATGGTTTCGCACTGCTCCTG |
|  |  | F2 | ATGGTTTCGCACTGCTCCTA |
|  |  | R1 | TTCGCAAGCTAGATGCAACG |
| ZG is ZHENG/GEMS2 population,MT is Mo17/TY1 population. | | | |
